# Ontogenetic changes and sexual dimorphism in the cranium and mandible of the Atlantic walrus (*Odobenus rosmarus rosmarus* L.)

**DOI:** 10.1002/ar.70050

**Published:** 2025-09-12

**Authors:** Katrien Dierickx, Oliver Kersten, Youri van den Hurk, Brenna A. Frasier, Richard Sabin, Bastiaan Star, James H. Barrett

**Affiliations:** ^1^ Department of Archaeology and Cultural History NTNU University Museum Trondheim Norway; ^2^ Centre for Ecological and Evolutionary Synthesis, Department of Biosciences University of Oslo Oslo Norway; ^3^ Hokkaido University Museum Hokkaido University Sapporo Japan; ^4^ Nova Scotia Museum Halifax Nova Scotia Canada; ^5^ Vertebrates Division Natural History Museum London UK

**Keywords:** archaeology, geometric morphometrics, linear measurements, Odobenidae, osteology, sex and age determination

## Abstract

Walruses have been an important subsistence and cultural resource for humans and have been exploited for millennia across their distribution. This exploitation has contributed to severe declines in several populations and local extirpations. The study of walrus remains from archaeological sites can provide insights into past exploitation strategies and contribute to improving current conservation practices. The ability to differentiate disarticulated skeletal remains by age and sex can provide insights into past herd compositions. While sexing is a well‐established approach for mandibles, a clear morphological key to determine such differentiation in crania is currently lacking. Here, linear measurements and geometric morphometrics of mandibles (*n* = 121) and crania (*n* = 116) of the Atlantic walrus (*Odobenus rosmarus rosmarus*) are used to describe the age and sex differences. Suture fusion states are strongly associated with age. There are clear size and shape differences between adults and neonates, with juveniles showing a gradual transition between states. Sexual dimorphism is clearly present in adults, but not detected in juveniles and neonates. Linear discriminant analysis successfully assigned sexes to 19 out of 21 (90.5%) DNA‐sexed adult test specimens. Comparatively, only two out of five juvenile test specimens were correctly sexed using morphometrics. We suggest that aging specimens using suture fusion states, followed by classification between adult males and females, provides the most accurate results. This classification technique will facilitate future osteological studies on walruses, for example, to understand their ecomorphology and to provide insight into historical age‐ and sex‐based exploitation and population impacts through time.

## 1 INTRODUCTION

Walruses (*Odobenus rosmarus* [Linnaeus, 1758]) are characterized by their large size and tusks. Living in the Arctic region, these semi‐aquatic animals gather in huge groups on beaches or ice to rest and give birth. There are two recognized subspecies: *O. rosmarus rosmarus* (Linnaeus, 1758) in the Atlantic Ocean and *O. rosmarus divergens* (Illiger, 1815) in the Pacific Ocean and Laptev Sea. They have been an important subsistence and cultural resource for humans, who used their meat for food and tusks, skin and other products for tools and cultural objects, since at least 2500 to 2000 BCE in the western Atlantic Ocean (Black, 2017; Darwent & LeMoine, 2021). The exploitation of Atlantic walruses is thought to have become more intense and economically driven during the medieval and post‐medieval periods, when European exploration expanded across the North Atlantic (e.g., Barrett et al., 2020; Barrett et al., 2022; Gjertz & Wiig, 1994; Keighley et al., 2019; McLeod et al., 2014; Star et al., 2018). This intensification is thought to have contributed to the extirpation of at least two walrus populations (Iceland in the medieval period and the Maritime Provinces of Canada in the 18th century CE) and to the severe population decline of others, such as in Svalbard (Gjertz, 2021; Gjertz & Wiig, 1994; Keighley et al., 2019; Kruse et al., 2016; McCaffrey, 2020; McLeod et al., 2014; Rijkelijkhuizen, 2009). Since Atlantic walruses received protection from large‐scale economic exploitation during the 20th century, populations have slowly recovered (e.g., Gjertz & Wiig, 1994; Kovacs et al., 2014).

The study of walrus remains from archaeological sites can provide key insights into the exploitation strategies Indigenous communities and European traders employed, ultimately resulting in a better understanding of both past societies and the historical ecology of the species (e.g., Barrett et al., 2020; Desjardins, 2018; Dyke et al., 1999; Gotfredsen et al., 2018; Murray, 1992). Combined with traditional ecological knowledge (Inuit *Qaujimajatuqangit*) (e.g., Born et al., 2017; Martinez‐Levasseur et al., 2021), these insights can provide an informative comparison vis‐à‐vis modern‐day walruses and can help to inform current conservation practices. For example, walrus remains can be used to gain insight into their past abundance, ecology, distribution, and behavior, and provide a baseline with which to compare modern post‐exploitation populations.

A fundamental methodological step for zooarchaeological analyses of a species is the ability to differentiate between juvenile or adult specimens and between males and females. Analyzing the sex and age of exploited animals can reveal hunting and subsistence strategies used by humans and facilitate inferences regarding the cultural or economic significance of their products (e.g., Magnell, 2005; Weinstock, 2000). By relating the zooarchaeological remains to the history of human societies, a more in‐depth understanding of how humans have impacted these animals can be obtained (e.g., Desjardins & Gotfredsen, 2021).

There is clear sexual dimorphism in modern adult walruses, with the males being on average larger than females. Currently, differentiating between the sex and age of walruses is uncommon (e.g., Desjardins, 2013; Thompson, 2011) and is mostly done using mandibles, which is a well‐established approach (e.g., Boisville et al., 2022; Gotfredsen et al., 2018; Monchot et al., 2013). Adult males have a larger mandible, and especially wider rami compared to females (Taylor et al., 2020; Wiig et al., 2007). The distinction in adult specimens is clear enough to allow separation of males and females based on linear measurements (mandible depth and mandible thickness or mandible length and mandible thickness) (Taylor et al., 2020; Wiig et al., 2007). These morphometric analyses have successfully been applied to determine the sex of walruses from archaeological assemblages (Gotfredsen et al., 2018; Monchot et al., 2013). Mandibles from male and female adults can also be differentiated using visual characteristics. Males have a more curved anterior margin in lateral view, while this is straight in females, and in females, the anterior tip of the symphysis is more pinched and the coronoid process more angular compared to males (Mohr, 1942, cited by Boisville et al., 2022; Boisville et al., 2022; Fay, 1982). The shape of the mandible in lateral view also allows classification into males and females using geometric morphometrics (Boisville et al., 2022). However, due to the absence of sexual dimorphism in growing mandibles of immature walruses (neonates, juveniles, or subadults), these distinguishing characteristics are only applicable on mandibles of adult specimens (Taylor et al., 2020). Mandibles of immature walruses are recognized by having incompletely fused suture lines (Taylor et al., 2020).

Due to limited insight into osteological cranial differences between juveniles, adults, males, and females, sexing by cranium size and shape is less well studied (e.g., Barrett et al., 2020). On crania, the distance between the tusks is larger in males, who also have larger and sometimes straighter tusks, compared to females (Boisville et al., 2022; Fay, 1982). Male and female juveniles are very similar in size and shape, although males tend to have a slightly broader head, even just after a few months (Fay, 1982). Measurements of the tusk socket have previously been shown to allow sexing of even highly fragmented crania of adults (Barrett et al., 2020). However, little is known about shape and size differences relating to age and potential sexual dimorphism in non‐adult walruses in both crania and mandibles.

Walruses are slow‐growing animals, with females reaching their adult size around 10 years of age, while males grow until 15 years old (Fay, 1982). Many linear measurements show a strong correlation with the size or age of walruses (e.g., mastoidal width with age, mandible width with age), which is different for both sexes (e.g., McLeod et al., 2014). Various studies have assessed ontogenetic stages by using proxies based on morphological features, such as the relative size, tooth wear, irregularity on the surface of the mandibular condyle, and reduction of porosity in the bone, especially the mandibular bone (Fay, 1982; Boisville et al., 2022). Tooth eruption pattern as a proxy for age is difficult for walruses as the species has a variable number of teeth, and eruption and replacement do not always follow the same pattern (McLaughlin et al., 2022). Counting cementum increments in teeth has been used regularly to age individuals with high precision (Garlich‐Miller et al., 1993; Mansfield, 1958). However, the applicability in archaeological specimens of this approach is limited due to teeth often being missing and/or poorly preserved, and the destructive nature of the technique further constrains its wide‐scale utility. Fusion of the mandibular symphysis can be a useful tool to assess the maturity of a specimen (Taylor et al., 2020) and similar approaches have been used in past studies to isolate adult crania for stable isotope analysis (Barrett pers. comm.). However, in the absence of detail, suture line fusion only allows differentiation of fully grown adults from young juveniles; it is difficult to identify subadult specimens. While several studies have analyzed suture fusion states in relation to age in other pinnipeds (e.g., Brunner et al., 2004; Stewardson et al., 2011; Tedman, 2003), there is currently no insight into the specific ages of suture fusions for walruses, and how they might differ between males and females, as the sexes follow different growth rates (Fay, 1982).

This study will provide a morphological analysis of mandibles and crania of the Atlantic walrus (*Odobenus rosmarus rosmarus*) using suture fusion state analysis, linear morphometrics, and 3D geometric morphometrics on osteological specimens from museum collections with known sex, age class, and, in a few cases, actual ages to describe shape and size differences between different ages and sexes. Methods will then be developed to classify unknown specimens, which are tested and confirmed using DNA sexing. Improved classification will allow a better insight into zooarchaeological assemblages, resulting in a more detailed interpretation of past human hunting practices and the long‐term impacts humans have had on these animals and their ecosystems.

## 2 MATERIALS AND METHODS

### 2.1 Specimens

Morphometric analysis was performed on osteological natural history specimens of Atlantic walrus (*n* = 126) from the following museum collections: American Museum of Natural History (New York, USA), Canadian Museum of Nature (zoological, paleontology and Government of Nunavut Heritage collections) (Ottawa and Gatineau, Canada), Natural History Museum (London, United Kingdom), Naturalis Biodiversity Center (Leiden, the Netherlands), Natural History Museum (Oslo, Norway), NTNU University Museum (Trondheim, Norway; Hårsaker & Bakken, 2024), Smithsonian National Museum of Natural History (Washington D.C., USA), Tromsø Museum (The Arctic University Museum of Norway, Tromsø, Norway), The Polar Museum (Tromsø, Norway), The University Museum of Bergen (Bergen, Norway), and Zoological Museum, Natural History Museum of Denmark (Copenhagen, Denmark) collected from Canada, Greenland, Iceland, Svalbard, Franz Josef Land, and possibly Jan Mayen. Table 1 provides a summary of the specimens used in this study. Animals in these collections obtained via zoos or other captive environments were excluded from the analysis. Details on each specimen can be found in Table S22 in Data S7. For 111 specimens, both cranium and mandible were available. Specimens are identified with a unique FOC (“4‐OCEANS”) number.

**TABLE 1:** Overview of the number of Atlantic walrus specimens used in this study for linear measurement (LM) analysis and geometric morphometric (GM) analysis of crania and mandibles, grouped per sex and age group.

Ontogeny group	LM cranium	GM cranium	LM mandible	GM mandible	Total
Female (sub)adult	31	31	33	31	35
Female juvenile	9	9	8	6	9
Male (sub)adult	48	48	53	48	54
Male juvenile	17	17	17	15	17
Neonate	11	11	10	9	11
Total	116	116	121	109	126

*Note*: For more details on each specimen see Table S22 in Data S7.

**FIGURE 1:**
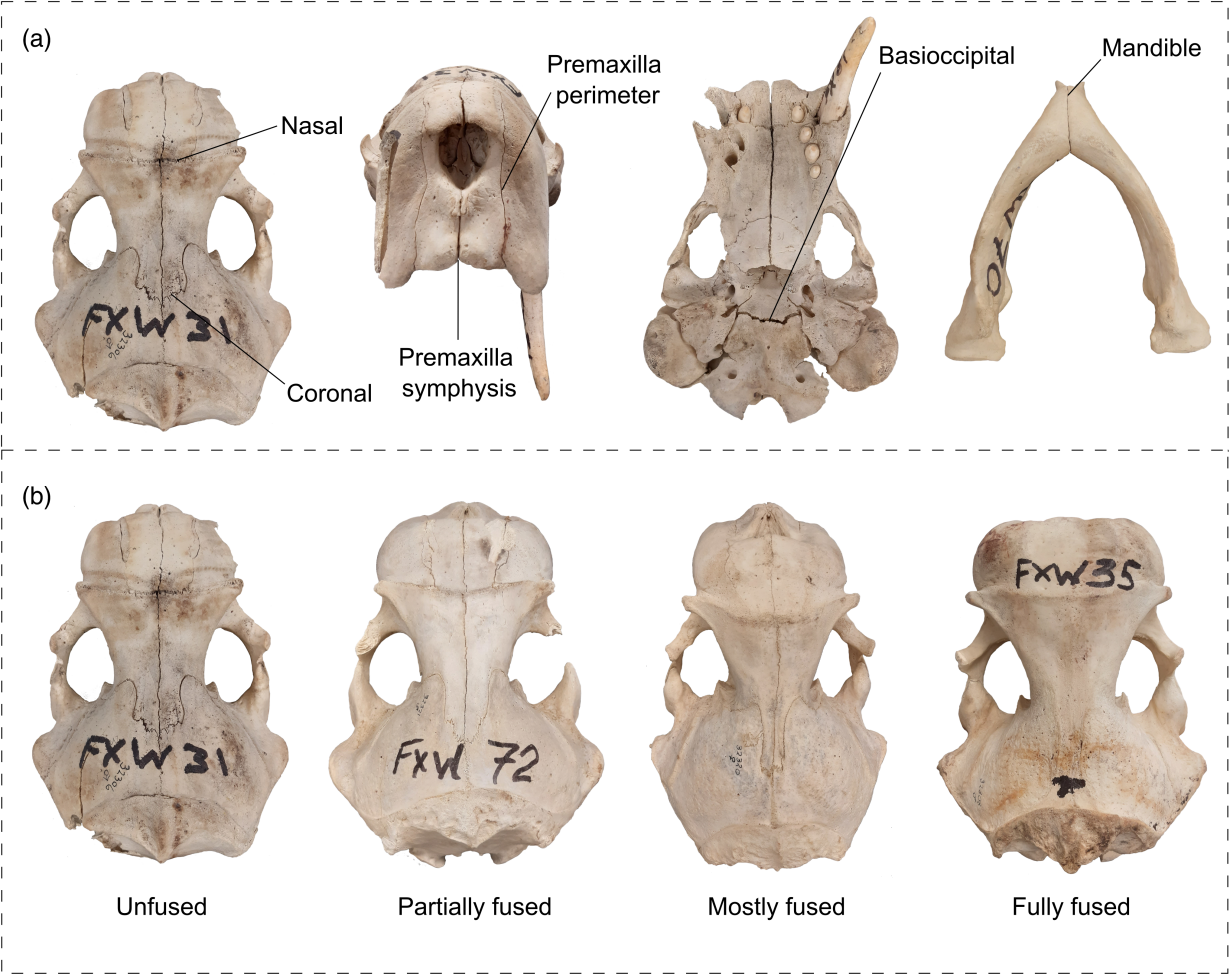
Suture fusion locations and states between Atlantic walrus specimens of different ages. (a) Location of sutures on crania (from left to right: dorsal, frontal and ventral view) and mandible (on the right, ventral view) shown on specimen FOC4507; (b) suture fusion states following Taylor et al. (2020) on crania of unfused (3.2 years old male, FOC4507), partially fused (8.3 years old male, FOC4495), mostly fused (12.3 years old female, FOC4494), and fully fused (18.2 years old male, FOC4491) sutures. Photos not to scale.

Each specimen was assigned an initial ontogenetic group, based on age (neonate, juvenile, adult) and sex (female, male). Subadults were included in the adult group for analysis (see Results section). Males and females were differentiated by associated data from the collections, or visually by the shape of their mandibles for very clear specimens, following Boisville et al. (2022) for adults, which is a well‐established approach. For subadults, juveniles, and neonates, no sex was assigned, unless it was known from museum catalogue information. In case this difference was not clear or no data were available, no sex was assigned (“unknown”) and the specimen was not included in this study, except for neonates to obtain sufficient individuals for this age group. Age was determined either by associated museum metadata, or by using fusion states following Taylor et al. (2020) and this study (see Results section) on crania (five sutures) and mandibles (one suture) (Figure 1). While Taylor et al. (2020) applied suture fusion states on walrus mandibles only, we applied the same descriptions on crania here, as these are well‐illustrated and species‐specific. Unfused suture state is defined as the bones being completely loose from each other, partially fused as the bones starting to grow together (but still showing a clear separation and sometimes being slightly loose), mostly fused as the bones being together and unmoveable while the suture line remains visible across most of its length, and fully fused as the suture line having mostly disappeared (also see Taylor et al., 2020). The coronal suture joins the parietal and frontal bones and can usually be seen very clearly from a dorsal view. The nasal suture joins the frontal and nasal bones. When fully fused, the nasal suture is often accompanied by a raised ridge in adults. There are two types of premaxilla sutures: the symphysis connecting both premaxillae and the perimeter sutures connecting the premaxilla with the maxilla. The basioccipital suture, located on the ventral side behind the nasal cavity, joining the basisphenoid and occipital, can be difficult to locate in adults. The symphysis of the mandibles can be seen ventrally at the anterior margin.

A small subset of specimens (*n* = 18), caught in 1961 in Parry Bay in northern Foxe Basin, Canada, has recorded age expressed in years in the museum metadata. Studying this subset of specimens allows for a more in‐depth assessment of the five suture fusion states as a proxy for age in crania. The defined suture fusion states were further used to classify specimens of unknown age to age groups (see Table 5).

### 2.2 Linear morphometrics

**FIGURE 2:**
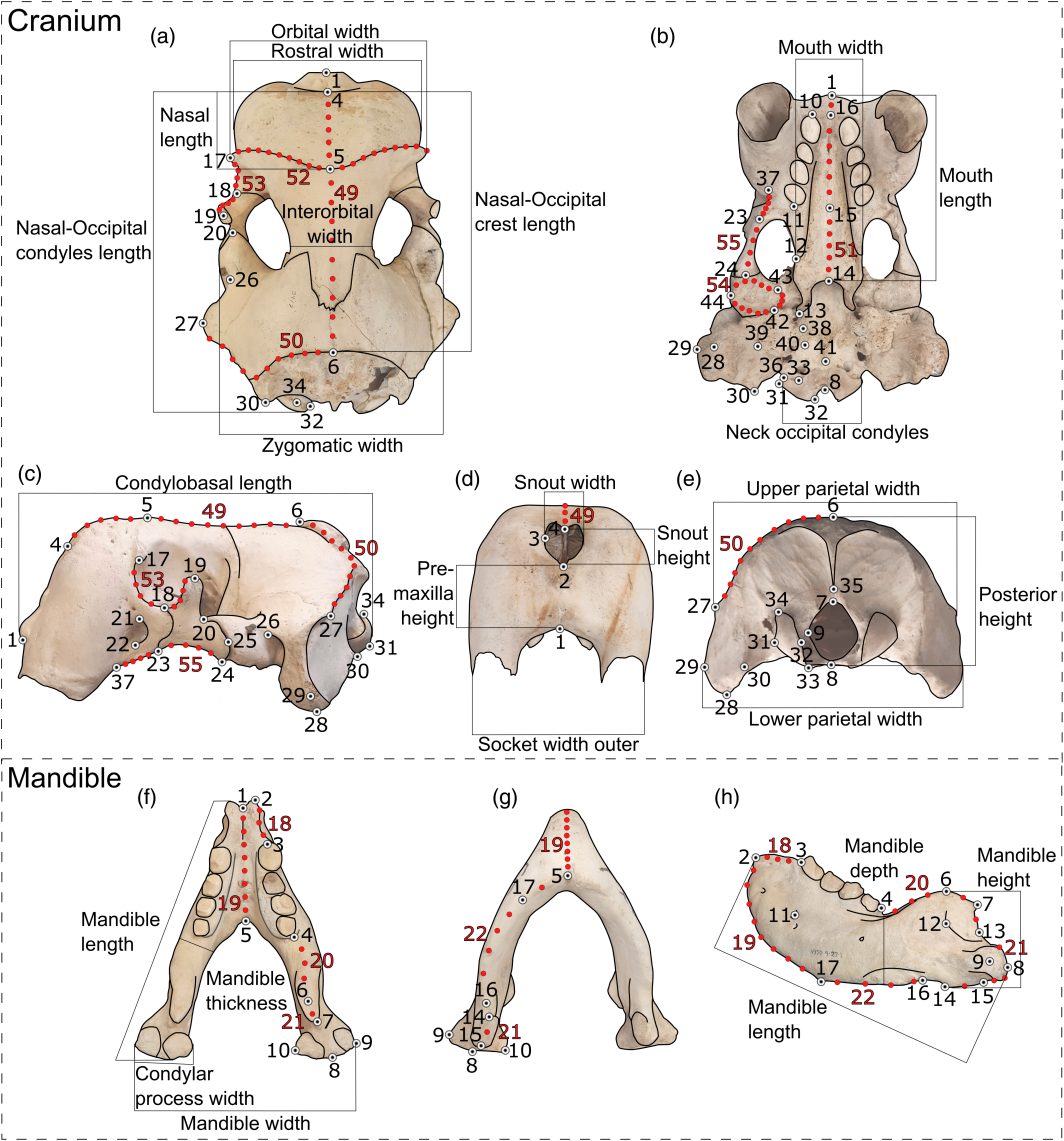
Measurements and landmark configurations on crania and mandibles of Atlantic walruses. (a) dorsal view; (b) ventral view; (c) lateral view; (d) anterior view; (e) posterior view of cranium; (f) dorsal view; (g) ventral view; (h) lateral view of mandible. Definitions of all measurements are provided in Table 2. Definitions of all landmarks are provided in Tables 3, 4. Paired measurements and landmarks are only displayed on one side. White with black dots indicate fixed landmarks and red dots sliding semilandmarks.

Two datasets were created, using 116 (complete and partial) crania, and 121 (complete and partial) mandibles. Each individual was represented by at least one of these two elements. In total, 18 linear measurements were taken on crania, and 6 on mandibles where possible using “Sylvac” external, “RS” external, and “Moore & Wright Precision” inside digital calipers accurate to 0.01 mm. Where relevant, both left and right sides were measured. These measurements follow the Committee on Marine Mammals (1967), Wiig and Gjertz (1996), Ericson and Storå (1999), Wiig et al. (2007), McLeod et al. (2014), Taylor et al. (2020), and McLaughlin et al. (2022), with additional measurements added. Abbreviations for and short explanations of these measurements are given in Table 2. Figure 2 provides a visual summary of the measurements. All measurements were present in at least 70% of the specimens across the dataset. Additionally, a subset of specimens was subjected to internal tusk socket measurements and subsequently analyzed (Data S5).

**TABLE 2:** Measurements taken on Atlantic walrus crania and mandibles (also see Figure 2) following Committee on Marine Mammals (1967), Wiig and Gjertz (1996), Ericson and Storå (1999), Wiig et al. (2007), McLeod et al. (2014), Taylor et al. (2020), McLaughlin et al. (2022) and additional measurements designed in this study.

Measurement	Abbr.	Definition	Paired	Reference
Cranium				
Rostral width	RW	Greatest width of the maxillary bones at the level of the tusks		Committee on Marine Mammals (1967)
Orbital width	OW	Width at anterior protruding tip of the frontal bone		This study
Interorbital width	IW	Least distance between the orbital fossae		Committee on Marine Mammals (1967)
Zygomatic width	ZW	Greatest width across the zygomatic arches		Committee on Marine Mammals (1967)
Nasal‐occipital condyles length	ONL	Length from the anterior tip of the nasals to the posterior of the occipital condyles		McLeod et al. (2014)
Nasal‐occipital crest length	nOc	Length from the most anterior tip of the nasals to the occipital crest		McLeod et al. (2014)
Nasal length	NL	Length from the anteriormost to posteriormost points of the nasal bones		McLeod et al. (2014)
Condylobasal length	CBL	Length from the anterior tip of the premaxilla to the occipital condyle		Committee on Marine Mammals (1967)
Mouth length	ML	Length of mouth cavity from tip of premaxilla to posterior margin of palatine		McLaughlin et al. (2022)
Mouth width	MW	Width of parietals at concave edges		This study
Neck of occipital condyles	OC	Length between the anterior constriction of the occipital condyles		This study
Snout height	SnH	Height of nasal aperture		This study
Snout width	SnW	Greatest width of nasal aperture		This study
Premaxilla height	PMH	Length from ventral tip of premaxilla to dorsal tip of premaxilla		This study
Socket width outer	SWo	Width between outer lateral edges of tusk sockets		This study
Posterior height	PH	Length from the ventral base below the posterior foramen to the occipital crest		This study
Upper parietal width	uPW	Upper cranial width near parietal bone		McLeod et al. (2014), but split in upper and lower by this study
Lower parietal width	lPW	Lower cranial width near parietal bone along the mastoid process		McLeod et al. (2014), but split in upper and lower by this study
Mandible				
Mandible length	ManL	Length from anterior point on mandible to lateral point on posterior surface of articular condyle	Yes	Wiig et al. (2007), Taylor et al. (2020)
Mandible height	ManH	Length from most dorsal point on coronoid process to most ventral point on angular process	Yes	Wiig et al. (2007), Taylor et al. (2020)
Mandible depth	ManD	Minimal distance between dorsal and ventral mandibular surfaces, posterior to the last cheek tooth	Yes	Wiig et al. (2007), Taylor et al. (2020)
Mandible thickness	ManT	Minimal lateral distance between medial and lateral mandibular surfaces, posterior to the last cheek tooth	Yes	Wiig et al. (2007), Taylor et al. (2020)
Condylar process width	PrW	Length between lateral and medial point of the condylar process	Yes	Ericson and Storå (1999)
Mandible width	MaW	Length between the lateral sides of the left and right condylar process		McLeod et al. (2014)

*Note*: Several measurements on the mandible were measured on both left and right sides.

### 2.3 3D scanning and landmarking

3D models of each specimen were created using an Einscan HX (Shining 3D). To include texture and photorealism in the model, scans were taken using Exscan software with the Rapid scan function using a light source with a resolution of 0.7 mm. Features were selected for alignment during the scans. Specimens were scanned in multiple views, which were aligned before creating an unwatertight mesh model. Small data holes were manually filled using the tangent option if required, leaving gaps from natural fragmentation and anatomical structures as is. Final models were exported as .ply files. Landmarks were placed on the models using Stratovan Checkpoint (Stratovan Corporation, 2020) manually for fixed landmarks and using a template for sliding semi‐landmarks. Landmarking was exclusively undertaken by the first author and intraperson consistency checked (Figure S1 in Data S7). Anatomical fixed landmarks (*n* = 48 for crania, *n* = 17 for mandibles) and curves with sliding semi‐landmarks (*n* = 7 for crania, *n* = 5 for mandibles) were used. The landmarks are visualized in Figure 2 and explained in Tables 3, 4. Landmark coordinates were exported as .pts files. Each file was coded with information on the individual specimen, sex, and age.

**TABLE 3:** Description of fixed and sliding landmarks placed on the 116 crania (see Figure 2a–e).

Land‐mark	Definition	Paired
Fixed		
1	Premaxilla most anterio‐ventral tip	No
2	Nasal aperture ventral point	No
3	Nasal aperture most lateral point within nasal aperture curve	Yes
4	Nasal aperture dorsal point	No
5	Midpoint or ridge between nasals and frontals	No
6	Dorsal point occipital crest suture	No
7	Foramen magnum dorsal	No
8	Foramen magnum ventral	No
9	Foramen magnum most lateral point	Yes
10	Anterior most point on alveolar margin of tooth row	Yes
11	Posterior most point on alveolar margin of tooth row	Yes
12	Posterior suture point of palatine and maxilla	Yes
13	Posterior tip of pterygoid	Yes
14	Posterior midpoint of palatine	No
15	Midpoint of maxilla at level of landmark 11	No
16	Midpoint of premaxilla at level of landmark 10	No
17	Frontal zygomatic process	Yes
18	Dorsal suture of maxilla and zygomatic	Yes
19	Zygomatic frontal process most dorsal point	Yes
20	Dorsal suture of zygomatic and temporal (squamosal)	Yes
21	Dorsal anterior point of infraorbital foramen	Yes
22	Ventral anterior point of infraorbital foramen	Yes
23	Ventral suture of maxilla and zygomatic	Yes
24	Ventral suture of zygomatic and temporal (squamosal)	Yes
25	Lateral suture of zygomatic and temporal (squamosal)	Yes
26	Lateral joining point of mastoid process and temporal (squamosal)	Yes
27	Lateral most point on temporal	Yes
28	Ventral most point of mastoid process	Yes
29	Lateral most point of mastoid process	Yes
30	Posterior most point of occipital/temporal suture	Yes
31	Lateral point of occipital condyles	Yes
32	Medial point of occipital condyles	Yes
33	Ventral point of occipital condyles	Yes
34	Dorsal point of occipital condyles	Yes
35	Ventral point of ridge occipital	No
36	Lateral nook in front of occipital condyles	Yes
37	Ventral point between zygomatic arch and tusk socket (maxilla)	Yes
38	Medial point of tympanic bulla at basisphenoid	Yes
39	Lateral point of tympanic bulla at mastoid process	Yes
40	Posterior point of small process of basisphenoid ventrally	Yes
41	Posterior point of ventral ridge on basisphenoid	No
42	Posterior medial point glenoid fossa at tympanic bulla	Yes
43	Anterior medial point of glenoid fossa at temporal	Yes
44	Lateral point of glenoid fossa at temporal	Yes
sliding semilandmarks		
49	Contour along dorsal midline starting from anterior tip of nasal (4) till occipital crest along midline (6) including nasal suture point (5)	No
50	Contour along occipital crest between dorsal tip occipital ridge (6) and widest points head (27) on both sides	Yes
51	Contour along mouth starting from lip tip (1) till posterior side palatine (14), including 15 and 16	No
52	Contour along nasal suture between frontal process (17) including nasal suture point (5)	No
53	Contour along orbit, following posterior edge of maxilla and dorsal edge of zygomatic from frontal zygomatic process (17) to zygomatic frontal process (19), via 18	Yes
54	Contour along edge of glenoid fossa, including 42, 43, 44	Yes
55	Contour along ventral edge of zygomatic arch, from posterior socket (37), along the maxilla, via (23), till ventral suture between temporal and zygomatic (24)	Yes

**TABLE 4:** Description of fixed and sliding landmarks placed on the 109 mandibles (see Figure 2f–h).

Land‐mark	Definition	Paired
Fixed		
1	Anterior most edge of symphysis	No
2	Anterior most tip on lateral side	Yes
3	Anterior most tip of tooth plane, taken on alveolar margin	Yes
4	Posterior most tip of tooth plane, taken on alveolar margin	Yes
5	Ventral edge of symphysis	No
6	Dorsal most point of coronoid process	Yes
7	Posterior most point of coronoid process	Yes
8	Posterior most point of condyloid process	Yes
9	Lateral most point of condyloid process	Yes
10	Medial most point of condyloid process	Yes
11	Anterior mental foramen	Yes
12	Anterior/ventral most point of masseteric fossa	Yes
13	Most concave/anterior point between condyloid and coronoid process	Yes
14	Anterior most point of angular process	Yes
15	Posterior most point of angular process	Yes
16	Ventral notch between ramus and angular process	Yes
17	Ventral notch on ramus (angle between anterior and ventral edge)	Yes
Sliding semilandmarks		
18	Contour between anterior tip (2) and anterior tooth plane (3)	Yes
19	Contour along anterior and posterior edge of symphysis (1, 5)	No
20	Contour along dorsal edge from posterior tooth plane (4) to mandibular notch (13), including 6, 7	Yes
21	Contour along posterior edge from mandibular notch (13) to anterior tip of angular process (14), including 8, 15	Yes
22	Contour along ventral edge from anterior tip of angular process (14) to ventral tip of symphysis (5), via anterior notch (17)	Yes

### 2.4 Analysis

All analyses were done on either all of the available specimens or on subsets containing only adults (including subadults), juveniles, females, or males. Data were analyzed using R (R Core Team (2022), version 4.3.0 (2023‐04‐21 ucrt)—“Already Tomorrow”) using the following packages: car (Fox et al., 2007), caret (Kuhn, 2008), geomorph (Adams & Otárola‐Castillo, 2013), ggplot2 (Wickham, 2016), MASS (Venables & Ripley, 2002), Morpho (Schlager, 2017), and Sjmisc (Lüdecke, 2018).

#### 2.4.1 Linear morphometrics

Descriptive statistics were obtained by assessing the mean, standard deviation, median, variance, minimum, and maximum for each measurement per ontogenetic group. This was further visualized using boxplots (Data S8). After testing for normality using Shapiro–Wilk tests and for homoscedasticity using Breusch‐Pagan tests, ANOVA or Kruskal‐Wallis tests were used to identify significant differences between ontogenetic groups. Tukey HSD and Dunn tests were employed to see which groups differed significantly. Missing values in specimens were estimated using only specimens from the same ontogenetic group with that measurement taken, which was calculated using the mutate_at() function from the dplyr package. The isosize is calculated using the arithmetic mean of all log‐transformed and centered measurements in equal weight and is used as a proxy for size for each specimen (isometric size in Baur & Leuenberger, 2011). By projecting measurements orthogonal to the isosize, it is possible to obtain the shape variables that do not contain any size effects (see Baur & Leuenberger, 2011 for more details). All measurement data were log‐transformed, centered, and transformed using isosize before obtaining shape variables from a shape Principal Components Analysis (sPCAs), following Baur and Leuenberger (2011) and their provided functions (see Rscript in the Supplementary Information). sPCAs were conducted for crania and mandibles separately to detect differences between ontogenetic groups. Allometry was tested using a MANOVA for size alone (shape ~ isosize), for group alone (shape ~ ontogeny group), and for the interaction between size and ontogenetic group (shape ~ isosize ontogeny group). Correlations between the first three PC axes and isosize were calculated using a Spearman correlation test. Pairwise Wilcoxon tests were used to check for significant differences between mean isosize of ontogenetic groups.

#### 2.4.2 Geometric morphometrics

3D landmark data were loaded into R using the import.pts() function, provided by Chatar et al. (2025). Missing landmarks were estimated per subset of ontogenetic groups using the estimate.missing() function in the package geomorph (see Arbour & Brown, 2014). Semilandmarks to slide were determined via a table in order to minimize thin‐plate spline bending energy and optimize their position along the curve. Procrustes superimposition was performed using the gpagen() function in geomorph, which also allowed the calculation of the centroid size for each specimen, followed by a PCA using gm.prcomp() in geomorph. The centroid size (the square root of the sum of squared distances of all the landmarks of an object from their centroid) is a commonly used proxy for size in geometric morphometric analyses (Bookstein, 1991; Monteiro, 1999). Minimum and maximum shapes were visualized using the specimens with lowest or highest scores on the first three PC axes using the deformGrid2d() function in Morpho. Allometry was tested using multivariate regression (Procrustes ANOVA, procD.lm() in geomorph) for size alone (shape ~ centroid size), for group alone (shape ~ ontogeny group), and for the interaction between size and ontogenetic group (shape ~ centroid size ontogeny). Correlations between the first three PC axes and the natural logarithm of the centroid size were calculated using a Spearman correlation test. Pairwise Wilcoxon tests were used to check for significant differences between the means of the centroid size of ontogenetic groups. Data were visualized using ggplot() and associated functions.

#### 2.4.3 Linear discriminant analysis

A linear discriminant analysis (LDA) was performed to assess the success rate of classifying specimens to ontogenetic groups using either linear measurements (LMs) or geometric morphometrics (GMs). For each subset of the dataset, an LDA was performed 100 times, and the mean accuracy was calculated. Each LDA consisted of a training and testing set with an 80:20 ratio. Linear measurements were log10‐transformed, and the lda() function from the package MASS was used. The method used for LDA on geometric morphometrics here follows Dierickx et al. (2023). A PCA was performed on PC scores of generalized procrustes analysis (GPA)‐transformed landmarks of the training set. The resulting scores were used to create a training model. After standardization of the testing set using the mean shape of the PCA of the training set, the testing set is transformed into PC scores. Using the training model, the ontogenetic group is then predicted, and accuracy is calculated by comparing the prediction with the actual classification. Pooled variance analysis following Bucchi et al. (2024) is used to assess the most informative landmarks.

Additional specimens (*n* = 29 total, *n* = 15 for crania, *n* = 22 for mandibles; Table S22 in Data S7) with no metadata on sex from Canadian Museum of History (Ottawa, Canada), New Brunswick Museum (Saint John, Canada), Government of Nunavut Heritage Collections (Gatineau, Canada), Nova Scotia Museum (Halifax, Canada), Sable Island Institute (Halifax, Canada), and Svalbard Museum (Longyearbyen, Svalbard) were selected to test the classification on specimens with varied morphological states and geographical origins within the Atlantic Ocean. They were aged according to their suture fusion states. These specimens were individually analyzed against the reference set using LDA to identify the most probable sex, which was then confirmed with DNA analysis (see below). Linear measurements or landmarks that were not present in these specimens were removed from the reference set, as the LDA is not possible with missing values or landmarks. Linear measurements were log10‐transformed, and the lda() function from the package MASS was used. For geometric morphometrics, the method in Dierickx et al. (2023) was adapted. GPA and PCA were performed using procSym() combining the archaeological sample and the reference dataset. To condense the data for ease of analysis and to reduce the computational time, a PCA using the prcomp() function on the PC scores of the first PCA was performed using only those of the reference set. The PC scores of the archaeological sample after the initial GPA and PCA were used to calculate the sex using the LDA training model. For each specimen, the most probable sex was noted for the classification.

#### 2.4.4 DNA sexing

DNA sexing was applied in two ways. First, one specimen, FOC3018, was analyzed as it was an outlier in the initial data exploration and showed that the initial museum label was incorrect. Second, a test group of 29 specimens of unknown or unclear sex was analyzed to test the LDA‐based classification and thus the success rate of the morphological approach. Specimens were sampled either by cutting off a piece at the tympanic bulla or tusk socket for crania. This was done using a 38 mm diameter rotating diamond blade. For mandibles, powder was collected from a tooth alveolus using a power drill fitted with a 1.6 to 4.8 mm drill bit. Around 300 mg of bone material was taken for DNA analysis and the chunks were crushed into smaller pieces.

All pre‐PCR (polymerase chain reaction) laboratory work was performed in a clean lab at the University of Oslo, Norway, following strict ancient DNA (aDNA) guidelines (Cooper & Poinar, 2000; Gilbert et al., 2005). Prior to milling bone samples to a coarse powder using a stainless‐steel mortar (Gondek et al., 2018), samples were subjected to UV light exposure. The subsequent DNA extraction process included a modified pre‐digestion protocol (Boessenkool et al., 2017; Lord et al., 2022) using ca. 100 mg of bone powder, followed by a 48‐h overnight digestion in a lysis buffer containing EDTA (0.5 M, pH 8), urea (1 M) and proteinase K (10 ug/uL). DNA was concentrated and extracted using Amicon−30 kDA centrifugal filter units, filtered via MinElute (Qiagen) columns, and eluted in 100 μL of preheated (60°C) elution buffer (EB) (Star et al., 2014). Qubit measurements were conducted on all DNA extracts to optimize downstream protocols. Genomic libraries were built with the Santa Cruz reaction protocol (Kapp et al., 2021) and indexed using sample‐specific P5 and P7 adapters. The resulting libraries were cleaned and purified using AMPure® XP beads (Beckman‐Coulter) and subsequently sequenced on the Illumina NovaSeq 6000 platform.

Sequencing reads for each sample were processed and mapped to the reference genome (Oros_1.0_HiC, Foote et al., 2015, Dudchenko et al., 2017, 2018) using PALEOMIX (Schubert et al., 2014). Prior to mapping, the reference assembly was altered and improved in three ways: (a) Only the first 17 HiC_scaffolds were used as the reference genome due to the clear chromosome boundaries visible in the HiC contact map (https://www.dnazoo.org/assemblies/odobenus_rosmarus) and because additional scaffolds were characterized by a substantial dropoff in length, (b) The autosomes and allosomes of the reference genome were differentiated by synteny with the California sea lion (*Zalophus californianus*) reference assembly (mZalCal1.pri.v2, GCF_009762305.2), and (c) The mitogenome (NC_004029.2) was added to the reference genome.

Each sample was sexed genomically following a previously established and tested approach (Barrett et al., 2020; Barrett et al., 2022; Bro‐Jørgensen et al., 2021; Nistelberger et al., 2019) with several modifications. Specifically, a reference dataset containing the samples with more than 100,000 uniquely mapped reads (21 of 29 samples + FOC3018) was created. The sex of the individuals within this reference dataset was assigned by utilizing the ratio of “genomic coverage of X‐chromosome vs. median genomic coverage of autosomes” (X:A ratio, Males ca. 0.5, Females ca. 1.0). BAM files of the reference dataset were randomly subsampled (without replacement) to contain a final number of mapped reads ranging from 1000 to 50,000 in increments of 1000 reads. Subsequently, the ratio of “genomic coverage of X‐chromosome vs. median genomic coverage of autosomes” (X:A ratio), “genomic coverage of Y‐chromosome vs. median genomic coverage of autosomes” (Y:A ratio), and “genomic coverage of Y‐chromosome vs. genomic coverage of X‐chromosome” (Y:X ratio) was calculated for each of the 50 resulting BAM files per reference sample. The three ratios were then determined for each sample not in the reference dataset (*N* = 8) and projected onto the plot with the downsampled reference dataset to assign the genomic sex.

### 2.6 Specimen availability and ethic statement

All walrus specimens included in this study were collected by or donated to museums or registered research institutes and can be accessed. No live animals were collected by the authors involved in this study. Sampling and/or imaging permissions were kindly provided by the American Museum of Natural History, Canadian Museum of History, Canadian Museum of Nature, Government of Nunavut Heritage Collections (Department of Culture and Heritage), Inuit Heritage Trust, Natural History Museum (London), Naturalis Biodiversity Center, Natural History Museum (Oslo), New Brunswick Museum, Nova Scotia Museum, NTNU University Museum, Sable Island Institute, Smithsonian National Museum of Natural History, Svalbard Museum, The Arctic University Museum of Norway, The Polar Museum (Tromsø), The University Museum of Bergen, and the Zoological Museum, Natural History Museum of Denmark. Samples were shipped across borders (from Canada, the Netherlands and the United Kingdom to Norway) under institutional CITES registrations CA 003, CA 004, CA 020, CA 039, GB 001, NL 001, NO 001, and NO 007.

## 3 RESULTS

### 3.1 Age estimation using suture fusion

Based on the known age specimens from Parry Bay, walruses from this population that are younger than 4 years (*n* = 2) have unfused sutures and were labeled as neonates, and those older than 4 years have partially, mostly, or fully fused sutures. Due to a lack of specimens between ages 3 and 7, it is unclear when fusion of sutures sets in. Walruses older than 10 years have mostly fused or fully fused sutures and were labeled as adults or subadults. Juveniles were identified as specimens between neonates and (sub)adults with most of the sutures being partially fused. Although the majority of sutures are partially fused in juvenile walruses between ages 7 and 10, occasionally sutures can also be mostly fused. The difference between partially and mostly fused sutures is, however, prone to subjective interpretation. The coronal suture becomes mostly fused or fully fused after 13 years. The nasal suture is fully fused in all specimens older than 15 years but can occur earlier. The premaxilla sutures are partially fused until 8 years, and full fusion is present in all specimens older than 13 years. The perimeter sutures can sometimes be more fused than the symphysis suture. The basioccipital remains partially fused until age 8 and rapidly becomes fully fused in specimens older than 10 years, at which point the individual specimen can be labeled as adult. The mandible can be mostly or fully fused after 8 years (see Table 5; see also Table S1 in Data S7). Although no males with known age between 9 and 13 were available, all males older than 15 years have fully fused sutures, and those from 13 years (*n* = 2) had mostly fused sutures. Females from 11 and 12 years had mostly fused sutures, and all three females of ages 22 years and older still had some mostly fused coronal or mandible sutures (see Table S1).

**TABLE 5:** Summary of the relation between age, age group, and suture fusion state for the subset of Foxe Basin Atlantic walrus specimens for the five sutures.

Age (years)	Age group	Coronal	Nasal	Premaxilla	Basioccipital	Mandible
<3–4	Neonate	Unfused	Unfused	Unfused	Unfused	Unfused
<8–9	Juvenile	Partially fused	Partially fused	Partially fused	Partially fused	Partially fused
8–10	Juvenile	Partially fused	Partially fused	Partially fused	Partially fused	Mostly fused
10–13	(Sub)adult	Partially/fully fused	Mostly/fully fused	Mostly fused	Fully fused	Mostly fused
13–15	Adult	Mostly/fully fused	Mostly/fully fused	Fully fused	Fully fused	Mostly/fully fused
>15	Adult	Mostly/fully fused	Fully fused	Fully fused	Fully fused	Mostly/fully fused

*Note*: For details, see Table S1.

### 3.2 Morphological differences related to age and sex

#### 3.2.1 General ontogeny

**FIGURE 3:**
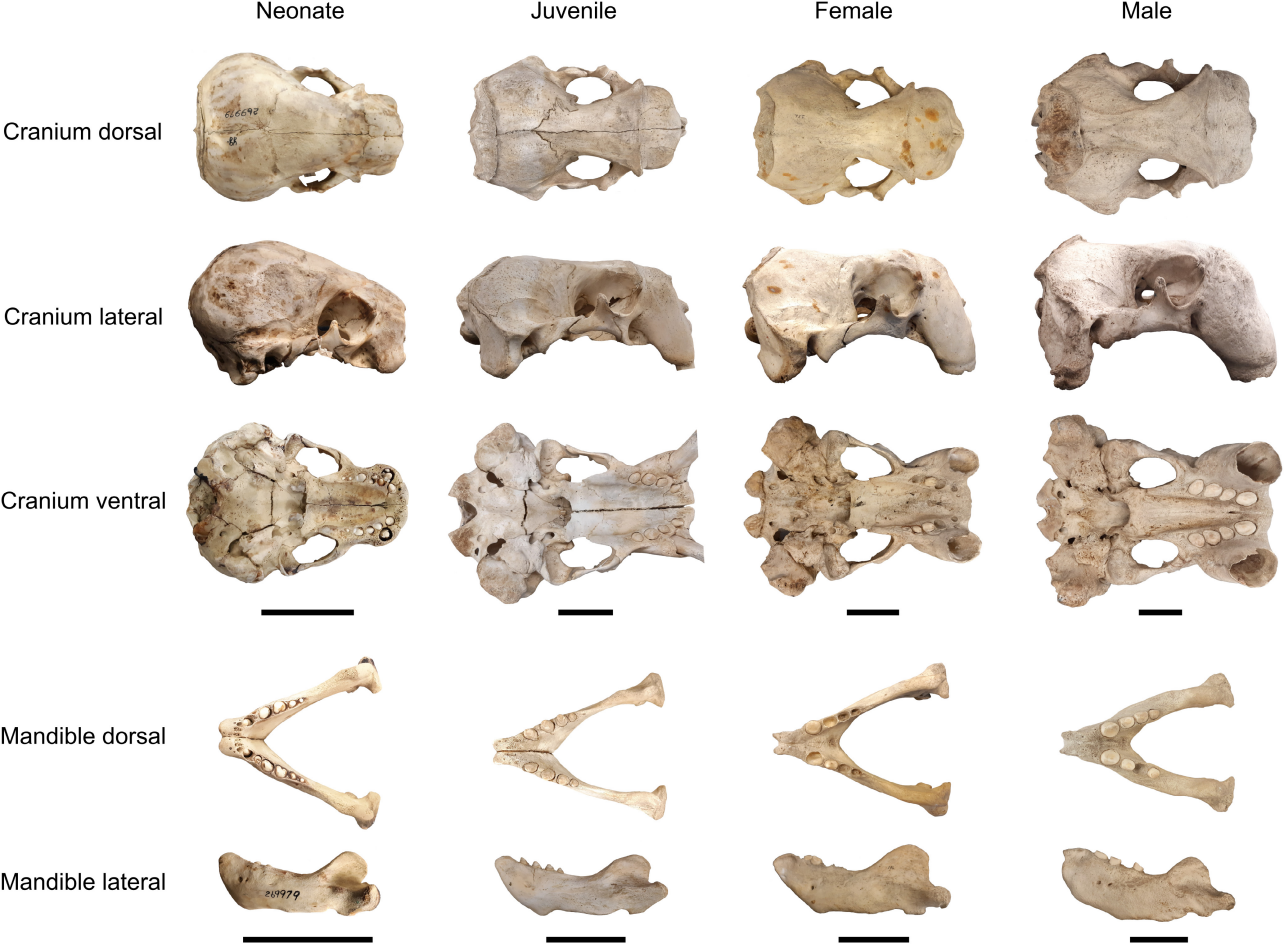
Cranium and mandibles from examples from the different age and sex groups from centrally in the convex hulls for each distinguishable ontogenetic group of Atlantic walrus in Figures 4 and 6. From left to right: neonate (FOC4916), juvenile (FOC4834; male), adult female (FOC4595), and adult male (FOC4817). From top to bottom: cranium dorsal, cranium dextral, cranium ventral, mandible dorsal, mandible sinistral view. Black bars indicate 10 cm.

A summary of descriptive statistics for each linear measurement per ontogenetic group is provided in Table S2 in Data S7 for cranium data and Table S3 in Data S7 for mandibular data. Subadults are included in the adult group as their basioccipitals and other sutures are mostly to fully fused, making the distinction between these groups difficult. As most of the measurements lacked a normal distribution and/or homoscedasticity, a Kruskal‐Wallis test was performed to test if ontogenetic groups differ significantly by linear measurements. For IW and SnH, an ANOVA was performed as these measurements are normally distributed and have homoscedasticity. Where *p* < 0.01, it was considered that the ontogenetic groups differed significantly for a measurement. All cranium and mandible measurements were significantly different between groups. Using post‐hoc tests, it was found that adult males differ from all adult and juvenile females in most measurements, except SnH and SnW in the cranium. They also differ in many measurements from juvenile males and neonates. Between the other ontogenetic groups, few significant differences in measurements were found for crania or mandibles (Table S16 in Data S6). All measurements are highly correlated with isosize, with different ontogenetic groups having different correlation coefficients (Table S17 in Data S6). Figure 3 shows the osteological differences between representative specimens from adult males, adult females, juveniles, and neonates.

**FIGURE 4:**
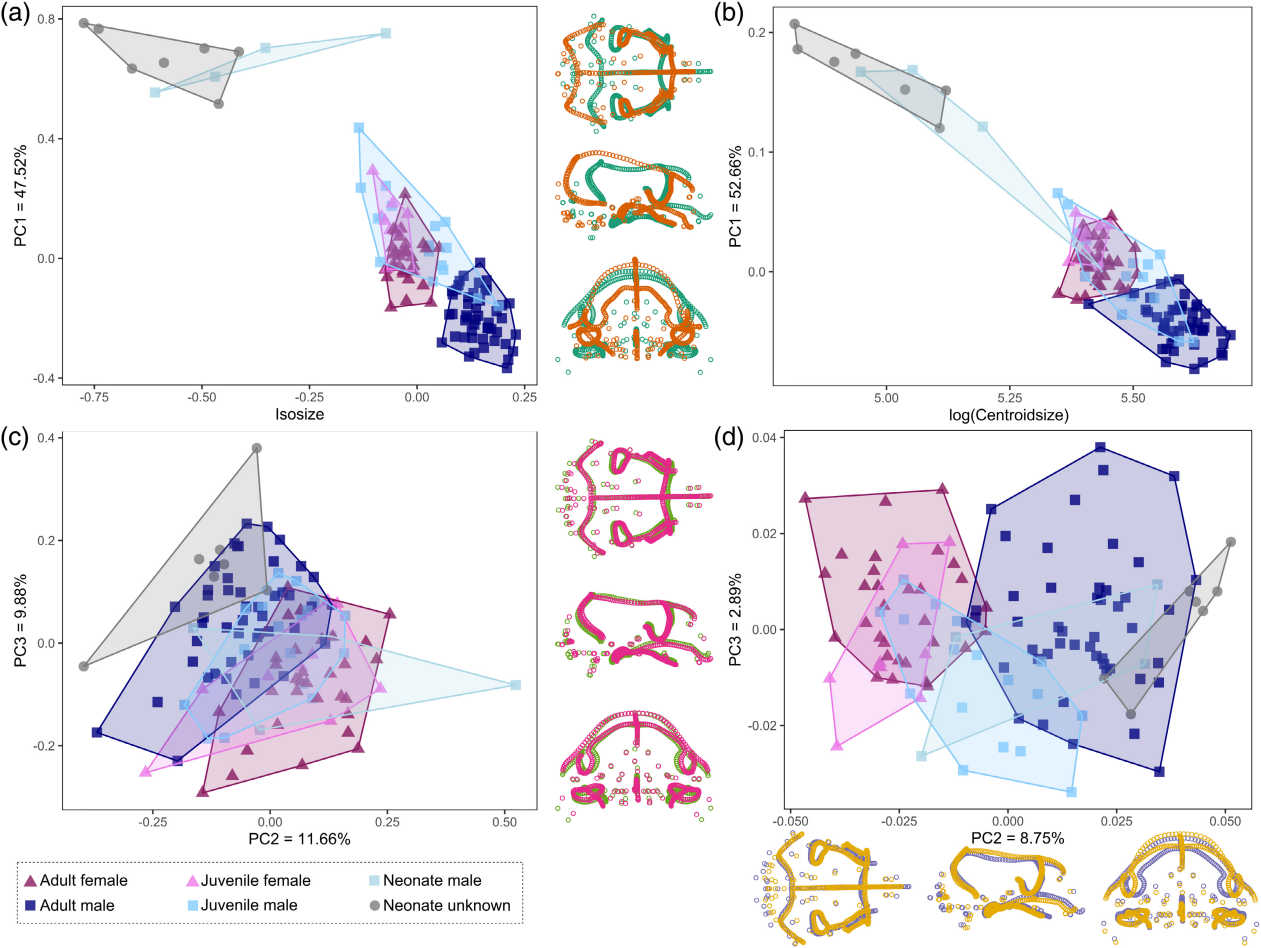
General ontogeny of the cranium between age and sex groups of Atlantic walrus specimens (*n* = 116) using linear measurements (a, c) and geometric morphometrics (b, d). (a) Isosize versus sPC1; (b) centroid size versus PC1; (c) sPC2 versus sPC3; (d) PC2 versus PC3. (b) and (d) with minimum (green, purple) and maximum (orange, pink) shape deformations per PC. For loadings of the sPC, see Figure 5.

In crania, there is a clear difference in size between the ontogenetic groups. Neonate walruses are (predictably) much smaller compared to juveniles and adults (*p* < 0.01, Figure 4a,b Table 8). Adult males are significantly larger than all other groups (*p* < 0.01). There is no significant difference in size between adult and juvenile females (isosize *p* = 0.02197; centroid size *p* = 0.09942). Juvenile males are not significantly different in size compared to adult and juvenile females (isosize *p* = 0.12457, *p* = 0.09765 respectively; centroid size *p* = 0.09623, *p* = 0.07936 respectively), but are significantly smaller than adult males (*p* < 0.01). Crania also show a strong allometric effect for linear (*F* = 155.95, *p* < 0.01; Table 6) and geometric morphometrics (*R* ^2^ = 0.47, *F* = 100.04, *p* = 0.01; Figure S2 and Table 7). A significant interaction was found between isosize and ontogenetic groups (*F* = 8.37, *p* = 0.01; Table 6) and centroid size and ontogenetic groups (*R* ^2^ = 0.032, *F* = 1.6214, *p* = 0.02; Figure S2 and Table 7). These findings indicate that the shape of ontogenetic groups is more different than expected based on size alone.

**TABLE 6:** Allometric effect across and between ontogenetic groups of Atlantic walrus using MANOVA models.

Model	Factor	*F*	Pillai	df	den df	*p*
Cranium						
Shape ~ isosize		155.95	0.80685	1	112	<0.01
Shape ~ ontogenetic group		20.613	1.512	5	330	<0.01
Shape ~ isosize ontogenetic group	Size	391.27	0.92005	1	102	<0.01
	Group	15.94	1.30173	5	312	<0.01
	Size:group	8.37	0.86100	5	312	<0.01
Mandible						
Shape ~ isosize		32.553	0.4614	1	114	<0.01
Shape ~ ontogenetic group		10.514	0.81371	4	339	<0.01
Shape ~ isosize ontogenetic group	Size	103.722	0.74590	1	106	<0.01
	Group	10.160	0.82026	4	324	<0.01
	Size:group	2.294	0.23495	4	324	<0.01

Abbreviations: den df = degrees of freedom associated with the model errors; df, degrees of freedom; *F*, F statistic; Pillai, Pillai's Trace.

**TABLE 7:** Allometric effect across and between ontogenetic groups of Atlantic walrus using Procrustes ANOVA models.

Model	Factor	*F*	*Z*	df	*r* ^2^	*p*
Cranium						
Shape ~ log centroid size		100.04	2.9259	1	0.46739	0.01
Shape ~ ontogenetic group		24.046	4.5945	5	0.52221	0.01
Shape ~ log centroid size ontogenetic group	Size	120.0392	2.9534	1	0.46739	0.01
	Group	4.9368	5.9396	5	0.09611	0.01
	Size:group	1.6214	3.0354	5	0.03157	0.02
Mandible						
Shape ~ log centroid size		42.616	4.2681	1	0.28484	0.01
Shape ~ ontogenetic group		11.442	5.2559	5	0.3571	0.01
Shape ~ log centroid size ontogenetic group	Size	48.4578	4.3691	1	0.28484	0.01
	Group	3.3303	4.8714	5	0.09788	0.01
	Size:group	1.6033	2.8535	5	0.04712	0.01

Abbreviations: df, degrees of freedom; *F*, F statistic; *r* ^2^ = r‐squared; *Z* = Z score.

**FIGURE 5:**
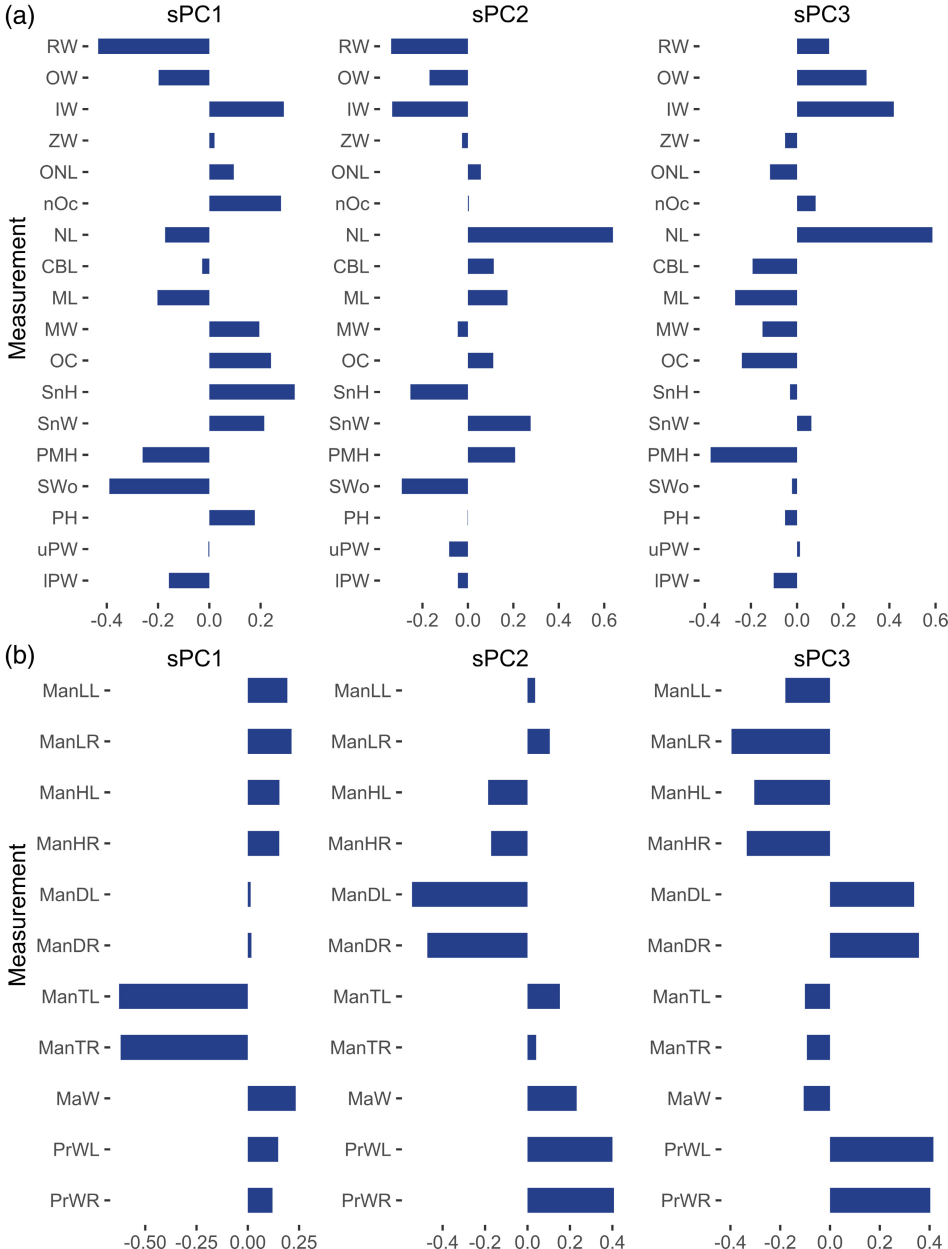
Loading contributions of the shape principal components for linear measurements using all ontogenetic groups of Atlantic walrus. (a) Cranium measurements; (b) mandible measurements.

In crania, there is a clear difference in shape between the ontogenetic groups using linear measurements (*p* < 0.01; Table 6). Neonate crania differ from adult male crania on sPC1 (Figure 4). Adult and juvenile females differ slightly from adult males on sPC1. Juvenile males also overlap slightly with adult males on sPC1. Adult and juvenile females overlap completely with juvenile males on sPC1. sPC1 (47.52%) is heavily correlated with isosize (cor = −0.84, *p* < 0.01), indicating there is allometric variation. The most important loadings on sPC1 are RW, SWo, SnH, nOc, and IW (Figure 5). sPC2 (11.66%) is not significantly correlated with isosize (cor = −0.23, *p* = 0.01162). The most important loadings on sPC2 are NL, IW, RW, SWo, and PMH. sPC3 (9.88%) is significantly correlated with isosize (cor = 0.35, *p* < 0.01). The most important loadings on sPC3 are NL, IW, PMH, OW, and ML. Based on the most important loadings for PC1, neonates differ from juveniles and adults by having relatively slenderer rostra and larger nasal apertures (Figures 4 and 5).

In crania, there is a clear difference in shape between the ontogenetic groups using geometric morphometrics (*p* < 0.01; Table 7). Neonate crania differ from juvenile and adult crania on PC1 (Figure 4). Adult and juvenile female walruses differ from adult males on PC1 and PC2. Juvenile males slightly differ from adult and juvenile females and adult males on PC2 and PC3, but still show overlap. PC1 (52.66%) is correlated with size (cor = −0.85, *p* < 0.01). Neonate walruses, which have high PC1 scores, have smaller rostra, shorter palates, more globular posterior (parietal, temporal, and occipital) crania, and narrower crania compared to adults and juveniles, that have lower PC1 scores. The oldest known (3.2 years male) specimen with unfused sutures, making it classified as neonate, falls in the juvenile male group and also has a more elongated cranium. PC2 (8.75%) is correlated with size (cor = 0.43, *p* < 0.01). Juvenile and adult females have lower PC2 scores and have relatively slender yet long crania compared to adult males that have higher PC2 scores. PC3 (2.89%) is not significantly correlated with size (cor = −0.05, *p* = 0.5739). PC3 shows the slight morphological differences between juveniles and adults. Younger walruses with lower PC3 scores have slightly narrower crania with less pronounced parietal bones compared to older walruses that have higher PC3 scores (Figure 4).

**FIGURE 6:**
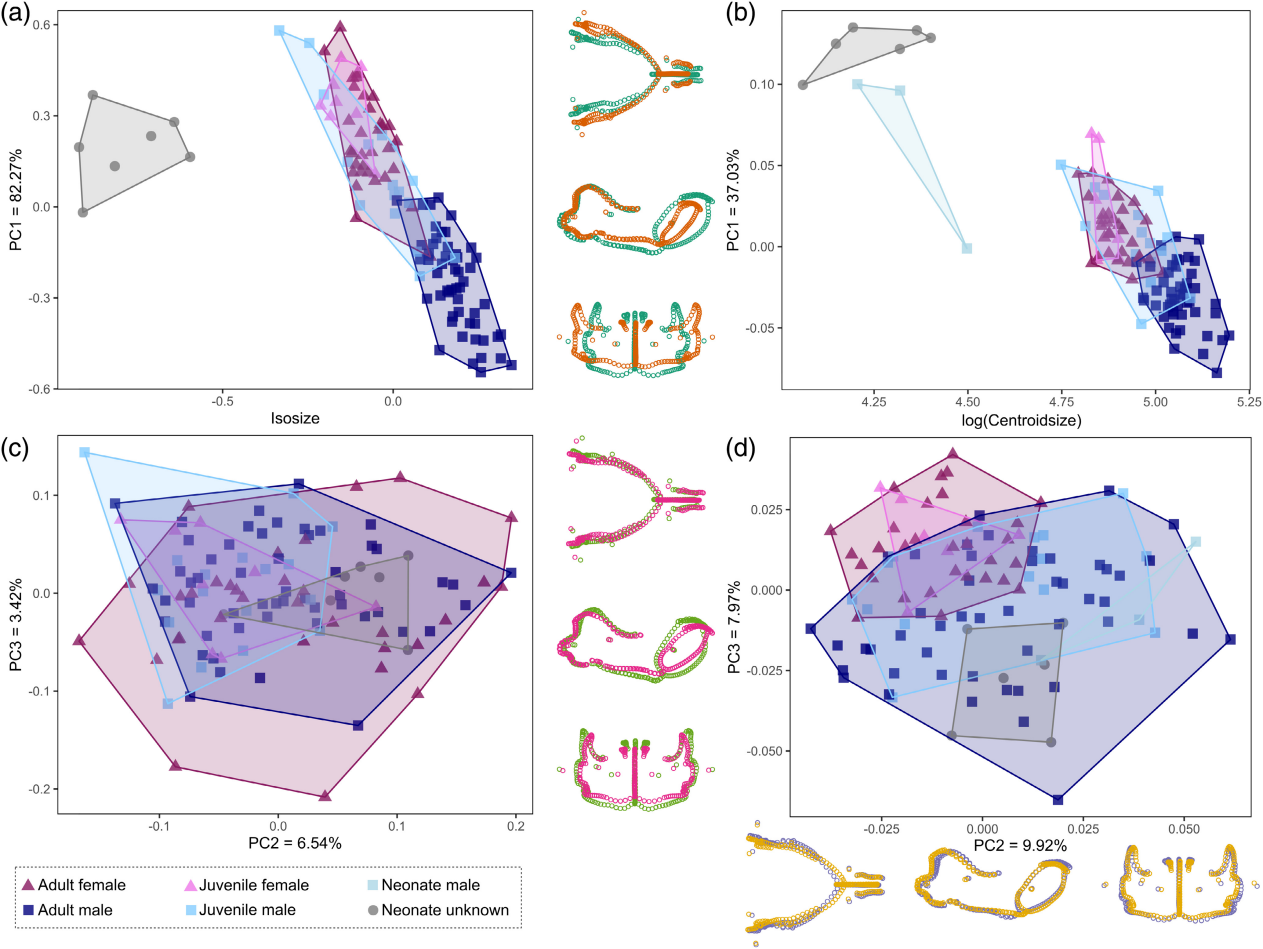
General ontogeny of the mandible between age and sex groups of Atlantic walrus specimens using linear measurements (a, c; *n* = 121) and geometric morphometrics (b, d; *n* = 109). (a) Isosize versus sPC1; (b) centroid size versus PC1; (c) sPC2 versus sPC3; (d) PC2 versus PC3. (b) and (d) with minimum (green, purple) and maximum (orange, pink) shape deformations per PC. For loadings of the sPC, see Figure 5.

In mandibles, there is a clear difference in size between the ontogenetic groups. Neonate walruses are much smaller compared to juveniles and adults (*p* < 0.01, Figure 6, Table 8). Adult males are significantly larger than all other groups (*p* < 0.01). Adult females are significantly larger than juvenile females (*p* = 0.00877) in isosize, but not in centroid size (*p* = 0.08547). Juvenile males overlap in size with adult and juvenile females (isosize *p* = 0.07126, *p* = 0.02501 respectively; centroid size *p* = 0.02439, *p* = 0.05176 respectively) and are smaller than adult males (*p* < 0.001). Mandibles also show a strong allometric effect (isosize *F* = 32.553, *p* < 0.01; Table 6; centroid size *R* ^2^ = 0.28484, *F* = 42.616, *p* = 0.01; Figure S2 and Table 7). A significant interaction was found between isosize and ontogenetic groups (*F* = 2.294, *p* < 0.01; Table 6) and centroid size and ontogenetic groups (*R* ^2^ = 0.04712, *F* = 1.6033, *p* = 0.01; Figure S2 and Table 7). These findings indicate that the shape of ontogenetic groups is more different than expected based on size alone.

**TABLE 8:** *p*‐Values of pairwise Wilcoxon tests to compare the isosize from linear measurements and centroid size from geometric morphometrics between ontogenetic groups of Atlantic walrus.

Pairs	Cranium		Mandible	
	Isosize	Centroid size	Isosize	Centroid size
Female adult—female juvenile	0.02197	0.09942	0.00877	0.08547
Female adult—male adult	<0.001	<0.001	<0.001	<0.001
Female adult—male juvenile	0.12457	0.09623	0.07126	0.02439
Female adult—male neonate	0.00014	0.03489	NA	0.00063
Female adult—unknown neonate	<0.001	<0.001	<0.001	<0.001
Female juvenile—male adult	<0.001	<0.001	<0.001	<0.001
Female juvenile—male juvenile	0.09765	0.07936	0.02501	0.05176
Female juvenile—male neonate	0.02448	0.14825	NA	0.02976
Female juvenile—unknown neonate	0.00029	0.00033	0.00044	0.00361
Male adult—male juvenile	<0.001	<0.001	<0.001	<0.001
Male adult—male neonate	<0.001	<0.001	NA	0.00021
Male adult—unknown neonate	<0.001	<0.001	<0.001	<0.001
Male juvenile—male neonate	0.00602	0.01504	NA	0.00368
Male juvenile—unknown neonate	<0.001	<0.001	<0.001	<0.001
Male neonate—unknown neonate	0.16364	0.11688	NA	0.38095

*Note*: Comparisons of crania are shown above the diagonal and those of mandibles below the diagonal.

In mandibles, there is a clear difference in shape between the ontogenetic groups using linear measurements (*p* < 0.01; Table 6). Neonate mandibles differ from adult male mandibles on sPC1 using linear measurements (Figure 6). Adult and juvenile females differ from adult males on sPC1. Juvenile males overlap slightly with adult males on sPC1. Adult and juvenile females overlap completely with juvenile males on sPC1. There is complete overlap between all groups on sPC2 and sPC3. sPC1 (82.27%) is heavily correlated with isosize (cor = −0.88, *p* < 0.01), indicating there is allometric variation. The most important loadings on sPC1 are ManT, MaW, and ManL (Figure 5). sPC2 (6.54%) is not significantly correlated with isosize (cor = −0.05, *p* = 0.5617). The most important loadings on sPC2 are ManD, PrW, and MaW. sPC3 (3.42%) is not significantly correlated with isosize (cor = 0.07, *p* = 0.4452). The most important loadings on sPC3 are ManL, ManT, and ManD. Adult males differ from all other groups by having a thicker ramus (Figures 5 and 6).

In mandibles, there is a clear difference in shape between the ontogenetic groups using geometric morphometrics (*p* < 0.01; Table 7). Neonate mandibles differ from juvenile and adult mandibles on PC1 (Figure 6). Adult and juvenile female walruses differ slightly from adult males on PC1. Adult males differ from male and female juveniles and adult females on PC3, but still show overlap. PC1 (37.03%) is correlated with size (cor = −0.81, *p* < 0.01). Neonate walruses, which have high PC1 scores, have a wider angle between the mandibles, smaller symphyses, and less pronounced ventral margin of the mandibles compared to adults and juveniles that have lower PC1 scores. The oldest known (1.2 years male) specimen with unfused sutures, making it classified as neonate, falls in the juvenile/adult male group. PC2 (9.92%) is not significantly correlated with size (cor = 0.17, *p* = 0.08299). Some adult and juvenile males have higher PC2 scores and have a slightly lower angle between the mandibles and proportionally longer rami compared to other males and adult and juvenile females that have higher PC2 scores. PC3 (7.97%) is correlated with size (cor = −0.29, *p* = 0.00209). Some adult males have lower PC3 scores and have larger symphyses and a wider angle between mandibles compared with other adult males, juvenile males, and females that have higher PC3 scores (Figure 6).

Shape differences cannot be analyzed in‐depth in relation to specific ages using the Parry Bay subset due to the small sample sizes for each group, but a visual summary showing the relationship between isosize, centroid size, sPC1, and PC1 with age for crania and mandibles is provided in Figures S5 and S6.

#### 3.2.2 Ontogenetic series per sex

Juveniles and adults were compared to assess ontogenetic shape differences with males and females separately. In both males and females, there is a strong allometric effect for crania and mandibles (Tables S4–S7). Juvenile and adult females are similar in size and shape (Figures S7–S10; Tables S4 and S5). Juvenile and adult males differ significantly in size and show clear differences in shape (Figures S11–S14; Tables S6 and S7). In their crania, juvenile males have more slender rostra, larger nasal apertures, more posteriorly positioned occipital ridges, smaller mastoid processes, shorter occipital condyles, more slender orbitals, and generally a lower cranium compared to adult males. The mandibles of juvenile males have a more slender mandibular ramus, a smaller symphysis, a lower mandible, and a lower angle between both mandibles compared to adult males.

#### 3.2.3 Sexual dimorphism per age group

**FIGURE 7:**
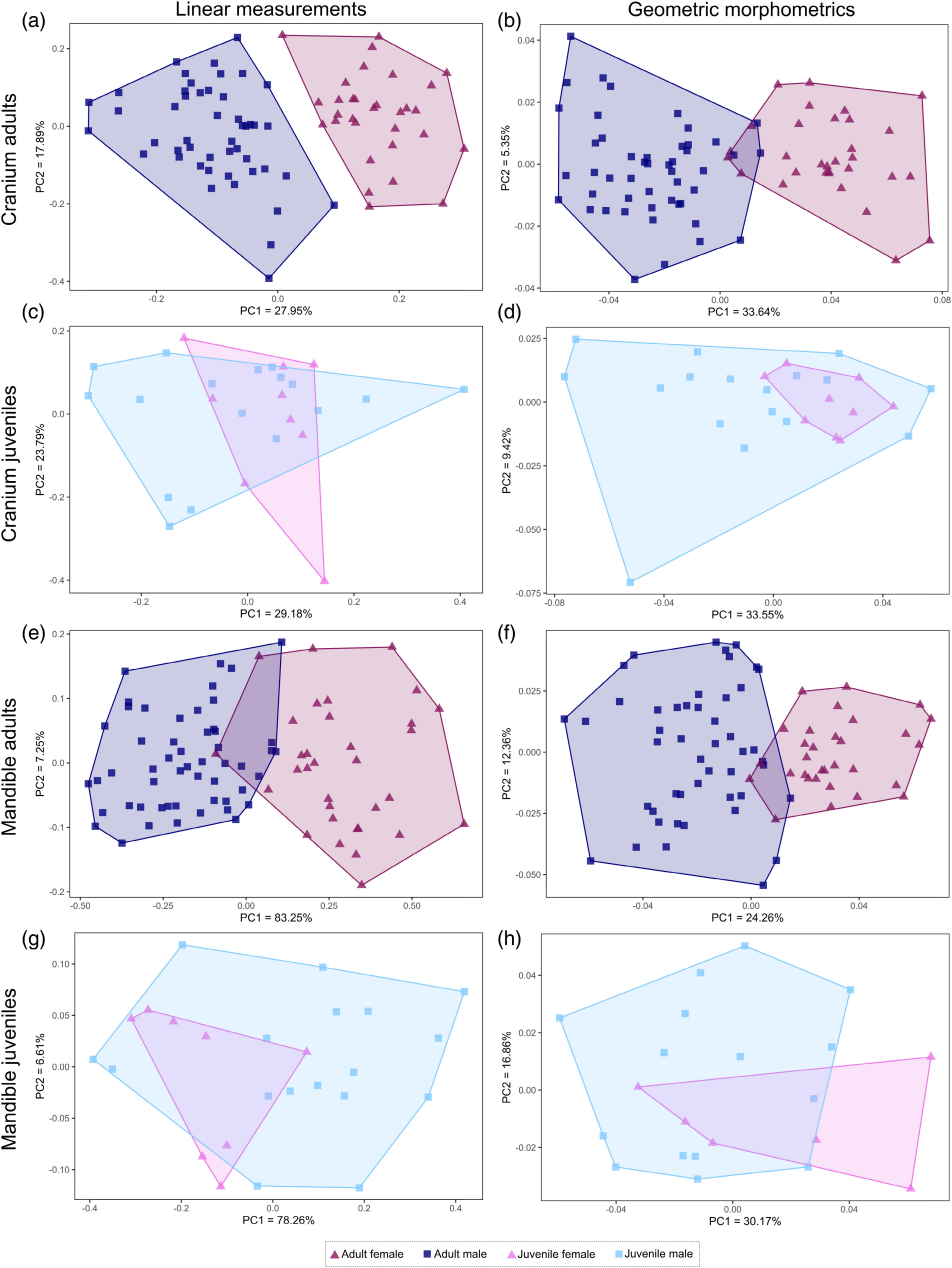
Sexual dimorphism for Atlantic walrus adults (a, b, e, f) and juveniles (c, d, g, h) using linear measurements (a, c, e, g) and geometric morphometrics (b, d, f, h) for crania (a, b, c, d) and mandibles (e, f, g, h). (a) sPC1 versus sPC2 for adult crania; (b) PC1 versus PC2 for adult crania; (c) sPC1 versus sPC2 for juvenile crania; (d) PC1 versus PC2 for juvenile crania; (e) sPC1 versus sPC2 for adult mandibles; (f) PC1 versus PC2 for adult mandibles; (g) sPC1 versus sPC2 for juvenile mandibles; (h) PC1 versus PC2 for juvenile mandibles.

Sexual dimorphism was assessed for adults and juveniles separately. Neonate walruses were not taken into consideration here due to the lack of females. Juvenile males and females were tested to see if sexual dimorphism is present and detectable from an early age. In both juveniles and adults, there is a strong allometric effect for crania and mandibles (Tables S8–S11). Adult males are significantly larger than adult females for both crania and mandibles (Tables S8 and S9) and show clear differences in shape (Figure 7; Tables S15–S18). In their crania, adult females have relatively longer and narrower crania, more slender rostra and frontals, more posteriorly positioned occipital ridges, lower nasal bridges, smaller mastoid processes, more curved palates, wider nasal apertures, and wider occipital condyle bases compared to adult males. The mandibles of adult females have more slender rami, a smaller symphysis, a lower mandible, and a wider angle between the mandibles compared to adult males. Juvenile males and females are similar in size and shape (Figure 7, Tables S10 and S11), while some juvenile males are larger than juvenile females for both crania and mandibles (Figures S19–S22).

#### 3.2.4 Juvenile males versus adult females

As shown in Figures 4 and 6, adult females and juvenile males are morphologically very similar and were therefore compared more in‐depth. The allometric effect was significant for the cranium and mandibles (Table S12 and S13). The crania of some juvenile males were larger than those of adult females (Figures S23 and S24), but this difference was not significant (Table S12). There is no difference in the size of mandibles (Figures S25 and S26, Table S13). The cranium and mandible shape differences between juvenile males and adult females are minimal. Adult females can have slightly slenderer orbitals and zygomatic arches, more protruding occipital condyles, more curved palates, and more pronounced glenoid fossa compared with juvenile males as detected on PC2 of a PCA on geometric morphometrics on the cranium (Figure S24).

### 3.3 Linear discriminant analysis

In order to assess the applicability of classifying walrus remains to specific ontogenetic groups based on morphological quantitative features, a LDA was applied. Table 9 provides the overall success rates, and Table S18 in Data S6 provides the rates for individual measurements. Geometric morphometrics provides similar or more successful identification rates compared to linear measurements when using the complete landmark set for crania and, in some cases, for mandibles. Crania provide better success rates compared to mandibles. The overall success rate to classify specimens to an ontogenetic group was rather low, and most individual measurements had a success rate of <70%. This low success rate is mostly due to the wrong classification between juvenile males, juvenile females, and adult females. Neonates and adult males are generally correctly identified, with occasional misidentifications as juvenile males. Classifying juvenile and adult females from each other is moderately possible using LDA. Classifying juvenile and adult males from each other is highly successful, especially on crania. The LDA was highly successful in distinguishing adult females and adult males, with overall and most individual measurements resulting in a >90% correct identification rate (Table S18). It is not possible to confidently distinguish juvenile females and males using linear measurements, as all LDA tests were less than 80% successful. Similarly, juvenile males and adult females are difficult to distinguish using linear measurements, with many individual measurements having a success rate lower than 70%. Geometric morphometrics provides a better success rate in this case, especially for crania.

**TABLE 9:** Mean success rate (%) of 100× bootstrapped linear discriminant analysis using a training and testing set with an 80:20 ratio of the natural history specimens for specific ontogenetic groups using all linear measurements and the complete landmarks set for geometric morphometrics.

	All groups	Adult female—adult male	Juvenile female—juvenile male	Adult female—juvenile female	Adult male—juvenile male	Adult female—juvenile male
Linear measurements						
Cranium	66.67	99.87	68.46	78.31	91.69	75.56
Mandible	63.16	95.33	57.71	77.51	89.62	60.68
Geometric morphometrics						
Cranium	74.57	99.76	77.98	85.70	93.98	89.11
Mandible	70.42	92.91	60.80	75.99	85.06	81.60

The pooled variance of the fixed landmarks of the cranium (Table S19 in Data S6) shows that for the general set, ontogenetic series, and sexual dimorphism in adults, the landmarks on the occipital (foramen, condyles, and ventral side; landmarks 7–9, 32–33, 40–41) provide the most information. In the PCAs, PC1 was often associated with changes in the posterior part of the cranium, but also other morphological changes are present, which are not detected via pooled variance. The nasal aperture (landmarks 1–4), the tooth implantation (landmarks 10–11), and the implantation of the zygomatic arch on the tooth socket (landmarks 37) provide the least information to distinguish groups. Remarkable is the low variation in the nasal aperture using geometric morphometrics, while the width and height of the nasal aperture are often among the highest loadings on PC1 using linear measurements. Pooled variance of the fixed landmarks of the mandible (Table S20 in Data S6) shows that for the general set, ontogenetic series, and sexual dimorphism in adults, the posterior half of the mandible (landmarks 4, 6–7, 12–17) provides the most information, which is also seen in the PCAs, due to changes in mandible thickness and width of the mandible and a change in the angle of the rami and the posterior condyles between the groups. The anterior part (landmarks 1–3, 5, 11) and the shape of the condylar processes (landmarks 8–9) provide the least information to distinguish groups.

Twenty‐nine specimens of unknown sex were also subjected to this classification using only the measurements and landmarks present. Ages were determined using suture fusion states. The sex attribution was confirmed via DNA and shows that morphometric classification is highly successful for adult specimens (Table 10). DNA sexing had a success rate of 89.7% (26 of 29 samples) with three samples receiving an “inconclusive” designation (Table 10, Table S23, Figure S30). Y:A and Y:X ratios of FOC3833 indicated its sex as male, but the X:A ratio placed the sample as female, resulting in the overall genomic sex being set to “inconclusive”. FOC4657 demonstrated an abnormally high X:A ratio and an absence of reads mapping to the Y chromosome, indicative of mapping bias due to low read numbers, thus rendering the assigned sex “inconclusive”. FOC4664 exhibited Y:A and Y:X ratios that were indicative of being male, yet its X:A ratio fell between the ranges associated with male or female classification. The remaining samples exhibited classic X:A, Y:A, and Y:X ratios characteristic of either being male or female. Of the 21 adult specimens that were successfully sexed using DNA analysis, 19 were correctly sexed using morphometric approaches. Geometric morphometrics and linear measurements agreed in all but two inconclusive cases (FOC4332 and FOC4496) on the sex attribution, with linear morphometrics and geometric morphometrics being confirmed by DNA sexing, respectively, for these specimens. The success rate in juveniles was remarkably lower, with only two specimens (out of five) being correctly identified using morphometrics. FOC4510 was incorrectly assigned sex via morphological approaches, and for FOC4597 and FOC4616, the crania and mandibles were classified to different sexes by geometric morphometrics and linear measurements. As these three specimens are juveniles and sexual dimorphism is not detectable in this ontogenetic group, this is not surprising. The probability scores of the linear discriminant analysis showed high probabilities for most specimens.

**TABLE 10:** Sex classification results for selected Atlantic walrus test specimens (*n* = 29) using linear measurements (LMs) and geometric morphometrics (GMs) compared with DNA sex attribution.

FOC specimen	Age	Element	LM	Probability score LM	GM	Probability score GM	DNA
3771	Adult	Mandible	Female	0.9999985	Female	0.9999994	Female
3828	Adult	Cranium	Male	1.0000000	Male	0.9999999	Male
3833	Adult	Cranium	Male	1.0000000	Male	1.0000000	Inconclusive
4332	Adult	Mandible	Male	0.9300563	Female	0.5035707	Male
4419	Juvenile	Mandible	Male	0.9825407	Male	1.0000000	Male
4489	Adult	Cranium	Male	0.9957703	Male	1.0000000	Male
		Mandible	Male	0.9979328	Male	0.9999977	
4496	Adult	Mandible	Female	0.9806515	Male	0.9999998	Male
4510	Juvenile	Cranium	Male	0.9134991	Male	0.9999858	Female
		Mandible	Male	0.8840263	Male	0.9922623	
4512	Juvenile	Cranium	Male	1.0000000	Male	0.9999998	Male
4538	Adult	Mandible	Male	0.9983632	Male	0.9999995	Male
4543	Adult	Mandible	Male	0.9998866	Male	0.9999996	Male
4587	Adult	Mandible	Male	0.9997981	Male	1.0000000	Male
4597	Juvenile	Cranium	Female	1.0000000	Female	1.0000000	Female
		Mandible	Male	0.8713275	Male	0.9398736	
4600	Adult	Mandible	Male	0.9999782	Male	0.9999999	Male
4605	Adult	Cranium	Male	1.0000000	Male	1.0000000	Male
4608	Adult	Mandible	Female	0.9999997	Female	0.9999408	Female
4616	Juvenile	Cranium	Female	1.0000000	Female	0.8783179	Female
		Mandible	Male	0.5827381	Male	0.9716738	
4617	Adult	Cranium	Female	1.0000000	Female	1.0000000	Female
4619	Adult	Cranium	Male	0.9507351	Male	1.0000000	Male
		Mandible	Male	0.9169162	Male	0.9996160	
4657	Adult	Cranium	Male	0.9998909	Male	0.6859423	Inconclusive
4663	Adult	Mandible	Male	0.9999995	Male	1.0000000	Male
4664	Adult	Mandible	Male	0.9999977	Male	0.9999997	Inconclusive
4668	Adult	Mandible	Female	0.9999809	Female	0.9997537	Female
4669	Adult	Cranium	Male	1.0000000	Male	1.0000000	Male
		Mandible	Male	0.9903931	NA	NA	
4819	Adult	Cranium	Female	1.0000000	Female	1.0000000	Female
		Mandible	Female	0.9999993	Female	1.0000000	
4836	Adult	Mandible	Female	0.9998311	Female	0.9999993	Female
4841	Adult	Mandible	Female	0.9986101	Female	1.0000000	Female
4845	Adult	Cranium	Male	0.9999975	Male	0.9352307	Male
5061	Adult	Cranium	Male	0.9996379	Male	1.0000000	Male
		Mandible	Male	1.0000000	Male	1.0000000	

## 4 DISCUSSION

Using suture fusion states and morphometric approaches, we here present new results that show that it is possible to determine the age and sex of walrus remains. While this was already known for mandibles (e.g., Boisville et al., 2022; Taylor et al., 2020; Wiig et al., 2007), this study provides an in‐depth analysis for crania as well. Age categories can be determined easily using specific suture fusion states on the cranium and mandible, but this is limited to specific age classes due to the timing of the fusion having some variation between individuals and adult females having fewer fused sutures at a given age compared to males. Age can also be determined for neonates and adult males using morphometric approaches due to size differences and neonates having a distinct morphology, but is limited due to overlap for juveniles and adult females. Sex determination is only confidently possible in adults using morphometrics, where sexual dimorphism is clear. Below the biological relevance and results of age and sex determination, the success of classifying specimens, and the wider impact of this morphometric approach are discussed in more detail.

### 4.1 Age

The age classes of Atlantic walruses are easily determined via suture fusion states and morphometrics. The sutures in their crania and mandibles become gradually more fused as they get older (also see Taylor et al., 2020 for walruses and e.g., Tedman, 2003; Brunner et al., 2004; Stewardson et al., 2011 for other pinnipeds). On present evidence, this process follows a pattern, with some sutures fusing earlier than others. As observed in other pinnipeds, the posterior part of the skull, including the basioccipital, fuses earlier than the anterior part, which includes the premaxilla (e.g., Stewardson et al., 2011; Tedman, 2003). It is therefore possible to classify walrus specimens to age classes. Unfused sutures appear only in very young walruses under 4 years old. With the basioccipital, it is possible to differentiate walruses younger than 8 from walruses older than 10 years old, and with the premaxilla, those above 13 years old. In males, all sutures are fully fused above age 15, which corresponds to the age of completed growth (Fay, 1982). For adult females, it is more difficult to differentiate between different ages, and suture fusion seems to be less final in adult females from Parry Bay, while all observed males have fully fused sutures at 15 years of age. This might also be related to sexual dimorphism (see below). However, this suture fusion pattern is based only on a few specimens from one locality. It is unclear whether the same pattern holds true for other walruses in the Atlantic Ocean or Pacific Ocean due to the absence of records on ages for most walrus specimens housed in museums. Furthermore, not all ages are represented in both sexes in this study. A gap between ages 4 and 7 made it difficult to assess the onset of suture fusion. No male was available between ages 8 and 13, which is when fusion progresses in many of the sutures. More specimens in general, and from other localities, are needed to further assess the applicability of this method to determine specific ages of walrus remains. For now, it is recommended to use this method for aging with some precaution and to not date to precise ages, but to age to groups as detailed in Table 5. These suture fusion states provide age data without the need for other analyses and thus are highly suitable for aging walrus remains to age classes as a standalone approach. Future studies can further uncover the classification potential of this approach by increasing the sample size, expanding the geographical range, and applying predictive age classification modeling.

Morphometrically, age groups differ from each other in size and shape (Figures 4 and 6). Neonates are generally distinct from the other ontogenetic groups, while a few show morphological similarities with older walruses. While a potential geographical effect cannot be excluded due to small sample sizes, it is more likely to be very fast shape and size changes as the animal ages. For the most distinct neonates, no precise age data are available, but based on the collection date and metadata, it is estimated that these are less than 1 year old. Those older than 1 year, yet still with unfused sutures, start resembling juveniles rapidly as they age. Juveniles, in their turn, gradually undergo changes as they mature. Adult males are easily distinguished from the other age groups, while no shape or size difference between adult females and juvenile females or juvenile males was found on crania and mandibles. This was also observed by Taylor et al. (2020) and Boisville et al. (2022) for mandibles. The only way to distinguish adult females from juveniles is the suture fusion state.

### 4.2 Sexual dimorphism

Sexual dimorphism is clearly present in adults, with males being significantly larger and having distinct shapes compared to adult females, which corroborates previous research studying shape and size in adults (e.g., Boisville et al., 2022; Fay, 1982; Taylor et al., 2020; Wiig et al., 2007). Adult females do not grow as large and their shape is less distinct from juveniles. Male walruses have an extended growth period, as was noted by Fay (1982), where males only become fully grown several years after females. As there is no indication of sexual dimorphism in juveniles and as adult males are both larger and shaped differently than females, this indicates sexual bimaturism in walruses. Sexual dimorphism in walruses might be linked to sexual selection or ecomorphology. This extended growth period could therefore provide a more impressive appearance and better protect males in fights between each other for dominance and mating access (Sjare & Stirling, 1996). The strengthening of the sutures and increased thickness and width of the cranium could be adaptations allowing males to fight with reduced risk of serious injuries. The distinct shape of the mandible, however, seems less related to this. It has been observed that during play fighting or mate competition, males lift their heads and point their chins towards each other, which has been interpreted as an optical signal to display the tusks (Miller & Kochnev, 2021). The width of the mandible in this position might thus also play a role in optical signaling prior to fights with other males. There is currently no evidence that modern male and female walruses have distinct feeding habits that would explain such clear differences in size and shape (e.g., Barrett et al., 2022 for Atlantic walrus, Sheffield & Grebmeier, 2009; Seymour et al., 2014; Clark et al., 2022; Koch et al., 2021 for Pacific walrus). Even though males seem to be more likely to predate on and consume birds and other pinnipeds, this behavior is not exclusive for males, and is more frequent in immature walruses (Giljov et al., 2017; Gjertz, 1990; Lovvorn et al., 2010; Lowry & Fay, 1984; Mallory et al., 2004; Seymour et al., 2014). Therefore, the distinct morphology and large size of adult males seem to mostly be related to behaviors related to reproduction.

Sexual dimorphism between male and female juveniles was not detected on crania and mandibles. Generally, sexual dimorphism is less apparent in juvenile and neonate mammals (e.g., Hart et al., 2007; Sanfelice & de Freitas, 2008; Xia & Millar, 1987). However, due to the small sample size of females, it is not possible to exclude the possibility that sexual dimorphism appears in older juveniles. In Pacific walrus, juvenile males will gradually mature and obtain the adult male appearance (Taylor et al., 2020). Fay (1982) reported that juvenile males have wider heads compared to juvenile females. This also seems to be true for the juvenile specimens from Parry Bay, which are the only ones for which age is known independently in this study. For the other specimens in this study, some juvenile males have higher values, while others have similar or even smaller values compared to the juvenile females. Using width alone is thus unsuitable to distinguish juveniles, which is also shown by the low success rates of the LDA on linear measurements (SI). As no female neonates were available, sexual dimorphism during the first years of life could not be assessed. A more detailed ontogenetic analysis with more juveniles of known and specific ages is needed to further consider potential sexual dimorphism in this age class. While no ecological differences between juvenile females and males are reported, between ages 2 and 10, males gradually start to leave their mothers and join male herds, while females stay in the female herds (Fay, 1982). Differential exploitation of such herds in the past could thus be assessed by sexing remains of juvenile walruses, which seems to be difficult using morphological features only.

When working with museum collections, the risk for bias in size, especially toward larger individuals, has to be taken into account. As shown in Figures S2–S4, there are no gaps between the juvenile and adult females and males for isosize and centroid size. Juvenile males also overlap slightly with adult males, as do juvenile females with adult females. While there might be an overabundance of larger individuals present in the studied collections, there is no evidence of absent sizes in the adult groups, which could have impacted the results.

### 4.3 Classification

Linear discriminant analysis provides a high success rate to distinguish adult males and females using both linear measurements and geometric morphometrics. The best result of identifying walrus remains to an ontogenetic group is obtained in combination with aging the specimen using the suture fusion states to differentiate the age classes, which then allows further classification between adult males and adult females. This is largely due to the lack of shape and size differences between adult females and all juveniles, which do not show any sexual dimorphism. Adult males and females can easily be distinguished due to differences in shape and size, resulting in a >99% success rate for complete crania and >92% for complete mandibles using linear measurements and geometric morphometrics. Boisville et al. (2022) reported similar success rates for mandibles. The high success rate to distinguish adult males and females using linear measurements is most likely attributable to the differences in size. Using geometric morphometrics, the size effect is removed without reducing the accuracy, showing there are sufficient shape differences between the sexes also. For juveniles, the success rate is much lower and the classification technique proposed here is not sufficient to distinguish males and females due to the lack of dimorphism in this age class, as was also noted by Taylor et al. (2020). The success of classification was also confirmed for the unknown test specimens using DNA sexing.

Fragmentation and abrasion are well‐known issues for morphometric analyses that can have severe negative effects on the success rate of the classification. In the classification test performed here, even fragmented specimens were correctly classified. Geometric morphometrics in general needs a minimum of three landmarks to work, and the success rate depends on which part of the specimen remains available and the additional landmarks available. In the case of linear measurements, it is in theory sufficient to use one measurement only to distinguish male and female adult walruses, due to the difference in size between the two sexes (see Table S18 in Data S6). Fragmentation should have a limited effect, but a good comparative set to show the variation for both sexes for that measurement is required. Using this approach, Barrett et al. (2020) were able to successfully classify rostra using a few measurements inside the tusk sockets, which is also confirmed to work by the present study (see Data S5; Figure S28, Table S15). Similarly, Wiig et al. (2007), Monchot et al. (2013), and Taylor et al. (2020) have shown the usefulness of mandibular measurements to identify males and females. For some measurements, however, such as those around the snout, the classification success rate is markedly lower (Table S18), which should be considered when working on fragmented specimens. The best linear measurements to allow classification are those with the highest loadings on either side of the bar plots in Figure 5 (Baur & Leuenberger, 2011): orbital width (OW), rostral width (RW), outer socket width (SWO), neck of occipital condyles (OC), and snout width (SnW) for crania, and mandible thickness (ManT), mandible width (MaW), and mandible length (ManL) for mandibles.

While a significant effect of and interaction between size and ontogenetic groups was mostly found for crania and mandibles for both linear measurements and geometric morphometrics, the low observed r^2^ values related to ontogenetic groups of the Procrustes ANOVAs indicate there is a lot of unexplained variance in the models, which can stem from natural shape variation within an ontogenetic group, measurement error, or even geographical variation. The morphometric data and classification test provided here only reflect the Atlantic walrus and do not include the Pacific subspecies. The mandibular sexual dimorphism for the Pacific walrus has been included in Boisville et al. (2022), who used mostly Pacific specimens for their study, and Taylor et al. (2020). It is generally accepted that the Pacific walrus is on average larger than the Atlantic walrus, but certain females from some areas in the Atlantic, such as those from Foxe Basin, might be larger than those from the Pacific population (Dyke et al., 1999; Garlich‐Miller and Stewart, 1998). This could indicate that size‐related sexual dimorphism might be more prominent in the Pacific subspecies. Although only minor osteological differences between the two subspecies are reported (Fay, 1982), ontogenetic differences and sexual dimorphism should also be assessed in the Pacific walrus, which includes the Laptev walrus (Lindqvist et al., 2009; Mills et al., 2024), to assess size and shape patterns in crania and mandibles. Furthermore, interpopulation differences should also be assessed in more detail, as there are indications that walruses from different areas in the Atlantic might differ in size and perhaps also shape (e.g., Knutsen & Born, 1994; McLeod et al., 2014). There were no indications of this in this study, but the sample sizes for different areas included were too small to assess in detail (also see Dierickx et al. in prep.).

## 5 IMPACT

Defining the age and sex of walrus remains is an important and typically essential foundation for inferring both how humans have utilized walruses in the past and walrus population compositions (Barrett et al., 2020; Desjardins, 2018; Dyke et al., 1999; Gotfredsen et al., 2018; Murray, 1992). By defining and comparing past exploitation strategies and their impacts on past populations of walrus, modern day conservation practices can also be assessed and improved. Fundamental age and sex information is central to almost all potential research entailing both the cultural and environmental aspects of historical ecology, especially insofar as the preferred targets of past and present hunting vary (e.g. Gotfredsen et al., 2018). Combined with traditional ecological knowledge, demographic information provides an important starting point for understanding long‐term changes in walrus distribution and exploitation (Born et al., 2017; Martinez‐Levasseur et al., 2021; Wiig et al., 2007).

Furthermore, morphometric and morphological classification and identification of sex and age groups can be used to classify specimens held in museum collections for which no metadata are available. The robustness of future studies on osteological specimens can be improved without the need to destructively sample in order to differentiate males and females using DNA. This new approach can also be applied in order to more accurately and specifically select samples for destructive sampling, reducing the impact on the material for studies such as stable isotope analysis and DNA. The interpretation of such analyses is also heavily dependent on insights into sex and age distributions due to behavioral, migratory, and dietary differences and human exploitation preferences (e.g., Fay, 1982; Wiig et al., 2007).

In order to facilitate future work on classifying age and sex groups of walrus remains, the Data S5 contains recommendations on how to use the data and code provided in this study (SI) for these purposes.

## 6 CONCLUSION

Linear measurements and geometric morphometrics allow the study of ontogeny and sexual dimorphism in the Atlantic walrus. Suture fusion states are strongly associated with age. There are clear size and shape differences between adults and neonates, with juveniles showing a gradual transition between these. Sexual dimorphism is clearly present in adults, while none was detected in juveniles and neonates. These clear morphological differences allow the identification of unknown material to age groups and sex based on suture fusion states and morphology. It is recommended to first assign a specimen to an age group and for adult walruses then to differentiate between males and females. This results in a high success rate, which was confirmed using DNA analysis. Better classification of osteological material will improve further studies on walruses to understand their ecomorphology, as well as to provide highly needed insight into their historical exploitation and impacts on their populations through time.

## AUTHOR CONTRIBUTIONS

**Katrien Dierickx:** Conceptualization; formal analysis; investigation; data curation; writing – original draft; writing – review and editing; visualization. **Oliver Kersten:** Formal analysis; visualization; writing – review and editing. **Youri van den Hurk:** Conceptualization; resources; writing – review and editing. **Brenna A. Frasier:** Writing – review and editing; resources. **Richard Sabin:** Writing – review and editing; resources. **Bastiaan Star:** Writing – review and editing; supervision. **James H. Barrett:** Conceptualization; resources; writing – review and editing; supervision; project administration; funding acquisition.

## CONFLICT OF INTEREST STATEMENT

The authors declare no conflict of interest in relation to this study.

## Supporting information


**Data S1:** Supporting information.

**Data S2:** SCRIPT_DIERICKXetal_ClassifyWalrus.

**Data S3:** RSCRIPT_DIERICKXetal_OntogenyWalrus.

**Data S4:** RSCRIPT_DIERICKXetal_Socket analysisWalrus.

**Data S5:** Additional results.

**Data S6:** Additional tables.

**Data S7:** Database—Table S22—database.

**Data S8:** Boxplots.

## Data Availability

All raw data can be found in the SI, Table S22 in Data S7. Additional data and results are available in Data S5, S6, S7, S8. The R scripts of the morphological analysis and classification analysis are provided in the SI. PTS files are available in the SI. 3D models for all specimens with rights‐holder permissions to share (258 of 260 models) can be found on Morphosource, project ID 000691735. Raw DNA sequencing data have been deposited in the European Nucleotide Archive (ENA) under the study accession number PRJEB85630.
