# Supplementary material for: Ontogenetic changes and sexual dimorphism in the cranium and mandible of the Atlantic walrus (Odobenus rosmarus rosmarus L.)
Source: Anat Rec (Hoboken). 2025 Sep 12;309(7):1875–903. doi: 10.1002/ar.70050 (PMC13251751; doi:10.1002/ar.70050)
Supplement: Supplementary file 5 — Data S5: Additional results. [file AR-309-1875-s008.pdf]

Supplementary Information 1

Title: Ontogenetic changes and sexual dimorphism in the cranium and mandible of the Atlantic walrus (*Odobenus rosmarus rosmarus* L.)

Authors: Katrien Dierickx[1], Oliver Kersten[2], Youri van den Hurk[1,3], Brenna A. Frasier[4], Richard Sabin[5], Bastiaan Star[2], James H. Barrett[1]

Affiliations

[1]Department of Archaeology and Cultural History, NTNU University Museum, Trondheim, Norway
[2]Centre for Ecological and Evolutionary Synthesis, Department of Biosciences, University of Oslo, Oslo, Norway
[3]Hokkaido University Museum, Hokkaido University, Sapporo, Japan
[4]Nova Scotia Museum, Halifax, Nova Scotia, Canada
[5]Vertebrates Division, Natural History Museum, London, UK

Corresponding author: Katrien Dierickx; katrien.dierickx.icht@gmail.com

Consistency test

Five, mostly complete, male adult specimens from a larger dataset were randomly selected for both mandible dataset and cranium dataset to test the intraperson consistency in placing the landmarks. Each specimen was landmarked using the templates seven times in consecutive weeks. The resulting .pts files were loaded into Rstudio and a principal component analysis was performed as described in the methods to verify the differences between iterative placing of landmarks of an individual specimen were minor compared to the differences between individual specimens. The individuals show consistent landmarking with no overlap, and each take up only a small part of the morphospace. Only specimen 4787 shows some less consistent landmarking of the mandible on one occasion (third round).

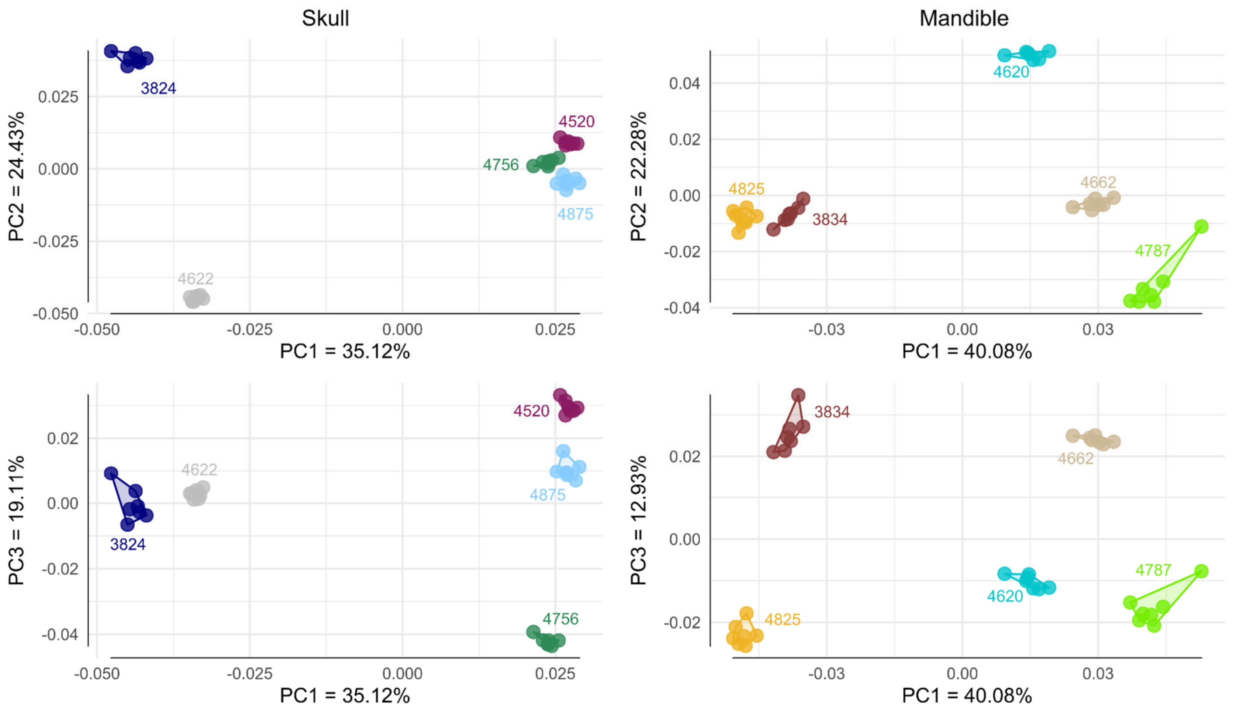

Figure S1. Principal component analysis (PCA) scatter plots on GMM data of adult male crania (left) and mandibles (right) of PC 1 versus PC 2 (top) and PC3 (bottom) coloured by individual specimen, labeled by its unique identifier.

Age estimation suture fusion

Table S1. Results of the Parry Bay subset with age data used for cranium suture fusion.

ID	Sex	Age (years)	Coronal	Nasal	Premaxilla	Basi-occipital	Mandible
4506	male	1.2	unfused	unfused	unfused	unfused	unfused
4507	male	3.2	unfused	unfused	unfused	unfused	NA
4493	female	7.3	part. fused	part. fused	part. fused	part. fused	part. fused
4508	male	8.1	part. fused	part. fused	part. fused		mostly fused
4504	female	8.2	part. fused	part. fused	part. fused	part. fused	NA
4495	male	8.3	part. fused	part. fused	part. fused	part. fused	mostly fused
4492	female	11.2	fused	mostly fused	mostly fused	fused	mostly fused
4494	female	12.3	part. fused	fused	mostly fused	fused	mostly fused
4518	male	13.1	mostly fused	mostly fused	fused	fused	mostly fused
4520	male	13.2	fused	mostly fused	fused	fused	fused
4515	male	15.2	fused	fused	fused	fused	fused
4517	male	17.1	fused	fused	fused	fused	fused
4491	male	18.2	fused	fused	fused	fused	fused
4490	male	20.2	fused	fused	fused	fused	fused
4503	female	22.2	mostly fused	fused	fused	fused	mostly fused
4509	male	23.2	fused	fused	fused	fused	fused
4505	female	26+	fused	fused	fused	fused	mostly fused
4514	female	28+	mostly fused	fused	fused	fused	fused

Descriptive statistics

Table S2. Descriptive statistics of cranium measurements per ontogenetic group. All measurements are expressed in mm. Measurement abbreviations are explained in Table 2. n = number of individuals; sd = standard deviation; min = minimum value; max = maximum value; GM = geometric mean.

Measurement	Ontogeny group	n	mean	sd	median	min	max	variance
**RW**	mal_neo	2	71.4	3.04	71.4	69.25	73.55	9.24
	unk_neo	7	68.82	11.01	69.63	56.32	84.65	121.19
	mal_juv	15	151.15	26.55	157.62	108.71	195.78	704.81
	fem_juv	8	137.13	8.10	136.27	128.67	154.50	65.66
	mal_adu	47	194.85	15.49	193.25	158.86	238.15	239.92
	fem_adu	29	144.70	7.18	145.48	130.1	159.42	51.57
**OW**	mal_neo	4	98.77	25.45	91.00	78.74	134.36	647.70
	unk_neo	7	77.17	11.89	76.5	63.33	92.32	141.35
	mal_juv	16	150.73	20.57	154.11	122.99	193.00	422.95
	fem_juv	9	131.30	7.54	134.98	118.99	141.04	56.81
	mal_adu	45	187.66	13.71	188.02	160.77	222.66	188.01
	fem_adu	28	136.08	10.48	134.72	119.02	159.45	109.87
**IW**	mal_neo	4	56.185	10.01	54.10	46.94	69.61	100.14
	unk_neo	7	51.01	7.92	48.31	44.25	64.29	62.77
	mal_juv	17	70.64	8.78	71.07	51.61	87.83	77.16
	fem_juv	9	62.31	6.44	64.83	51.1	70.7	41.51
	mal_adu	47	76.69	8.19	76.87	58.39	94.03	67.03
	fem_adu	29	62.11	5.49	62.18	44.59	72.23	30.10
**ZW**	mal_neo	3	150.27	43.42	130.06	120.64	200.11	1885.20
	unk_neo	7	119.29	17.58	118.06	98.81	139.99	309.11
	mal_juv	14	209.6	23.00	213.15	172.44	247.94	529.11
	fem_juv	8	195.32	8.23	191.91	183.83	207.67	67.67
	mal_adu	43	239.95	11.41	239.53	214.88	259.64	130.28
	fem_adu	26	202.41	7.23	201.57	191.25	221.18	52.28
**nOC**	mal_neo	3	178.9	27.58	172.2	155.29	209.21	760.51
	unk_neo	6	156.06	19.12	154.17	135.2	177.4	365.41
	mal_juv	17	223.47	11.17	221.85	204.45	240.13	124.80
	fem_juv	9	215.54	11.17	211.19	204.64	242.53	124.74

	mal_adu	45	242.01	12.84	242.27	219.11	272.00	164.88
	fem_adu	29	218.53	10.29	219.08	199.77	241.00	105.86
**NL**	mal_neo	3	43.98	24.50	31.58	28.16	72.2	600.20
	unk_neo	6	32.73	9.06	32.93	19.11	43.35	82.03
	mal_juv	17	66.91	8.56	66.28	55.55	85.31	73.21
	fem_juv	9	61.57	10.70	65.53	39.79	74.95	114.48
	mal_adu	47	77.36	9.57	77.48	53.21	100.25	91.56
	fem_adu	29	63.99	8.29	64.62	47.08	83.11	68.70
**ONL**	mal_neo	219	83	64.20	196.21	170.79	292.5	4121.88
	unk_neo	5	170.75	26.30	158.88	145.92	205.53	691.53
	mal_juv	12	302.85	21.87	308.47	272.83	337.63	478.24
	fem_juv	7	283.23	8.16	283.78	270.2	298.06	66.67
	mal_adu	38	332.38	14.16	332.78	307.64	371.69	200.40
	fem_adu	25	293.02	11.69	293.36	269.35	315.46	136.59
**CBL**	mal_neo	4	227.90	66.99	209.54	170.77	321.74	4487.13
	unk_neo	6	166.14	23.63	160.88	141.98	201.33	558.39
	mal_juv	12	334.37	34.46	342.25	289.01	387.52	1187.74
	fem_juv	7	312.13	8.40	314.63	298.23	321.53	70.64
	mal_adu	35	371.60	14.20	370.57	349.36	406.76	201.62
	fem_adu	25	324.60	12.43	324.03	298.81	349.85	154.41
**PH**	mal_neo	4	100.37	29.20	101.29	64.04	134.87	852.72
	unk_neo	6	89.22	12.96	83.24	77.49	111.25	168.00
	mal_juv	13	134.55	12.39	135.72	112.62	154.02	153.49
	fem_juv	9	126.72	3.88	127.27	119.19	133.59	15.08
	mal_adu	38	149.99	6.93	149.20	134.88	162.17	47.96
	fem_adu	25	130.85	5.07	130.91	120.61	141.58	25.71
**uPW**	mal_neo	4	169.29	42.50	159.13	131.66	227.25	1806.21
	unk_neo	7	136.28	19.64	130.87	112.28	160.88	385.89
	mal_juv	17	240.35	24.27	240.06	208.89	290.83	589.15
	fem_juv	9	219.12	6.13	217.81	208.68	227.87	37.61
	mal_adu	41	281.62	16.11	282.35	245.67	310.83	259.42
	fem_adu	26	230.27	10.19	229.46	212.82	249.85	103.91
**lPW**	mal_neo	4	158.47	47.76	145.22	117.25	226.2	2280.80

	unk_neo	7	121.88	18-54	118.32	99.87	145.79	343.72
	mal_juv	16	238.16	30.21	240.97	194.88	292.42	912.79
	fem_juv	9	223.90	11.73	225.89	204.11	238.09	137.70
	mal_adu	35	292.93	17.97	292.02	245.63	324.39	323.01
	fem_adu	24	237.74	10.70	238.52	221.35	266.05	114.54
**SnH**	mal_neo	3	39.51	14.09	33.59	29.35	55.6	198.58
	unk_neo	5	31.31	4.44	32.65	26.58	35-48	19.71
	mal_juv	17	48.67	5.08	49.18	40.85	56.79	25.83
	fem_juv	9	45.91	4.04	45.93	39.57	52.25	16.36
	mal_adu	48	50.35	5.38	50.06	39.84	64.15	28.99
	fem_adu	31	43.76	4.33	43.29	35.49	51.82	18.76
**SnW**	mal_neo	3	27.67	7.76	224.07	22.36	36.58	60.27
	unk_neo	5	22.79	5.43	23.5	16.73	28.95	29.50
	mal_juv	15	35.29	3.81	35.58	28.14	43.39	14.51
	fem_juv	9	34.70	1.98	33.81	32.34	37.82	3.91
	mal_adu	45	38.41	4.38	38.57	28.34	49.61	19.17
	fem_adu	31	35.02	3.02	35.07	29.23	41.13	9.12
**PMH**	mal_neo	4	31.17	11.81	26.88	22.33	48.6	139.75
	unk_neo	7	20.68	3.01	19.93	16.57	24.56	9.05
	mal_juv	17	47.96	9.88	47.17	31.47	64.06	97.65
	fem_juv	9	43.85	3.57	44.1	38.48	48.29	12.72
	mal_adu	47	55.10	5.98	54.26	42.08	67.86	35.73
	fem_adu	31	47.14	4.12	47.15	39.25	57.3	16.93
**SWo**	mal_neo	2	67.58	0.23	67.58	67.41	67.74	0.05
	unk_neo	7	65.57	9.72	68.35	53.63	77.96	94.52
	mal_juv	12	141.33	19.58	146.37	105.67	172.75	383.32
	fem_juv	8	134.37	6.88	133.84	126.33	146.62	47.32
	mal_adu	34	182.51	14.03	181.09	144.54	214.69	196.96
	fem_adu	22	141.30	5.51	141.68	130.75	151.89	31.49
**OC**	mal_neo	4	85.25	19.87	82.34	64.4	111.9	395.00
	unk_neo	6	66.82	10.90	66.22	55.04	81.12	118.80
	mal_juv	12	109.62	8.03	109.65	97.17	126.87	64.52
	fem_juv	7	104.38	3.37	104.25	99.68	108.26	11.35

	mal_adu	39	133.51	6.04	112.58	100.36	127.73	36.43
	fem_adu	25	105.00	5.14	104.73	94.52	116.00	26.38
**ML**	mal_neo	2	126.05	64.78	126.05	80.24	171.86	4197.11
	unk_neo	7	78.45	12.25	77.34	63.87	95.34	150.05
	mal_juv	17	185.88	22.10	189.86	148.04	227.14	488.58
	fem_juv	9	168.55	7.40	167.28	158.92	177.16	54.83
	mal_adu	38	213.88	12.62	211.83	197.23	245.09	159.28
	fem_adu	25	183.15	9.15	181.24	165.62	204.42	83.65
**MW**	mal_neo	3	59.32	20.05	50.64	45.07	82.25	402.09
	unk_neo	6	42-54	7.17	40.57	34.32	52.24	51.47
	mal_juv	16	73.82	8.59	72.81	62.22	86.54	73.83
	fem_juv	8	71.54	3.39	70.72	67.33	77.67	11.47
	mal_adu	40	78.51	6.34	79.33	64.84	90.22	40.25
	fem_adu	25	68.92	4.12	68.62	62.93	81.57	17.00
**GM**	mal_neo	4	7.05	0.83	6.9	6.24	8.1	0.70
	unk_neo	7	6.33	0.37	6.26	5.8	6.79	0.14
	mal_juv	17	8.03	0.37	7.97	7.35	8.82	0.14
	fem_juv	9	7.83	0.25	7.87	7.36	8.1	0.06
	mal_adu	48	8.40	0.54	8.55	6.28	9.38	0.29
	fem_adu	31	7.79	0.46	7.9	6.32	8.36	0.21

Table S3. Descriptive statistics of mandibular measurements per ontogenetic group. All measurements are expressed in mm. Measurement abbreviations are explained in Table 2. n = number of individuals; sd = standard deviation; min = minimum value; max = maximum value; GM = geometric mean.

Measurement	Ontogeny group	n	mean	sd	median	min	max	variance
**ManL L**	mal_neo	2	111.05	9.48	111.05	104.34	117.75	89.78
	unk_neo	7	105.71	15.81	101.11	87.41	127.35	249.81
	mal_juv	16	233.23	25.23	242.49	187.89	278.59	636.42
	fem_juv	6	214.80	11.57	209.80	204.68	232.52	133.77
	mal_adu	47	265.62	14.63	263.55	234.72	301.16	214.14
	fem_adu	32	227.93	12.56	226.95	204.08	257.89	157.67
**ManL R**	mal_neo	3	119.83	16.70	119.46	103.32	136.72	278.99
	unk_neo	5	109.73	15.29	114.98	89.4	125.00	233.78
	mal_juv	15	231.44	24.86	237.95	189.24	280.74	618.28
	fem_juv	6	215.56	11.63	209.61	205.81	233.89	135.28
	mal_adu	46	265.83	14.81	264.20	234.52	298.74	219.39
	fem_adu	32	231.18	21.03	227.35	203.70	323.13	442.47
**ManH L**	mal_neo	2	39.76	4.50	39.76	36.58	42.94	20.22
	unk_neo	7	37.92	5.95	36.99	30.16	45.73	35.36
	mal_juv	16	78.76	8.43	79.03	60.49	90.01	71.13
	fem_juv	7	73.64	5.26	73.24	64.62	81.38	38.70
	mal_adu	49	92.42	6.04	92.04	75.40	105.00	36.51
	fem_adu	32	77.56	5.75	77.27	66.68	92.39	33.07
**ManH R**	mal_neo	3	43.74	7.55	43.13	36.51	51.58	57.06
	unk_neo	7	37.74	6.04	36.14	29.78	45.05	36.47
	mal_juv	16	78.48	8.43	78.46	61.18	92.55	70.99
	fem_juv	7	72.88	4.80	73.01	65.42	78.83	23.05
	mal_adu	49	92.56	6.19	93.28	78.12	107.45	38.32
	fem_adu	32	77.77	5.23	76.53	68.30	91.67	27.31
**MD L**	mal_neo	2	25.765	1.85	25.77	24.46	27.07	3.41
	unk_neo	7	23.95	3.41	22.85	20.5	28.62	11.62
	mal_juv	17	52.63	5.90	53.61	39.14	61.01	34.83
	fem_juv	8	47.32	3.81	47.02	42.08	52.15	14.54
	mal_adu	50	63.31	6.09	62.76	52.63	78.86	37.06

	fem_adu	33	48.69	4.01	48.34	43.26	56.57	16.06
**MD R**	mal_neo	3	29.21	4.81	27.44	25.53	34.65	23.13
	unk_neo	7	23.57	3.37	22.2	20.05	28.22	11.38
	mal_juv	17	52.06	6.01	52.23	39.86	63.56	36.17
	fem_juv	7	47.72	3.89	48.8	43.18	53.34	15.13
	mal_adu	48	63.06	5.80	62.45	52.77	77.97	33.68
	fem_adu	33	48.71	4.13	48.23	41.70	57.25	17.05
**MaW**	mal_neo	2	106.22	10.31	106.22	98.93	113.51	106.29
	unk_neo	6	106.43	17.76	106.10	83.96	128.09	315.56
	mal_juv	16	193.05	17.41	197.61	163.76	225.03	306.52
	fem_juv	7	186.55	9.14	189.7	176.16	199.41	83.46
	mal_adu	51	223.24	12.33	223.66	200.27	243.59	153.05
	fem_adu	32	192.48	8.86	192.07	177.67	210.56	78.57
**MT L**	mal_neo	3	9.08	0.68	8.93	8.48	9.82	0.47
	unk_neo	7	8.66	1.36	8.54	6.93	10.53	1.84
	mal_juv	17	18.92	4.65	19.76	10.42	27.14	21.60
	fem_juv	8	14.70	2.02	14.02	12.79	19.26	4.10
	mal_adu	51	29.61	4.61	28.76	20.16	41.04	21.25
	fem_adu	33	17.07	2.66	17.08	11.92	26.17	7.09
**MT R**	mal_neo	NA	NA	NA	NA	NA	NA	NA
	unk_neo	6	8.54	1.14	8.6	6.91	10.38	1.29
	mal_juv	16	19.48	4.99	19.94	10.26	28.73	24.93
	fem_juv	8	14.51	1.45	14.14	13.09	17.51	2.11
	mal_adu	52	29.68	4.58	28.96	21.38	39.82	20.97
	fem_adu	33	17.21	2.55	17.36	11.91	25.74	6.52
**PrW L**	mal_neo	3	27.76	4.78	28.22	22.76	32.29	22.87
	unk_neo	6	24.21	3.62	23.78	19.87	28.66	13.12
	mal_juv	16	48.40	5.80	49.18	38.05	60.73	33.60
	fem_juv	6	44.48	3.73	44.76	40.51	50.73	13.94
	mal_adu	45	57.98	4.29	57.9	50.10	66.21	18.39
	fem_adu	30	48.26	3.59	48.18	40.02	55.02	12.91
**PrW R**	mal_neo	2	25.10	2.65	25.10	23.22	26.97	7.03
	unk_neo	5	23.34	4.62	20.5	19.29	28.48	21.34

	mal_juv	15	47.72	5.59	48.12	38.05	56.32	31.25
	fem_juv	6	43.88	2.58	43.67	40.82	47.33	6.64
	mal_adu	41	57.86	4.37	57.64	49.99	66.82	19.06
	fem_adu	30	47.24	3.37	47.09	39.28	54.23	11.36
**GM**	mal_neo	3	4.88	0.13	4.84	4.77	5.03	0.02
	unk_neo	7	4.49	0.48	4.32	3.73	5.11	0.23
	mal_juv	17	6.20	0.45	6.35	5.30	6.94	0.20
	fem_juv	8	5.65	0.24	5.86	4.08	6.19	0.45
	mal_adu	53	6.8	0.41	6.83	5.49	8.12	0.17
	fem_adu	33	6.09	0.40	6.11	4.06	6.64	0.16

Additional figures general ontogeny

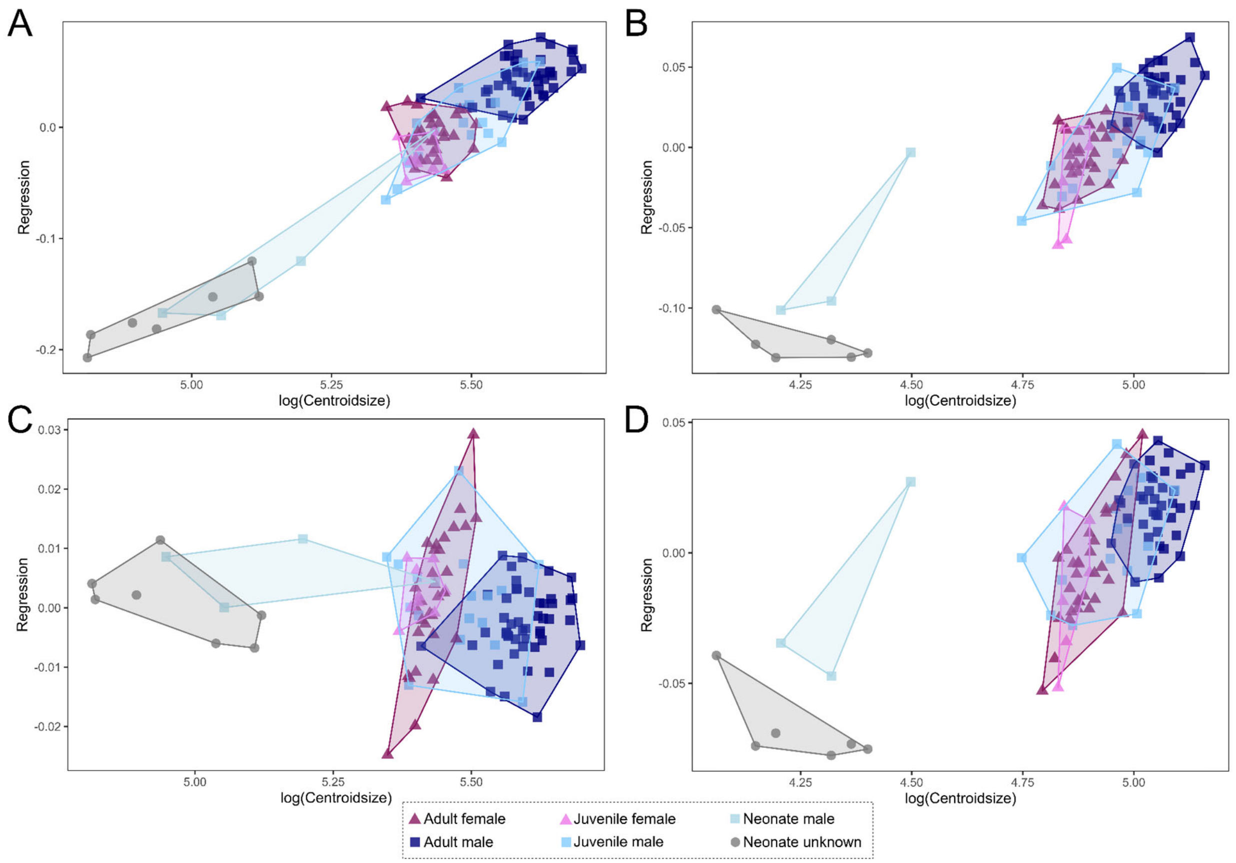

Figure S2. Regression scores from Procrustes ANOVA against log-transformed centroid size using geometric morphometrics. A. Shape~log(Centroid size) for crania; B. Shape~log(Centroid size) for mandibles; C. Shape~log(Centroid size)*Ontogenetic groups for crania; D. Shape~log(Centroid size)*Ontogenetic groups for mandibles.

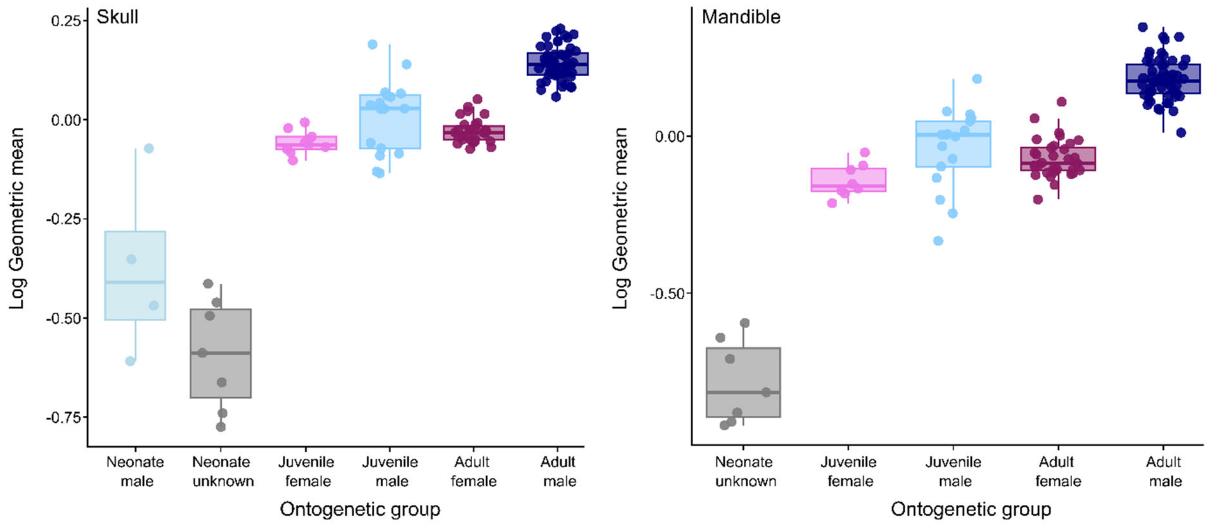

Figure S3. Boxplots showing the isosize for crania (left) and mandibles (right) per ontogenetic group.

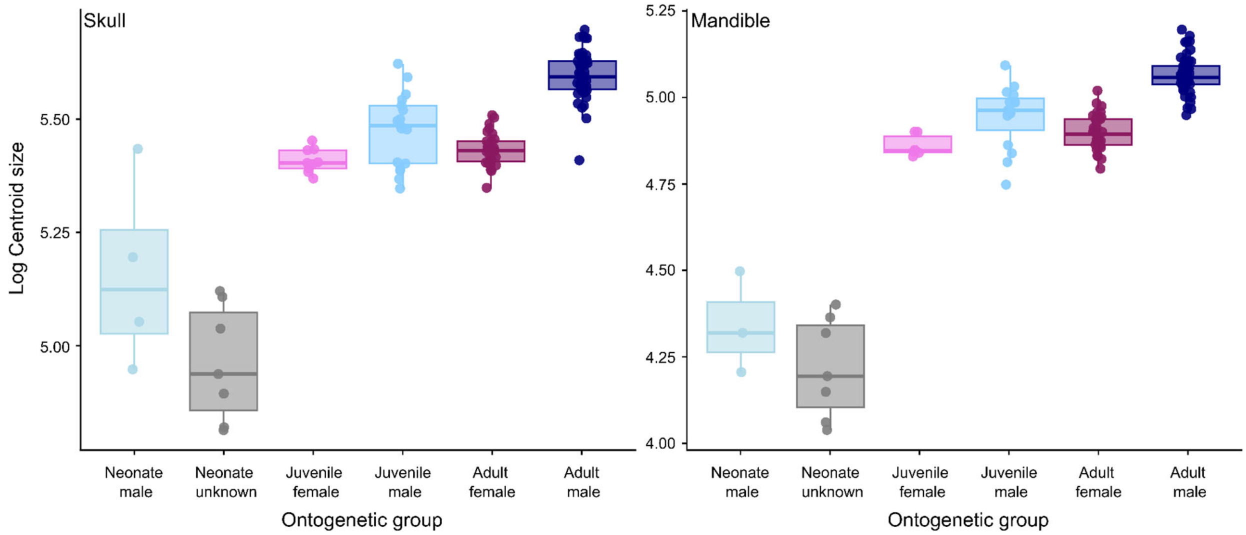

Figure S4. Boxplots showing the logarithmic of the centroid size for crania (left) and mandibles (right) per ontogenetic group.

Parry Bay age and ontogeny

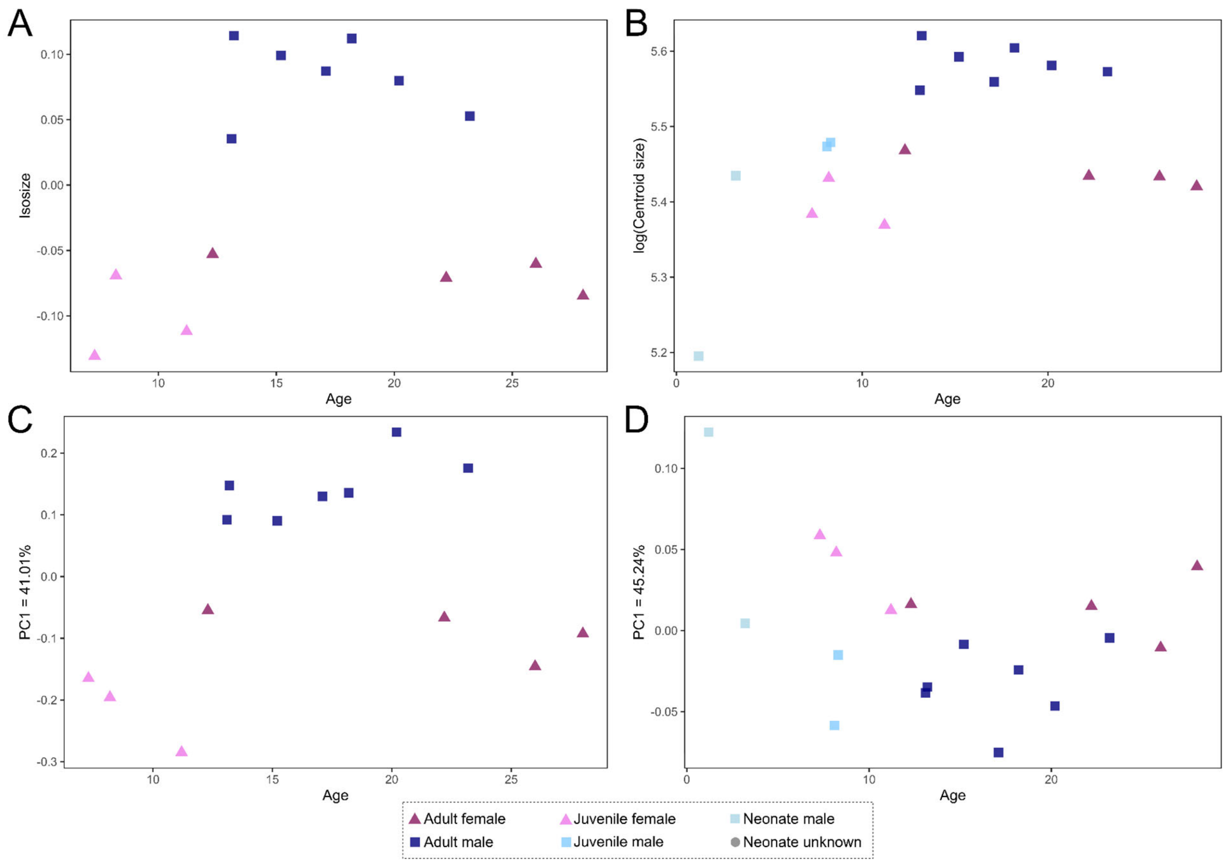

Figure S5. Size and shape comparison on crania of Parry Bay specimens per ontogenetic group compared with ages. A. Isosize versus age; B. log-transformed Centroid size versus age; C. sPC1 versus age; D. PC1 versus age.

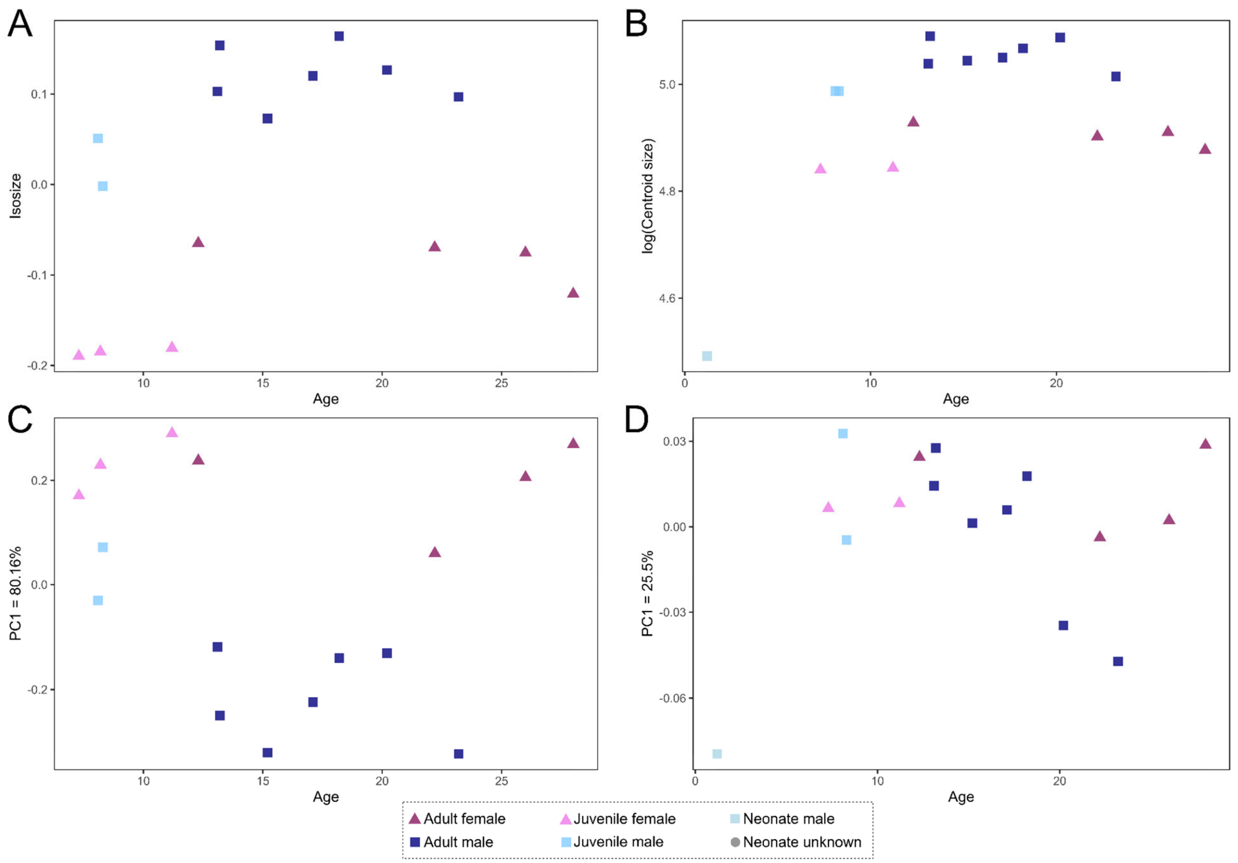

Figure S6. Size and shape comparison on mandibles of Parry Bay specimens per ontogenetic group compared with ages. A. Isosize versus age; B. log-transformed Centroid size versus age; C. sPC1 versus age; D. PC1 versus age.

Ontogeny series females

Cranium

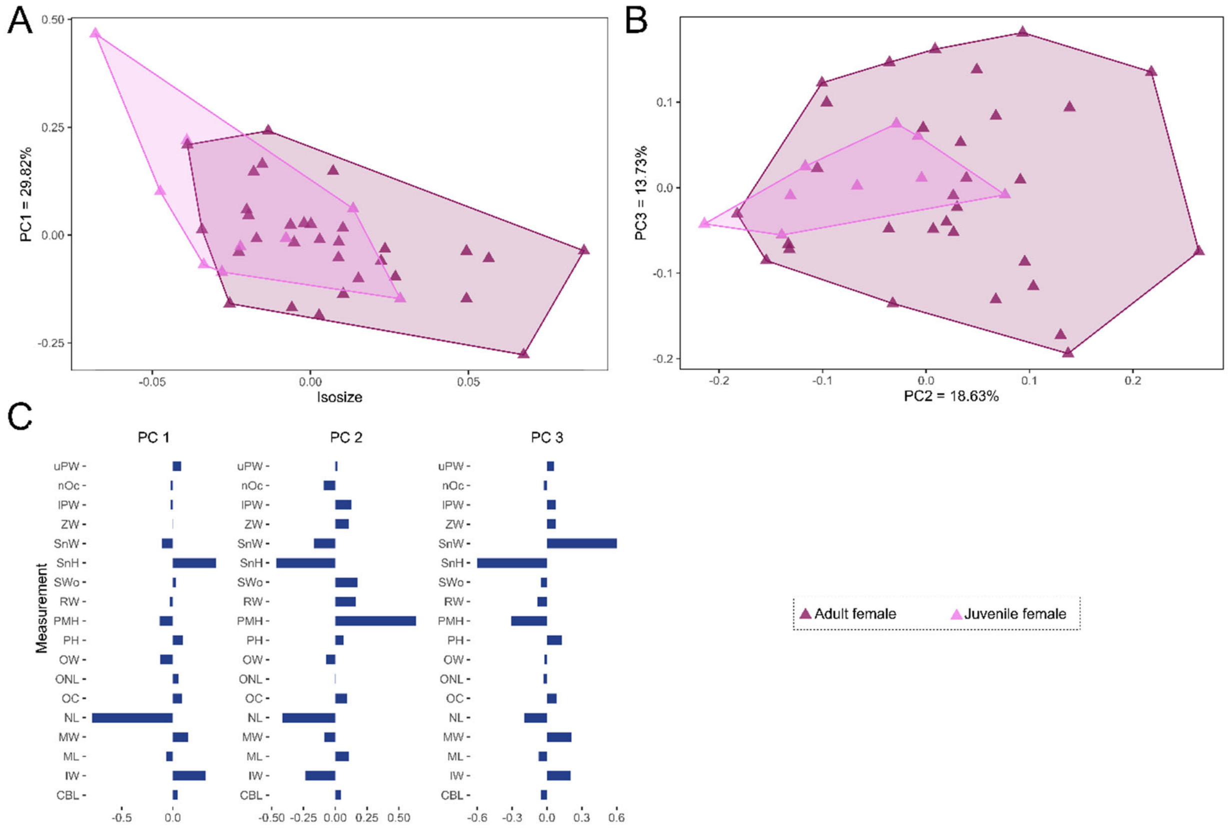

Figure S7. Ontogenetic series of females for crania. A. sPC1 versus Isosize; B. sPC2 versus sPC3; C. PCA loadings.

There is overlap between juvenile and adult females on sPC1 (29.82%), which is significantly correlated with size, and complete overlap on sPC2 (18.63%) and sPC3 (13.73%) for crania (Figure S7), which are not significantly correlated with size (Table S4). The most important loadings on sPC1 are NL, SnH, IW, MW, and PMH. The most important loadings on sPC2 are PMH, SnH, NL, IW, and SnW. The most important loadings on sPC3 are SnW, SnH, PMH, MW, and IW.

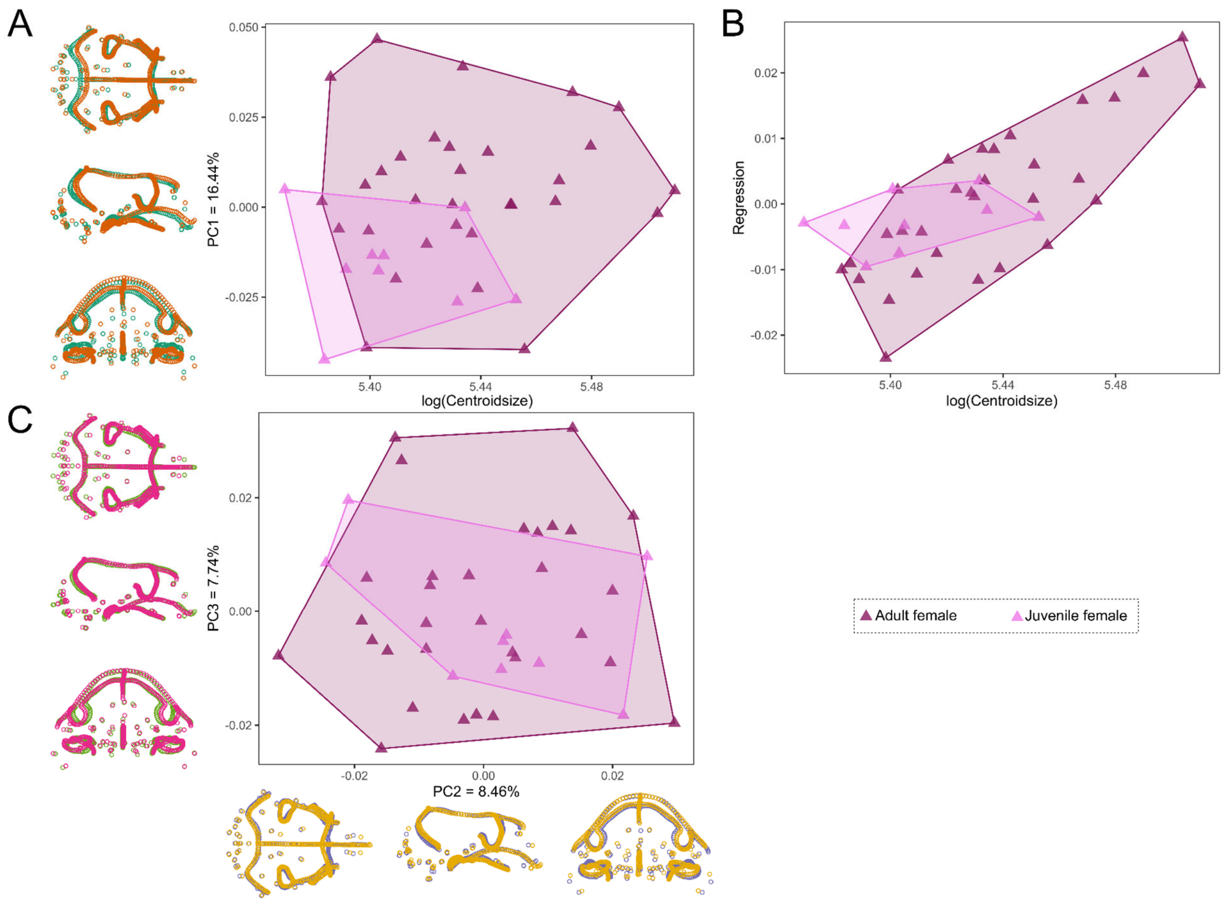

Figure S8. Ontogenetic series of females for crania. A. PC1 versus log-transformed Centroid size; B. Regression of Procrustes ANOVA versus log-transformed Centroid size per ontogenetic group; C. PC2 versus PC3.

There is strong overlap between juvenile and adult females on the three PC axes for crania (Figure S8), which are not significantly correlated with size (Table S4). PC1 is associated with shape changes in the occipital ridge and palate. PC2 is slightly associated with shape changes in the nasal ridge, the angle of the glenoid fossa and the orbital width. PC3 is slightly associated with shape changes in the orbital width and length of the occipital condyles.

Table S4. Output from correlation test, pairwise comparison, MANOVA, and regression models of crania for female ontogenetic series.

**Linear**							
	**Factor**	**p**	**cof**	**F**	**Pillai**	**df**	**den df**
**Shape ~Log Centroid size**		0.0001441		8.962	0.42754	1	36
**Shape ~ Ontogenetic group**		0.06717		2.5992	0.17804	1	36
**Shape ~Log Centroid size * Ontogenetic group**	Size	<0.001		9.6705	0.46042	1	34
	Group	0.007086		4.7591	0.29574	1	34
	Size:group	0.304878		1.2561	0.09978	1	34
**PC 1 ~ CS**		0.00127	-0.4917448				
**PC 2 ~ CS**		0.1489	-0.2324578				
**PC 3 ~ CS**		0.5218	-0.1043152				
**Pairwise**		0.016					

**GMM**							
	**Factor**	**p**	**cof**	**F**	**Z**	**df**	**r2**
**Shape ~Log Centroid size**		0.26		1.1134	0.74776	1	0.02847
**Shape ~ Ontogenetic group**		0.02		2.1092	2.5361	1	0.05259
**Shape ~Log Centroid size * Ontogenetic group**	Size	0.25		1.1291	0.79427	1	0.02847
	Group	0.02		1.8998	2.55177	1	0.04789
	Size:group	0.95		0.6370	-1.55675	1	0.01606
**PC 1 ~ CS**		0.3036	0.1667917				
**PC 2 ~ CS**		0.7267	-0.05703565				
**PC 3 ~ CS**		0.2656	-0.1803002				
**Pairwise**		0.064					

Mandible

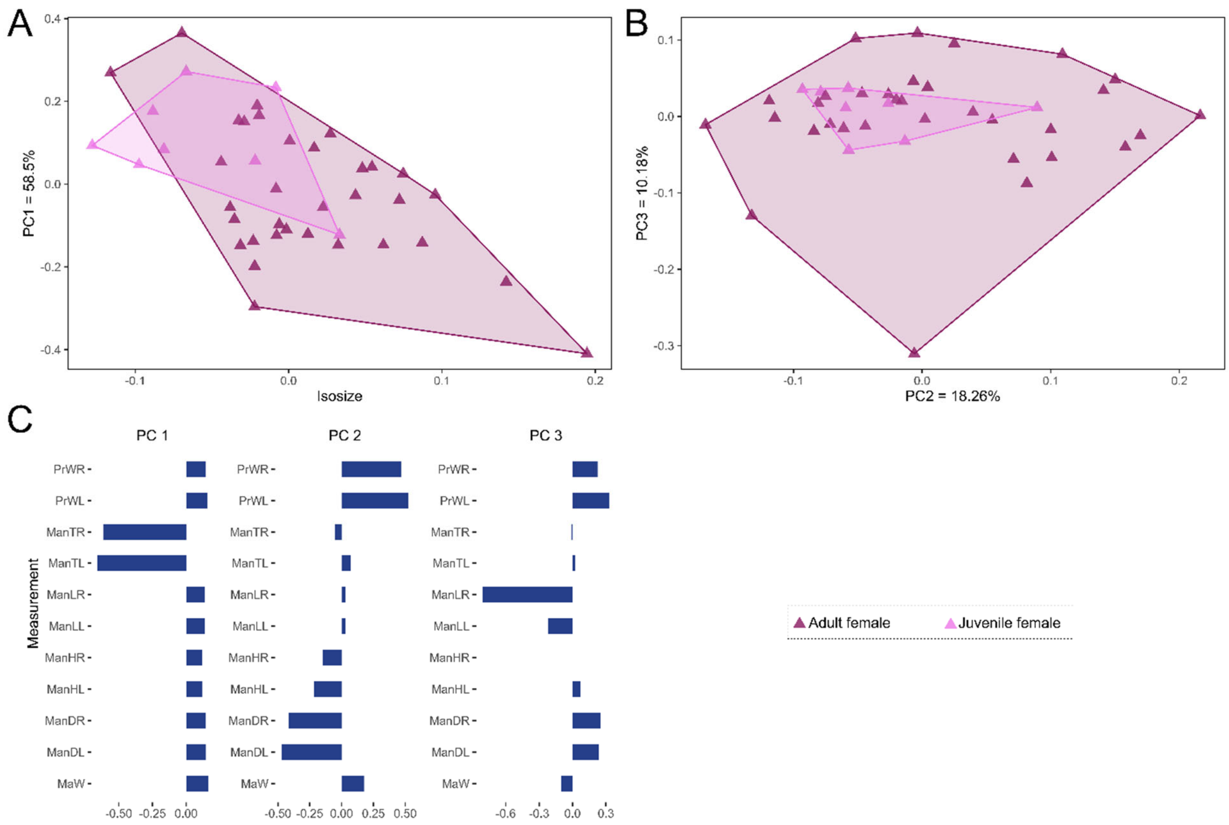

Figure S9. Ontogenetic series of females for mandibles. A. sPC1 versus Isosize; B. sPC2 versus sPC3; C. PCA loadings.

There is overlap between juvenile and adult females on sPC1 (58.50%), which is significantly correlated with size, and complete overlap on sPC2 (18.26%) and sPC3 (10.18%) for mandibles (Figure S9), which are not significantly correlated with size (Table S5). The most important loadings on sPC 1 are ManT, MaW, and PrW. The most important loadings on sPC 2 are PrW, ManD, and manH. The most important loadings on sPC3 are ManL, PrW, and ManD.

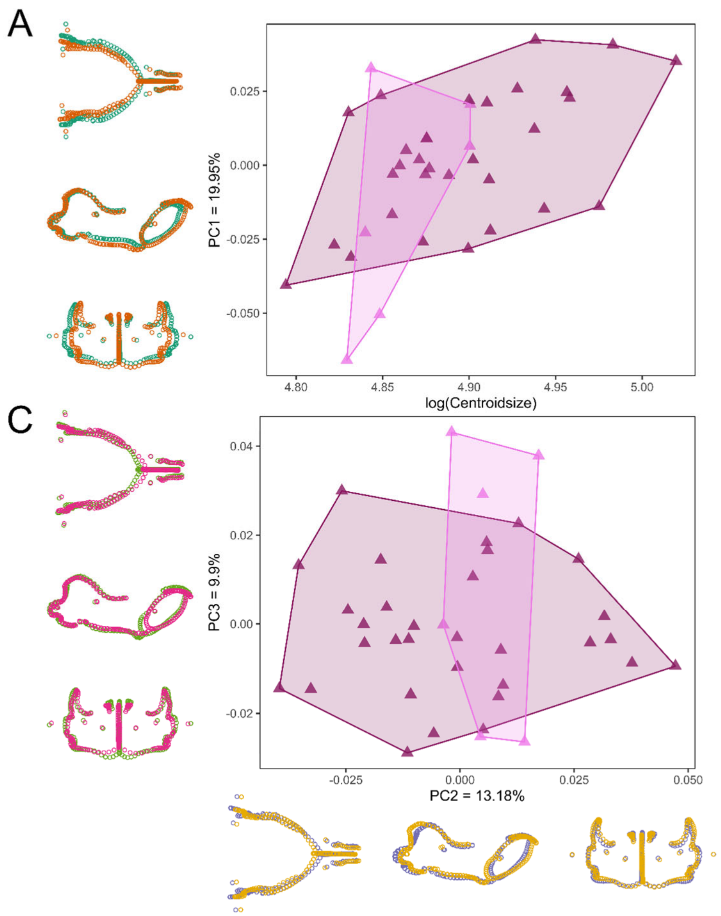

Figure S10. Ontogenetic series of females for mandibles. A. PC1 versus log-transformed Centroid size; B. Regression of Procrustes ANOVA versus log-transformed Centroid size per ontogenetic group; C. PC2 versus PC3.

There is strong overlap between juvenile and adult females on the three PC axes for mandibles (Figure S10). Only PC1 is significantly correlated with size (Table S5). PC1 is associated with shape changes in the angle between the mandibles and the depth of the ramus. PC2 is associated with shape changes in ventral margin of the ramus and the condylar, coronoid and angular processes. PC3 is associated with shape changes in the symphysis.

Table S5. Output from correlation test, pairwise comparison, MANOVA, and regression models of mandible for female ontogenetic series.

**Linear**							
	**Factor**	**p**	**cof**	**F**	**Pillai**	**df**	**den df**
**Shape ~Log Centroid size**		0.0004934		7.4763	0.37741	1	37
**Shape ~ Ontogenetic group**		0.1139		2.1228	0.14685	1	37
**Shape ~Log Centroid size * Ontogenetic group**	Size	0.0007201		7.1518	0.38004	1	35
	Group	0.2367626		1.4803	0.11259	1	35
	Size:group	0.9156397		0.1704	0.01440	1	35
**PC 1 ~ CS**		0.001689	-0.4752613				
**PC 2 ~ CS**		0.3994	-0.1351916				
**PC 3 ~ CS**		0.2455	0.1855401				
**Pairwise**		0.007					
**GMM**							
	**Factor**	**p**	**cof**	**F**	**Z**	**df**	**r2**
**Shape ~Log Centroid size**		0.01		2.9957	2.5485	1	0.7884
**Shape ~ Ontogenetic group**		0.16		1.3545	0.99012	1	0.03726
**Shape ~Log Centroid size * Ontogenetic group**	Size	0.01		3.0157	2.53278	1	0.07884
	Group	0.32		1.2091	0.62023	1	0.03161
	Size:group	0.40		1.0251	0.24662	1	0.02680
**PC 1 ~ CS**		0.001076	0.5161214				
**PC 2 ~ CS**		0.9113	0.01896633				
**PC 3 ~ CS**		0.6343	0.080844				
**Pairwise**		0.08					

Ontogeny series males

<u>Cranium</u>

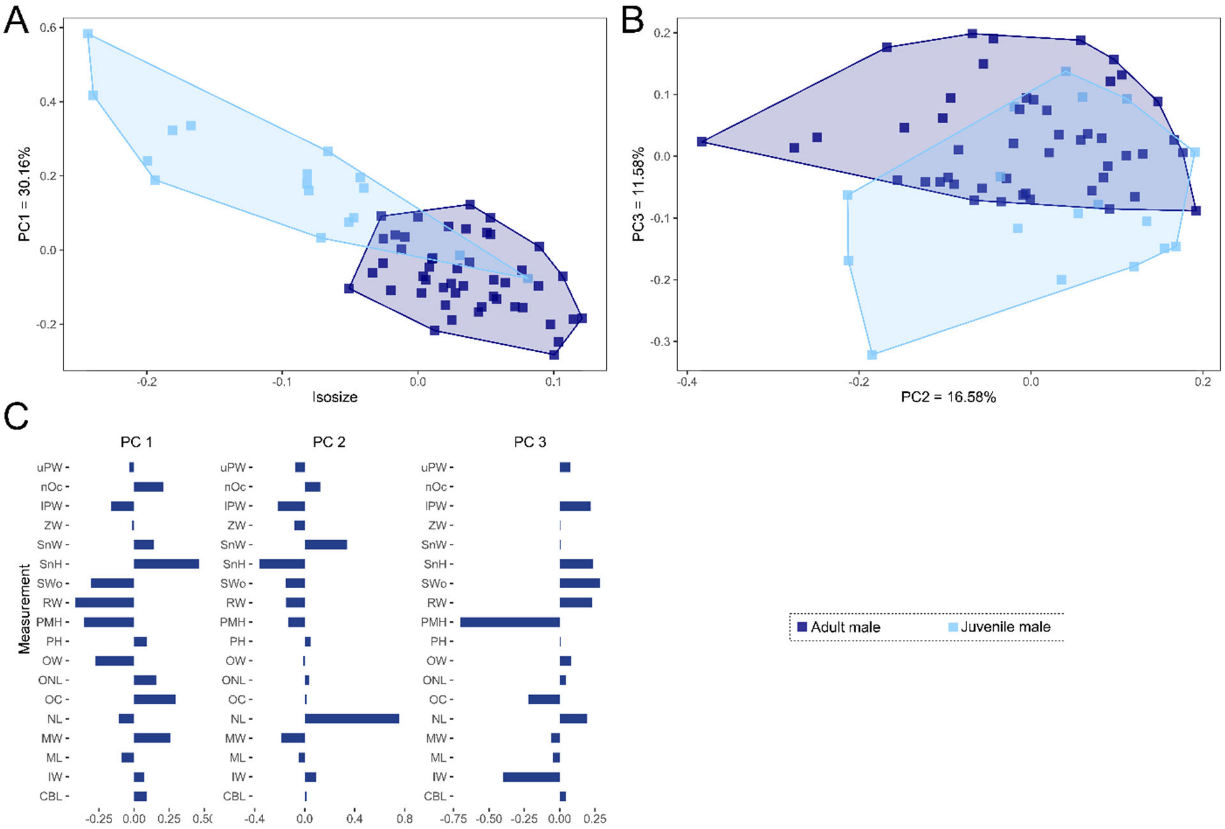

Figure S11. Ontogenetic series of males for crania. A. sPC1 versus Isosize; B. sPC2 versus sPC3; C. PCA loadings.

There is slight overlap between juvenile and adult males on sPC1 (30.16%), which is significantly correlated with size, and complete overlap on sPC2 (16.58%) and sPC3 (11.58%) (Figure S11), which are not significantly correlated with size (Table S6). The most important loadings on sPC1 are SnH, RW, PMH, SWo, and OC. The most important loadings on sPC2 are NL, SnH, SnW, IPW, and MW. The most important loadings on sPC3 are PMH, IW, SWo, SnH, and RW.

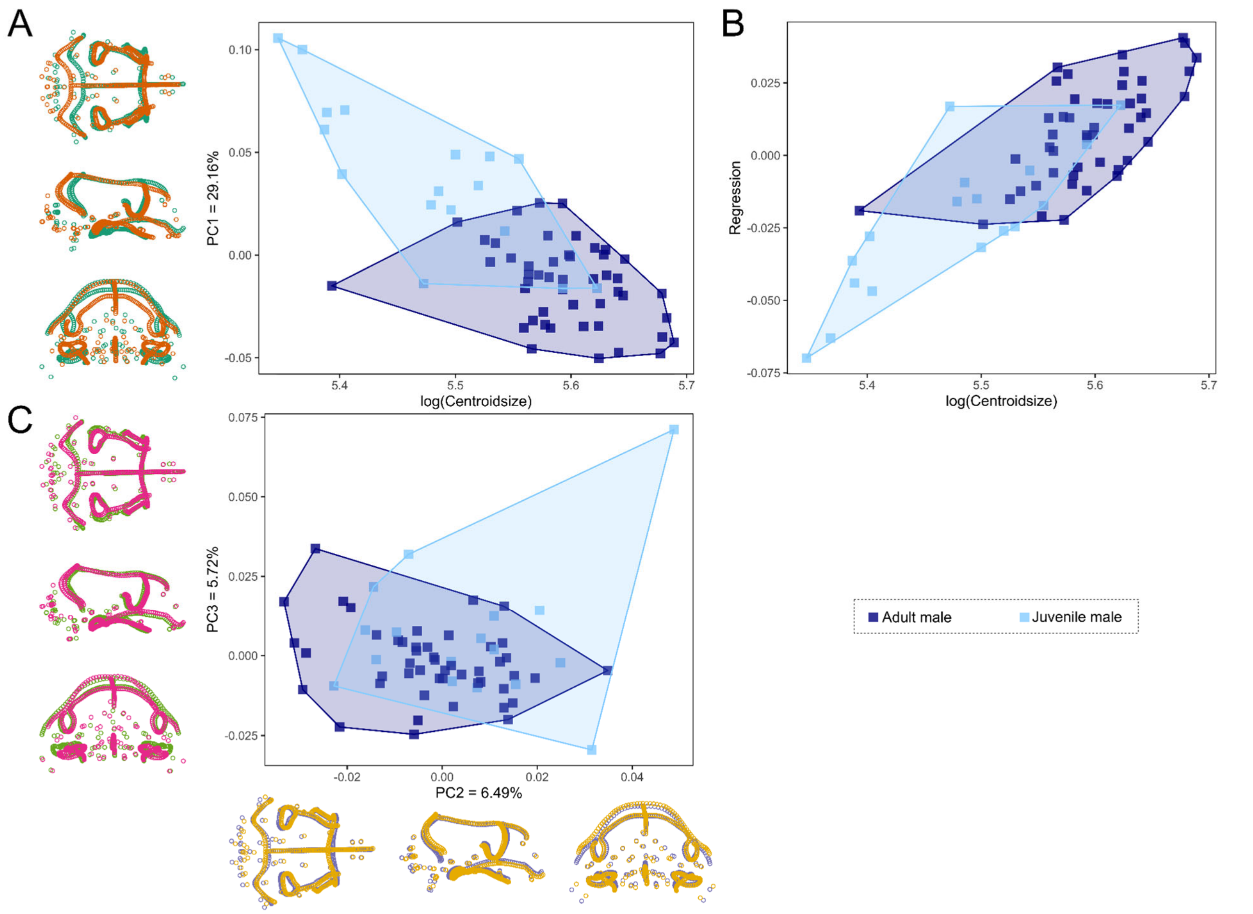

Figure S12. Ontogenetic series of males for crania. A. PC1 versus log-transformed Centroid size; B. Regression of Procrustes ANOVA versus log-transformed Centroid size per ontogenetic group; C. PC2 versus PC3.

There is little overlap between juveniles and adult males on PC1 and clear overlap on PC2 and PC3 for crania (Figure S12). Only PC1 is significantly correlated with size (Table S6). Juveniles, which have higher PC1 scores, have more posterior occipital ridge, smaller mastoid process, shorter occipital condyles and slenderer orbitals, and generally lower cranium. PC2 is associated with shape changes in the nasal ridge, mastoid process, and the occipital condyles. PC3 is associated with shape changes in the glenoid fossa, and the occipital ridge, and seems mostly driven by one individual juvenile.

Table S6. Output from correlation test, pairwise comparison, MANOVA, and regression models of cranium for male ontogenetic series.

**Linear**							
	**Factor**	**p**	**cof**	**F**	**Pillai**	**df**	**den df**
**Shape ~Log Centroid size**		<0.001		50.445	0.71272	1	61
**Shape ~ Ontogenetic group**		<0.001		50.74	0.71391	1	61
**Shape ~Log Centroid size * Ontogenetic group**	Size	<0.001		70.998	0.78308	1	59
	Group	<0.001		16.887	0.46198	1	59
	Size:group	0.0156		3.749	0.16011	1	59
**PC 1 ~ CS**		<0.001	-0.6949738				
**PC 2 ~ CS**		0.8241	0.02810315				
**PC 3 ~ CS**		0.7995	0.03212413				
**Pairwise**		<0.001					

**GMM**							
	**Factor**	**p**	**cof**	**F**	**Z**	**df**	**r2**
**Shape ~Log Centroid size**		0.01		13.673	3.3633	1	0.17833
**Shape ~ Ontogenetic group**		0.01		11.277	2.8993	1	0.15183
**Shape ~Log Centroid size * Ontogenetic group**	Size	0.01		14.3102	3.3610	1	0.17833
	Group	0.01		3.1041	2.6907	1	0.03868
	Size:group	0.02		1.8335	2.2756	1	0.2285
**PC 1 ~ CS**		<0.001	-0.6513986				
**PC 2 ~ CS**		0.6214	0.06241259				
**PC 3 ~ CS**		0.7505	-0.0402979				
**Pairwise**		<0.001					

Mandible

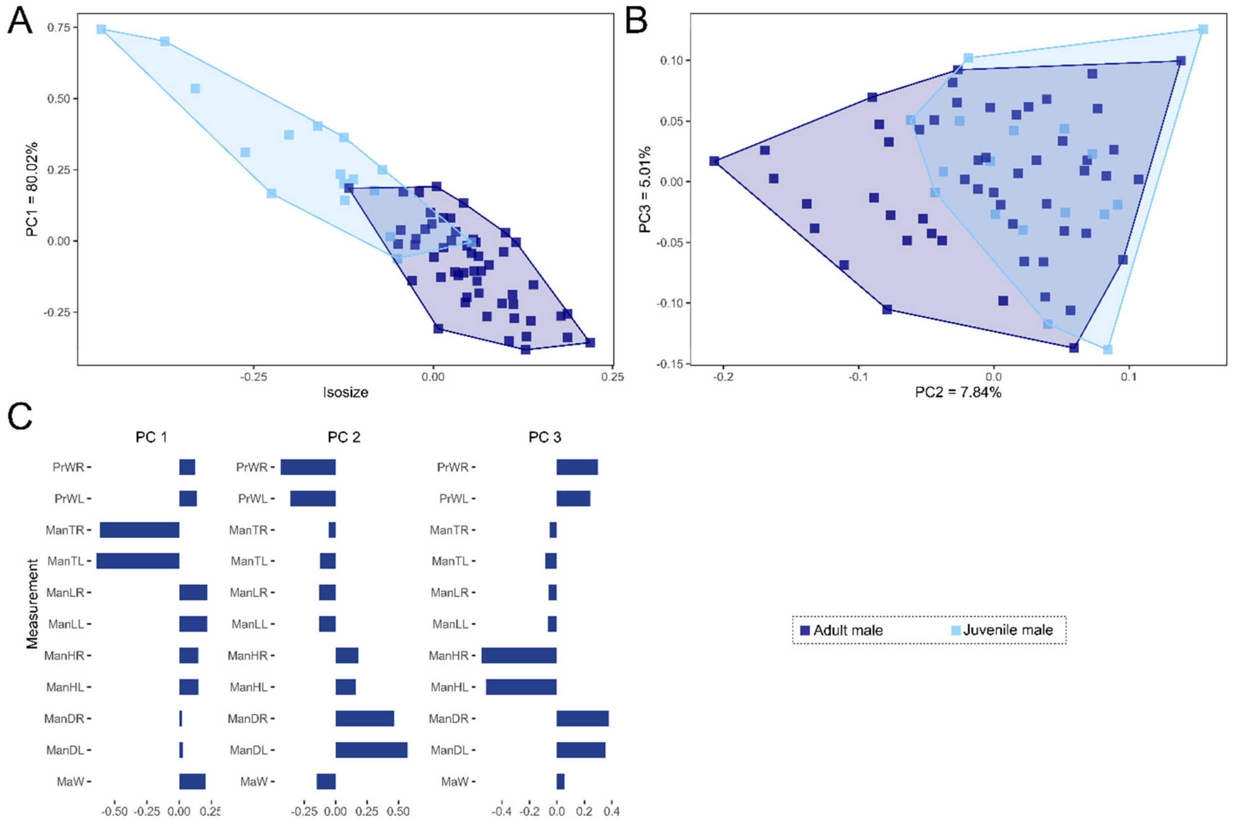

Figure S13. Ontogenetic series of males for mandibles. A. sPC1 versus Isosize; B. sPC2 versus sPC3; C. PCA loadings.

There is slight overlap between juvenile and adult males on sPC1 (80.02%), which is significantly correlated with size, and complete overlap on sPC2 (7.84%) and sPC3 (5.01%) (Figure S13), which are not significantly correlated with size (Table S7). The most important loadings on sPC 1 are ManT, ManL, and MaW. The most important loadings on sPC 2 are ManD, PrW, and ManH. The most important loadings on sPC3 are ManH, ManD, and PrW.

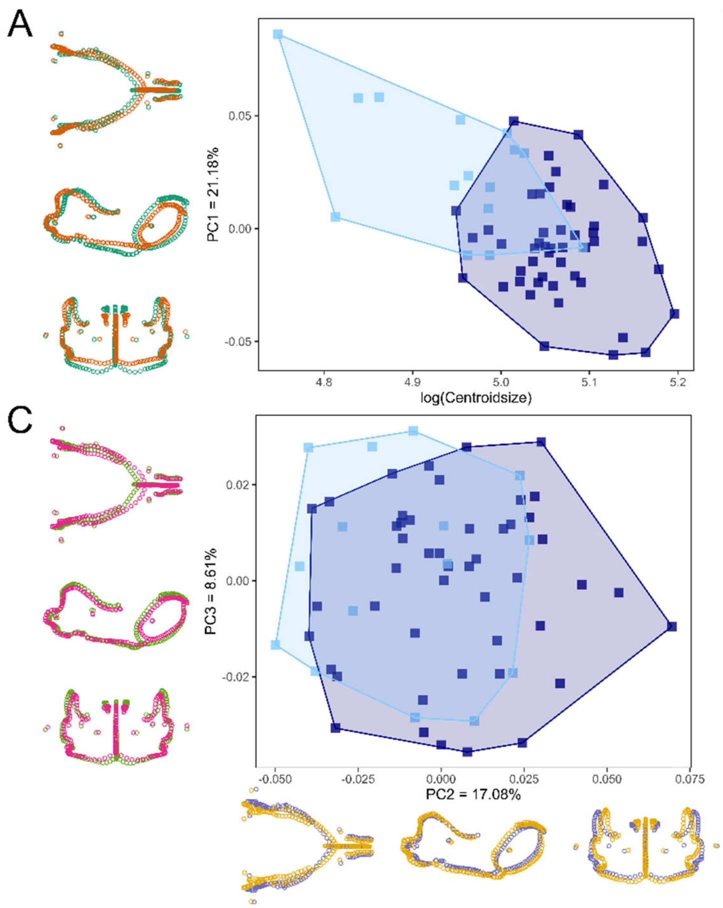

Figure S14. Ontogenetic series of females for mandibles. A. PC1 versus log-transformed Centroid size; B. Regression of Procrustes ANOVA versus log-transformed Centroid size per ontogenetic group; C. PC2 versus PC3.

There is overlap between juveniles and adult males on PC1 and complete overlap on PC2 and PC3 for mandibles (Figure S14). Only PC1 is significantly correlated with size (Table S7). Juveniles, which have higher PC1 scores, have a smaller symphysis, lower mandible, and lower angle between both mandibles. PC2 is associated with shape changes in the angular process and angle between the mandibles. PC3 is associated with slight shape changes in coronoid and condylar processes and the mental protuberance.

Table S7. Output from correlation test, pairwise comparison, MANOVA, and regression models of mandible for male ontogenetic series.

**Linear**							
	**Factor**	**p**	**cof**	**F**	**Pillai**	**df**	**den df**
**Shape ~Log Centroid size**		<0.001		93.748	0.80993	1	66
**Shape ~ Ontogenetic group**		<0.001		23.049	0.51164	1	66
**Shape ~Log Centroid size * Ontogenetic group**	Size	<0.001		92.238	0.81216	1	64
	Group	0.2618		1.364	0.06010	1	64
	Size:group	0.1229		2.000	0.8570	1	64
**PC 1 ~ CS**		<0.001	-0.8287114				
**PC 2 ~ CS**		0.8813	-0.01817864				
**PC 3 ~ CS**		0.5753	0.06811303				
**Pairwise**		<0.001					

**GMM**							
	**Factor**	**p**	**cof**	**F**	**Z**	**df**	**r2**
**Shape ~Log Centroid size**		0.01		6.8188	5.4589	1	0.10054
**Shape ~ Ontogenetic group**		0.01		5.8301	3.5554	1	0.08724
**Shape ~Log Centroid size * Ontogenetic group**	Size	0.01		6.9263	5.5075	1	0.10054
	Group	0.07		1.7424	1.5703	1	0.02529
	Size:group	0.29		1.2191	0.5659	1	0.01770
**PC 1 ~ CS**		0.002304	-0.3772561				
**PC 2 ~ CS**		0.01062	0.3197965				
**PC 3 ~ CS**		0.5058	-0.08539747				
**Pairwise**		<0.001					

Sexual dimorphism adults

Cranium

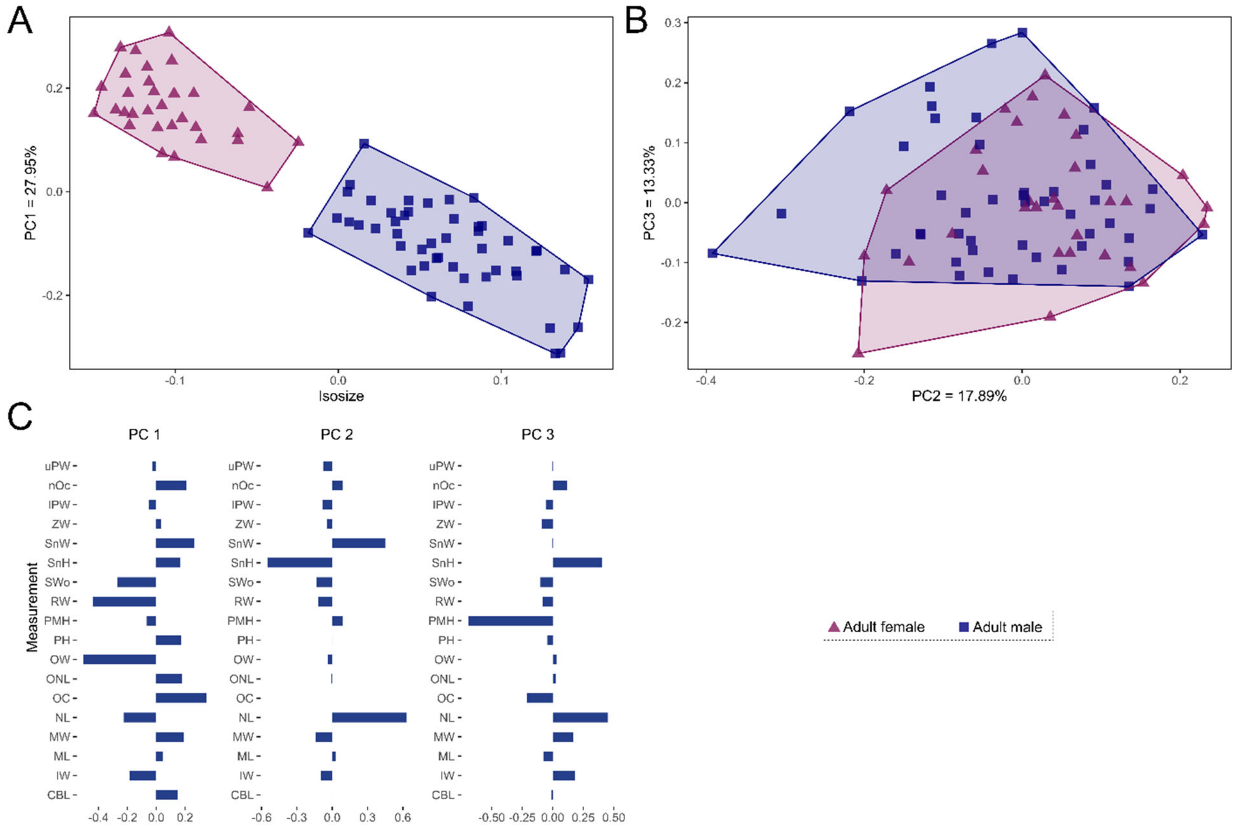

Figure S15. Sexual dimorphism in adults for crania. A. sPC1 versus Isosize; B. sPC2 versus sPC3; C. PCA loadings.

For crania, there is strong separation on sPC1 (27.95%), which is significantly correlated with size, and complete overlap on sPC2 (17.89%) and sPC3 (13.33%) (Figure S17), which are not significantly correlated with size (Table S8). The most important loadings on sPC1 are OW, RW, OC, SWo, and SnW. The most important loadings on sPC2 are NL, SnH, SnW, MW, and SWo. The most important loadings on sPC3 are PMH, NL, SnH, OC, and IW.

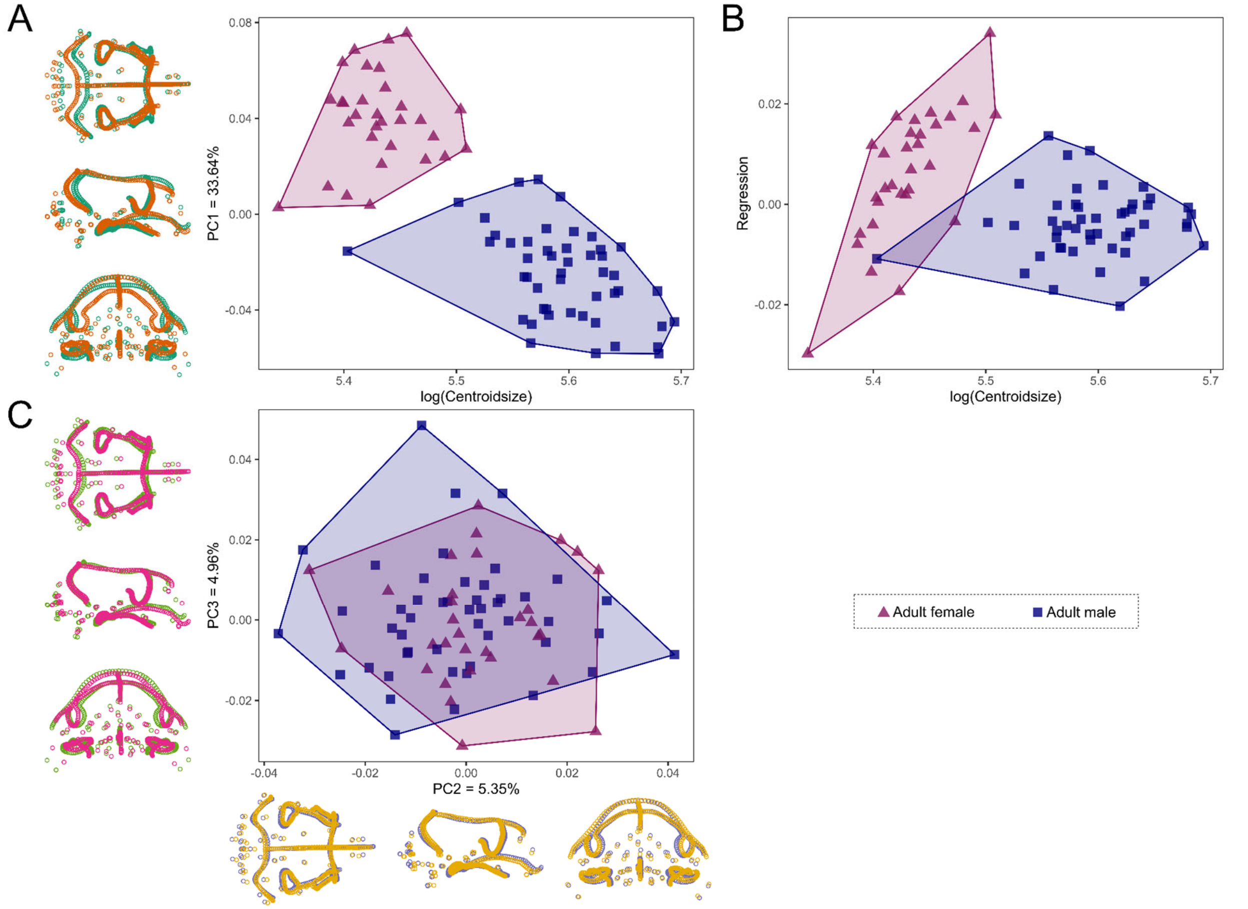

Figure S16. Sexual dimorphism of adults for crania. A. PC1 versus log-transformed Centroid size; B. Regression of Procrustes ANOVA versus log-transformed Centroid size per ontogenetic group; C. PC2 versus PC3.

There is little overlap between adult males and females on PC1, and strong overlap on PC2 and PC3 for crania (Figure S18). Only PC1 is significantly correlated with size (Table S8). Females, which have higher PC1 scores, have a relatively longer and narrower cranium, more posterior occipital ridge, lower nasal bridge, smaller mastoid processes and a more curved palate. PC2 is associated with shape changes in occipital condyles, palate and glenoid fossa. PC3 is associated with shape changes in the occipital ridge, palate, mastoid process, and the orbitals.

Table S8. Output from correlation test, pairwise comparison, MANOVA, and regression models of cranium for adult sexual dimorphism.

Linear							
	**Factor**	**p**	**cof**	**F**	**Pillai**	**df**	**den df**
**Shape ~Log Centroid size**		<0.001		156.98	0.86262	1	75
**Shape ~ Ontogenetic group**		<0.001		92.924	0.788	1	75
**Shape ~Log Centroid size * Ontogenetic group**	Size	<0.001		158.796	0.86712	1	73
	Group	0.001675		5.580	0.18655	1	73
	Size:group	0.034084		3.045	0.11123	1	73
**PC 1 ~ CS**		<0.001	-0.8977605				
**PC 2 ~ CS**		0.9179	-0.01178189				
**PC 3 ~ CS**		0.4331	0.08943525				
**Pairwise**		<0.001					

GMM							
	**Factor**	**p**	**cof**	**F**	**Z**	**df**	**r2**
**Shape ~Log Centroid size**		0.01		23.284	3.2736	1	0.23218
**Shape ~ Ontogenetic group**		0.01		26.314	3.1394	1	0.2547
**Shape ~Log Centroid size * Ontogenetic group**	Size	0.01		24.2802	3.3021	1	0.23218
	Group	0.01		4.1674	3.4534	1	0.03985
	Size:group	0.37		1.1259	0.3936	1	0.01077
**PC 1 ~ CS**		<0.001	-0.7927702				
**PC 2 ~ CS**		0.03969	-0.231962				
**PC 3 ~ CS**		0.5583	0.06684518				
**Pairwise**		<0.001					

Mandible

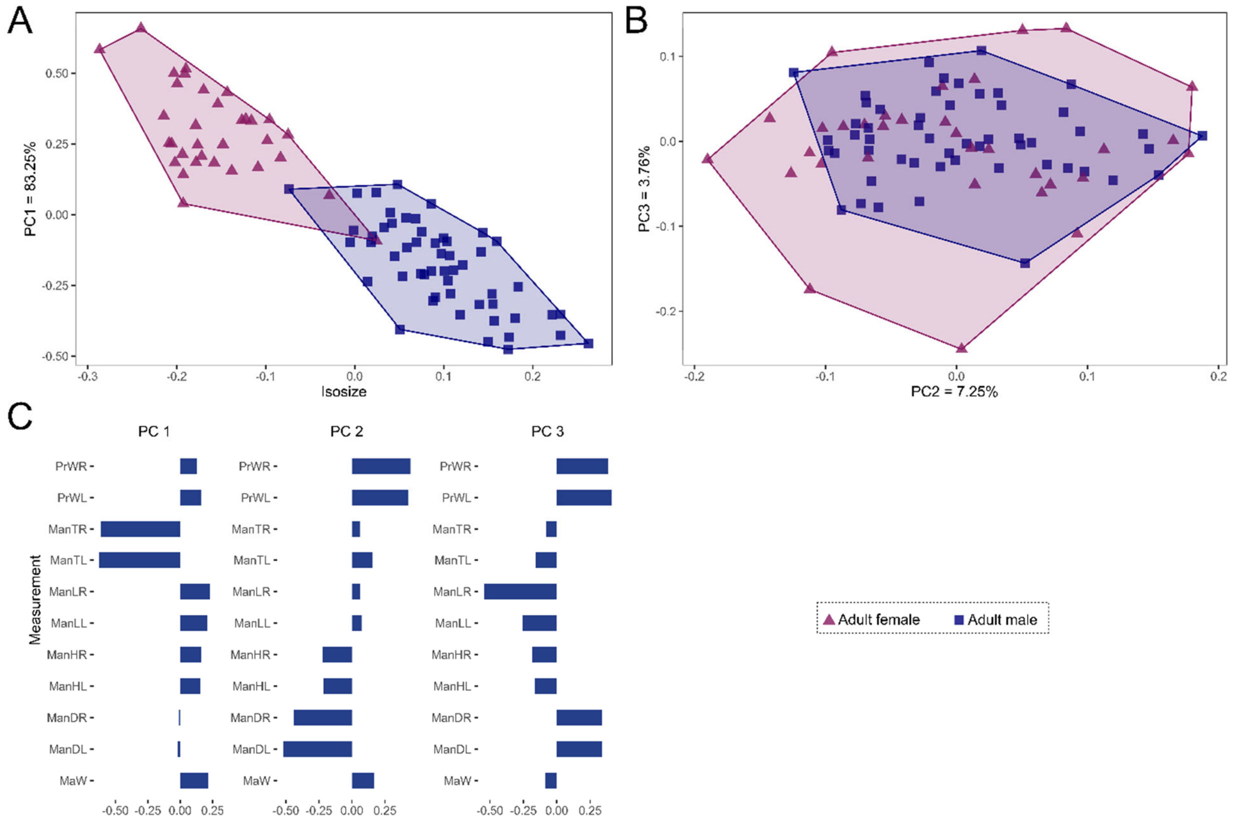

Figure S17. Sexual dimorphism in adults for mandibles. A. sPC1 versus Isosize; B. sPC2 versus sPC3; C. PCA loadings.

For mandibles, there is overlap on sPC1 (83.25%), which is significantly correlated with size, and complete overlap on sPC2 (7.25%) and sPC3 (3.76%) for mandibles (Figure S17), which are not significantly correlated with size (Table S9). The most important loadings on sPC 1 are ManT, ManL, and MaW. The most important loadings on sPC 2 are ManD, PrW, and ManH. The most important loadings on sPC3 are ManL, PrW, and ManD.

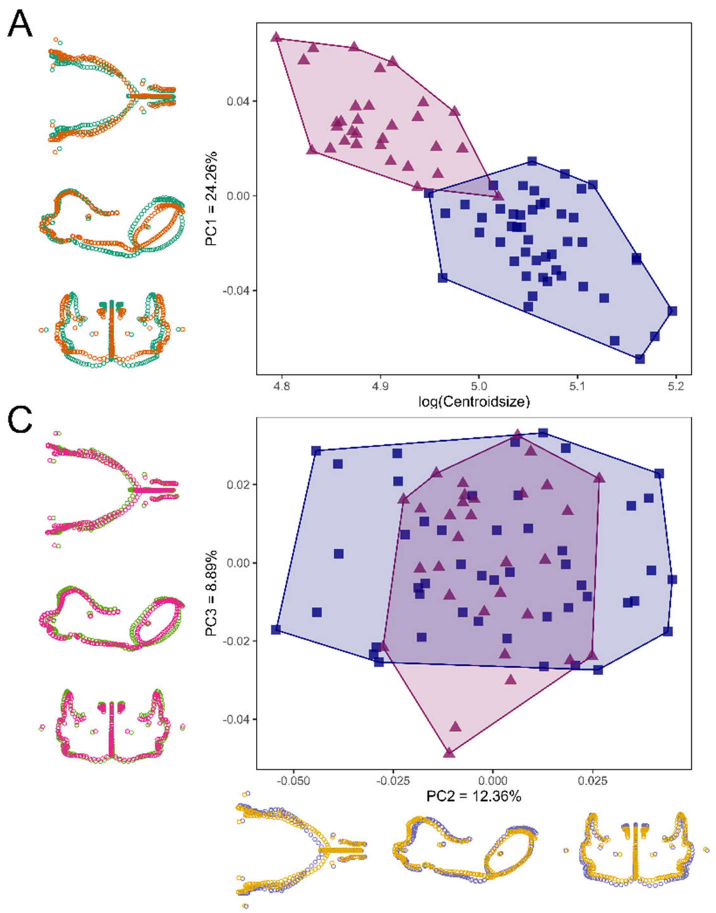

Figure S18. Sexual dimorphism of adults for mandibles. A. PC1 versus log-transformed Centroid size; B. Regression of Procrustes ANOVA versus log-transformed Centroid size per ontogenetic group; C. PC2 versus PC3.

There is little overlap between adult males and females on PC1, and strong overlap on PC2 and PC3 for mandibles (Figure S18). Only PC1 is significantly correlated with size (Table S9). Females, which have higher PC1 scores, have a relatively smaller symphysis, lower mandible, and wider angle between the mandibles. PC2 is associated with shape changes in ventral protrusion of the ramus, the angle between the mandibles, the angle of the ramus, and the shape of the coronoid process. PC3 is associated with shape changes in the condylar process. The mandible shape of adult females and males is clearly distinct and shows clear allometry.

Table S9. Output from correlation test, pairwise comparison, MANOVA, and regression models of mandible for adult sexual dimorphism.

**Linear**							
	**Factor**	**p**	**cof**	**F**	**Pillai**	**df**	**den df**
**Shape ~Log Centroid size**		<0.001		132.35	0.82883	1	82
**Shape ~ Ontogenetic group**		<0.001		62.403	0.6954	1	82
**Shape ~Log Centroid size * Ontogenetic group**	Size	<0.001		131.950	0.83188	1	80
	Group	0.3241		1.176	0.04225	1	80
	Size:group	0.4138		0.964	0.03490	1	80
**PC 1 ~ CS**		<0.001	-0.8851078				
**PC 2 ~ CS**		0.6646	-0.04742677				
**PC 3 ~ CS**		0.2393	0.1282419				
**Pairwise**		<0.001					

**GMM**							
	**Factor**	**p**	**cof**	**F**	**Z**	**df**	**r2**
**Shape ~Log Centroid size**		0.01		16.549	3.5708	1	0.1769
**Shape ~ Ontogenetic group**		0.01		15.327	3.9514	1	0.16601
**Shape ~Log Centroid size * Ontogenetic group**	Size	0.01		16.8900	3.5906	1	0.17690
	Group	0.01		2.2534	2.2518	1	0.02360
	Size:group	0.15		1.3356	1.1162	1	0.01399
**PC 1 ~ CS**		<0.001	-0.8063291				
**PC 2 ~ CS**		0.5871	-0.06202532				
**PC 3 ~ CS**		0.6908	-0.04544791				
**Pairwise**		<0.001					

Sexual dimorphism juveniles

Cranium

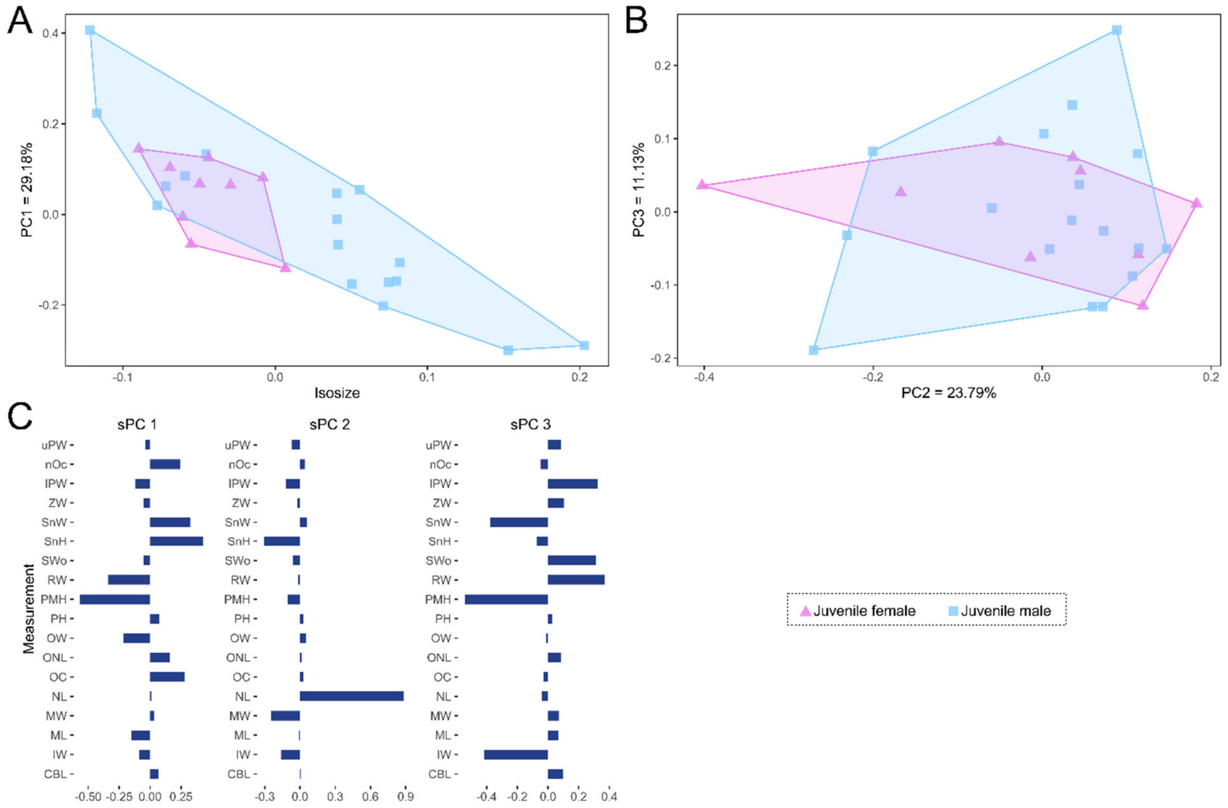

Figure S19. Sexual dimorphism in juveniles for crania. A. sPC1 versus Isosize; B. sPC2 versus sPC3; C. PCA loadings.

For crania, there is complete overlap on sPC1 (29.18%), which is significantly correlated with size, and complete overlap on sPC2 (23.79%) and sPC3 (11.13%) (Figure S19), which are not significantly correlated with size (Table S10). The most important loadings on sPC1 are PMH, SnH, RW, SnW, and OC. The most important loadings on sPC2 are NL, SnH, MW, IW, and IPW. The most important loadings on sPC3 are PMH, IW, SnW, RW, and IPW. The cranium shape difference between juvenile males and females is minimal.

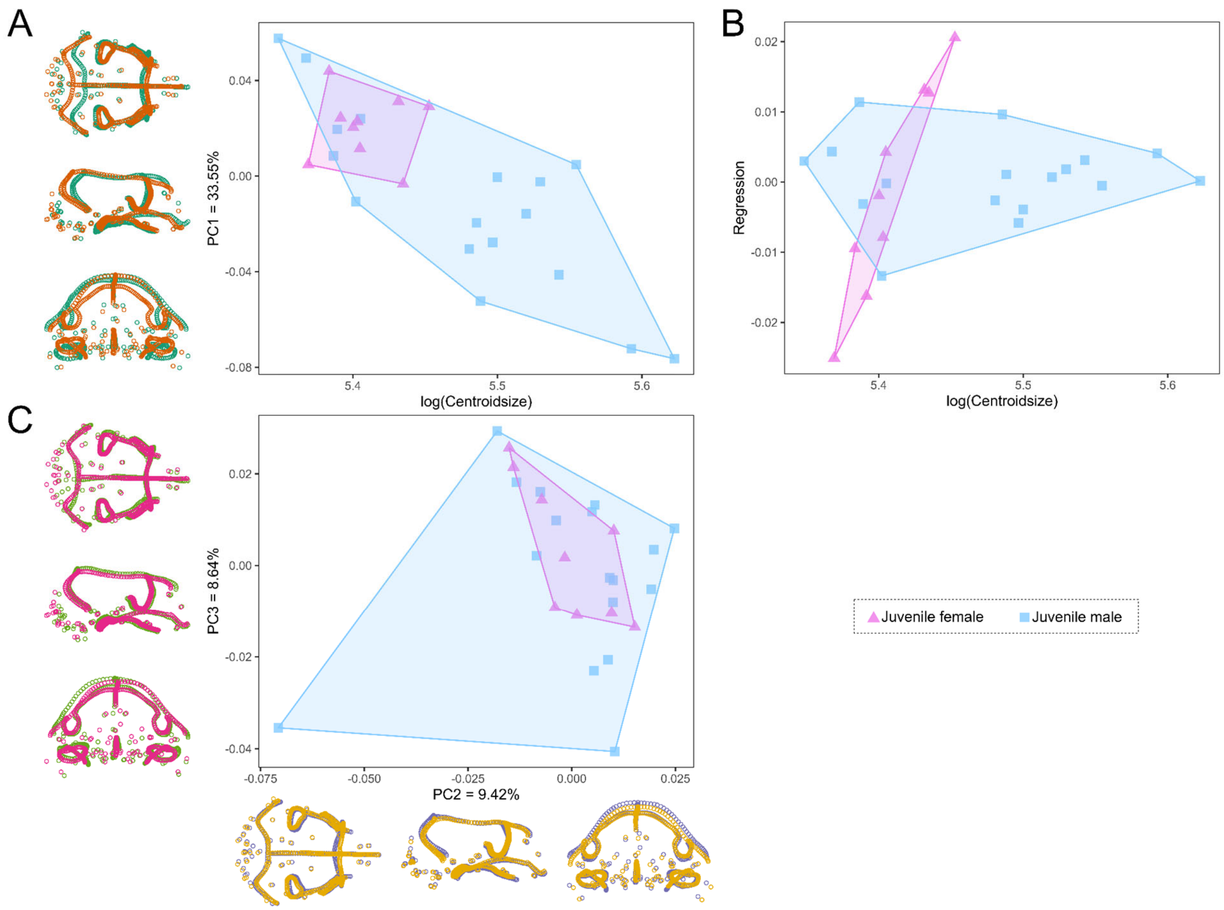

Figure S20. Sexual dimorphism of juveniles for crania. A. PC1 versus log-transformed Centroid size; B. Regression of Procrustes ANOVA versus log-transformed Centroid size per ontogenetic group; C. PC2 versus PC3.

There is complete overlap between juvenile males and females on PC1, PC2 and PC3 for crania (Figure S20). Only PC1 is significantly correlated with size (Table S10). PC1 is associated with shape changes in the occipital ridge, nasal ridge, occipital condyles, and general height of the cranium. PC2 is associated with shape changes in the height of the cranium and nasal ridge. PC3 is associated with shape changes in the orbit, occipital ridge and occipital condyles. The cranium shape difference between juvenile males and females is minimal.

Table S10. Output from correlation test, pairwise comparison, MANOVA, and regression models of cranium for juvenile sexual dimorphism.

**Linear**							
	**Factor**	**p**	**cof**	**F**	**Pillai**	**df**	**den df**
**Shape ~Log Centroid size**		<0.001		20.694	0.73835	1	22
**Shape ~ Ontogenetic group**		0.7639		0.3864	0.050053	1	22
**Shape ~Log Centroid size * Ontogenetic group**	Size	<0.001		20.6902	0.75631	1	20
	Group	0.54559		0.7310	0.09881	1	20
	Size:group	0.05927		2.9186	0.30449	1	20
**PC 1 ~ CS**		<0.001	-0.8071795				
**PC 2 ~ CS**		0.7843	-0.05641026				
**PC 3 ~ CS**		0.7287	-0.07145299				
**Pairwise**		0.085					

**GMM**							
	**Factor**	**p**	**cof**	**F**	**Z**	**df**	**r2**
**Shape ~Log Centroid size**		0.01		7.4574	2.9338	1	0.23706
**Shape ~ Ontogenetic group**		0.03		2.5645	2.0479	1	0.09654
**Shape ~Log Centroid size * Ontogenetic group**	Size	0.01		7.4892	2.87029	1	0.23706
	Group	0.19		1.2470	0.93919	1	0.03947
	Size:group	0.61		0.8552	-0.20753	1	0.02707
**PC 1 ~ CS**		<0.001	-0.7545299				
**PC 2 ~ CS**		0.4163	0.1664957				
**PC 3 ~ CS**		0.2806	0.2198291				
**Pairwise**		0.051					

Mandible

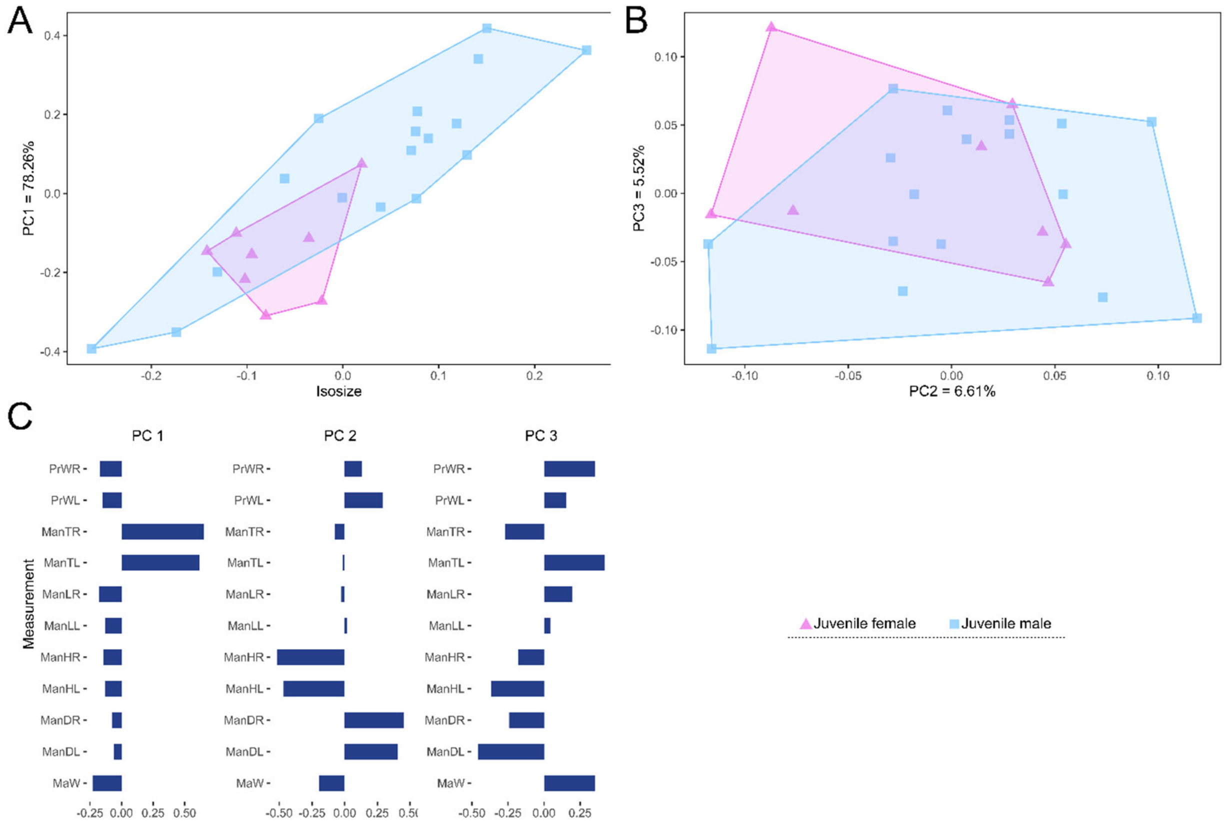

Figure S21. Sexual dimorphism in juveniles for mandibles. A. sPC1 versus Isosize; B. sPC2 versus sPC3; C. PCA loadings.

For mandibles, there is complete overlap on sPC1 (78.26%), which is significantly correlated with size, and complete overlap on sPC2 (6.61%) and sPC3 (5.52%) (Figure S21), which are not significantly correlated with size (Table S11). The most important loadings on sPC 1 are ManT, MaW, and ManL. The most important loadings on sPC 2 are ManH, ManD, and PrW. The most important loadings on sPC3 are ManD, ManT, ManH. The mandible shape difference between juvenile males and females is minimal.

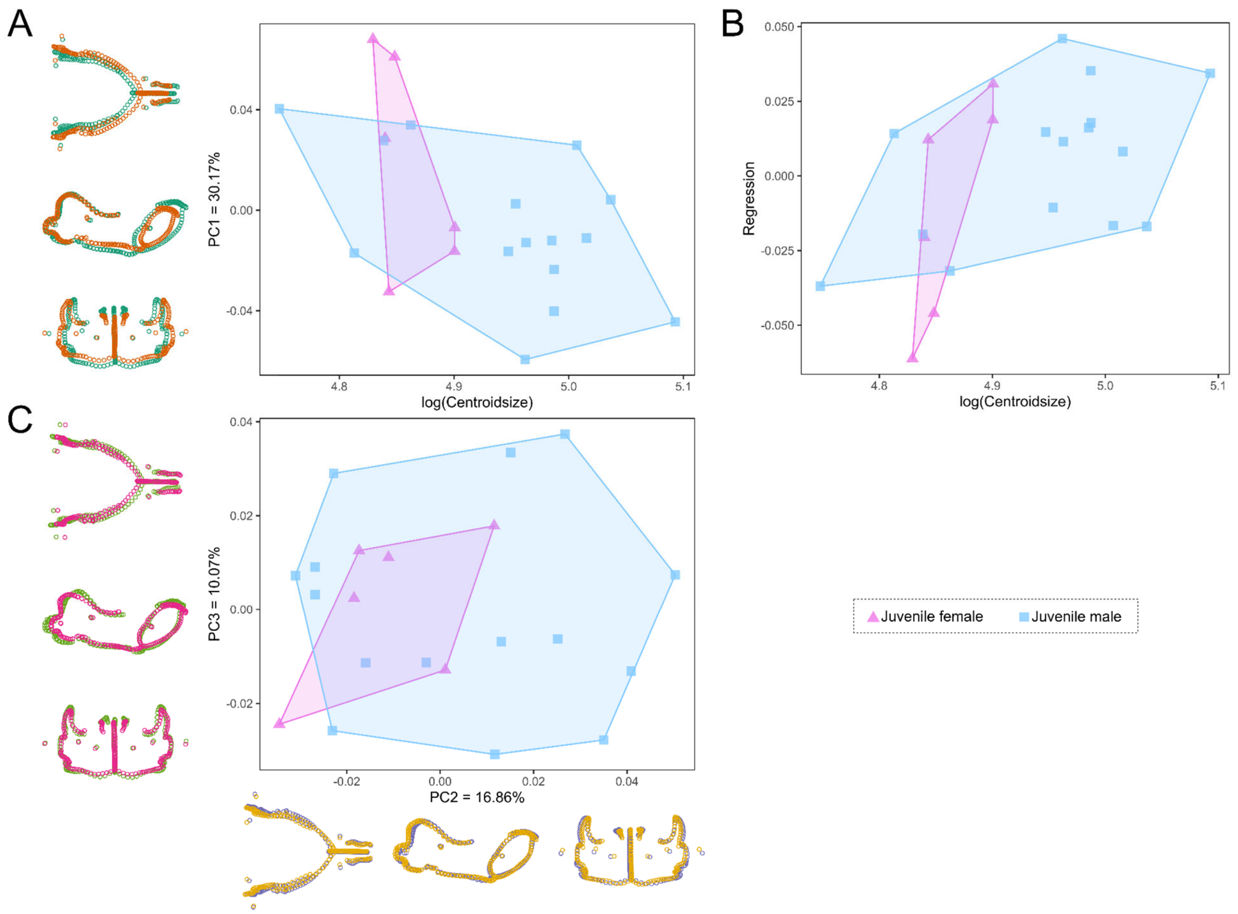

Figure S22. Sexual dimorphism of juveniles for mandibles. A. PC1 versus log-transformed Centroid size; B. Regression of Procrustes ANOVA versus log-transformed Centroid size per ontogenetic group; C. PC2 versus PC3.

There is strong overlap between juvenile males and females on PC1, PC2 and PC3 for mandibles (Figure S22). Only PC1 is significantly correlated with size (Table S11). PC1 is associated with shape changes in the symphysis, ventral protrusion of the ramus, and angle between the mandibles. PC2 is associated with shape changes in the coronoid process. PC3 is associated with shape changes in the coronoid, condylar and angular processes and the mental protuberance. The mandible shape difference between juvenile males and females is minimal.

Table S11. Output from correlation test, pairwise comparison, MANOVA, and regression models of mandible for juvenile sexual dimorphism.

**Linear**							
	**Factor**	**p**	**cof**	**F**	**Pillai**	**df**	**den df**
**Shape ~Log Centroid size**		<0.001		24.077	0.77475	1	21
**Shape ~ Ontogenetic group**		0.09544		2.4114	0.25622	1	21
**Shape ~Log Centroid size * Ontogenetic group**	Size	<0.001		24.9211	0.79736	1	19
	Group	0.5224		0.7748	0.10900	1	19
	Size:group	0.7856		0.3556	0.05317	1	19
**PC 1 ~ CS**		<0.001	0.8538462				
**PC 2 ~ CS**		0.6768	0.08769231				
**PC 3 ~ CS**		0.8897	-0.02923077				
**Pairwise**		0.023					

**GMM**							
	**Factor**	**p**	**cof**	**F**	**Z**	**df**	**r2**
**Shape ~Log Centroid size**		0.02		2.669	2.1745	1	0.12317
**Shape ~ Ontogenetic group**		0.16		1.411	1.158	1	0.06913
**Shape ~Log Centroid size * Ontogenetic group**	Size	0.02		2.6253	2.11632	1	0.12317
	Group	0.57		0.8355	-0.11710	1	0.03920
	Size:group	0.50		0.8532	-0.00091	1	0.04003
**PC 1 ~ CS**		0.04368	-0.4441558				
**PC 2 ~ CS**		0.8624	0.04025974				
**PC 3 ~ CS**		0.533	-0.1441558				
**Pairwise**		0.045					

Comparison adult females with juvenile males

Cranium

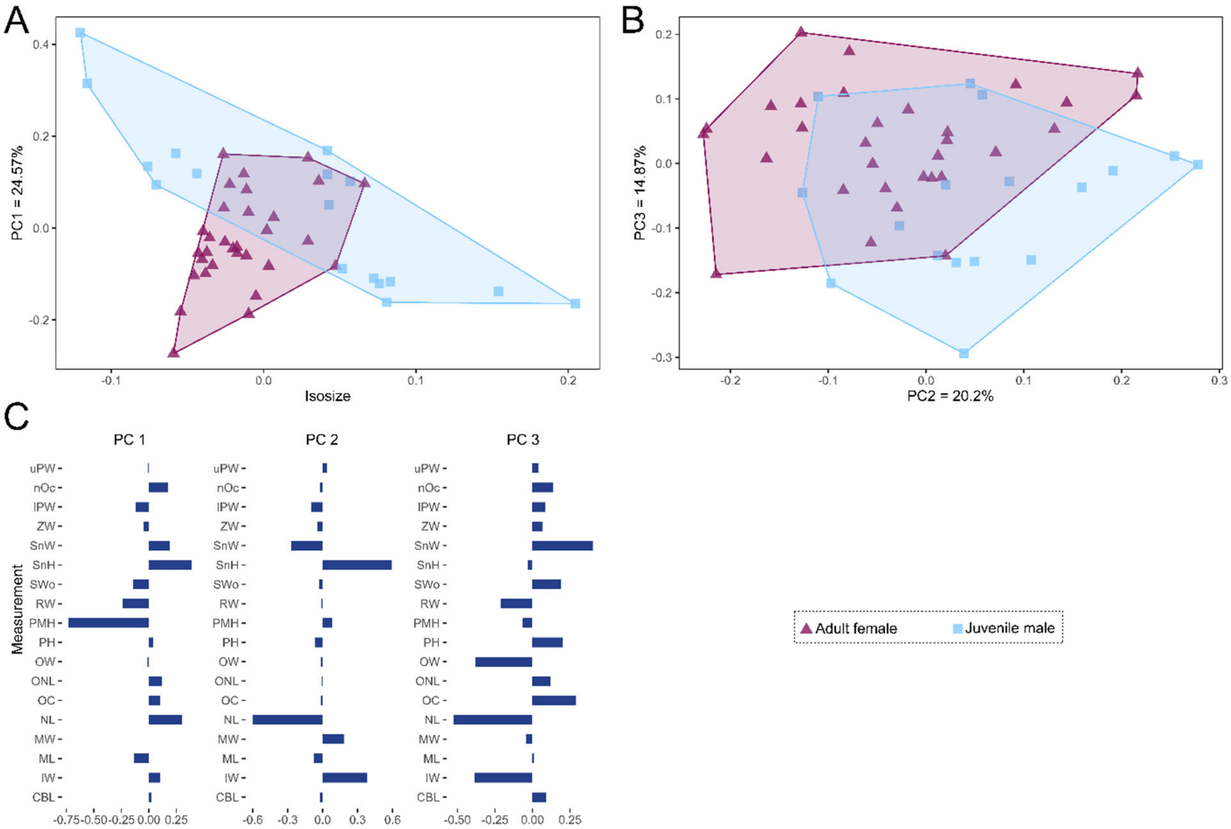

Figure S23. Comparison of adult females and juvenile males for crania. A. sPC1 versus Isosize; B. sPC2 versus sPC3; C. PCA loadings.

There is complete overlap in sPC1 (24.57%), sPC2 (20.2%) and sPC3 (14.87%) for crania (Figure S23). Only sPC3 is significantly correlated with size (Table S12). The most important loadings on sPC1 are PMH, SnH, NL, RW, and nOc. The most important loadings on sPC2 are NL, SnH, IW, SnW, and MW. The most important loadings on sPC3 are NL, OW, IW, SnW, and OC.

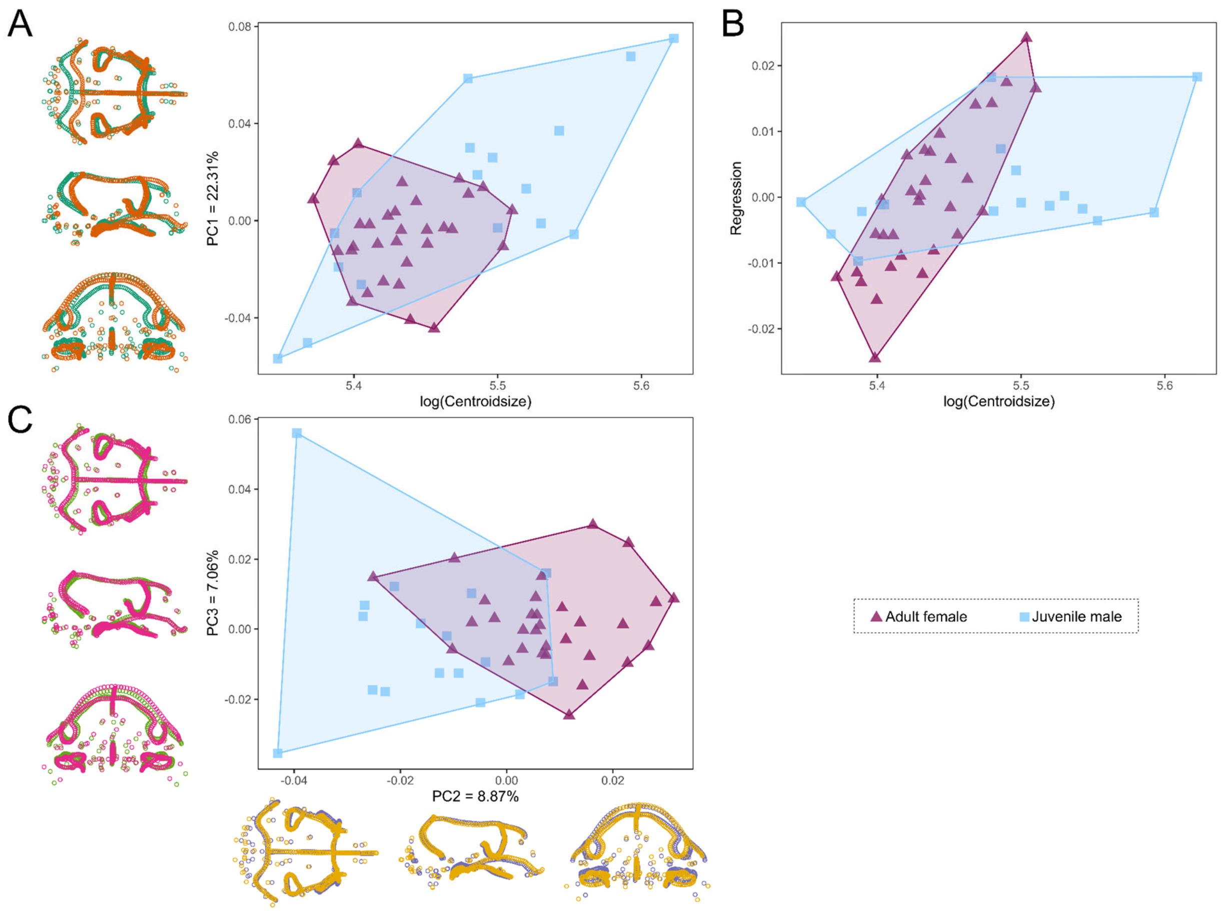

Figure S24. Comparison of adult females and juvenile males for crania. A. PC1 versus log-transformed Centroid size; B. Regression of Procrustes ANOVA versus log-transformed Centroid size per ontogenetic group; C. PC2 versus PC3.

There is complete overlap in PC1 and PC3 and slight overlap in PC2 for crania (Figure S24). Only PC1 is significantly correlated with size (Table S12). PC1 is associated with shape changes in occipital ridge, nasal ridge, occipital condyles, nasal aperture, and orbitals. Adult females, which have higher PC2 scores, have slightly slenderer orbitals, more protruding occipital condyles, more curved palates and more pronounced glenoid fossa compared with juvenile males. PC3 is associated with shape changes in occipital ridge curvature and mastoid process.

Table S12. Output from correlation test, pairwise comparison, MANOVA, and regression models of cranium for comparison of adult females and juvenile males.

**Linear**							
	**Factor**	**p**	**cof**	**F**	**Pillai**	**df**	**den df**
**Shape ~Log Centroid size**		<0.001		18.209	0.55387	1	44
**Shape ~ Ontogenetic group**		0.0001915		8.1976	0.35853	1	44
**Shape ~Log Centroid size * Ontogenetic group**	Size	<0.001		17.4711	0.55515	1	42
	Group	0.0001513		8.5469	0.37907	1	42
	Size:group	<0.001		10.9734	0.43940	1	42
**PC 1 ~ CS**		0.06619	-0.2673686				
**PC 2 ~ CS**		0.8951	-0.01953973				
**PC 3 ~ CS**		<0.001	-0.6098567				
**Pairwise**		0.12					

**GMM**							
	**Factor**	**p**	**cof**	**F**	**Z**	**df**	**r2**
**Shape ~Log Centroid size**		0.01		5.1714	3.5224	1	0.10106
**Shape ~ Ontogenetic group**		0.01		3.3852	3.0701	1	0.06855
**Shape ~Log Centroid size * Ontogenetic group**	Size	0.01		5.4968	3.6065	1	0.010106
	Group	0.01		2.9792	3.6932	1	0.05477
	Size:group	0.01		1.9155	2.7961	1	0.03522
**PC 1 ~ CS**		0.0004047	0.4902301				
**PC 2 ~ CS**		0.3613	-0.1347156				
**PC 3 ~ CS**		0.6939	0.05829353				
**Pairwise**		0.077					

Mandible

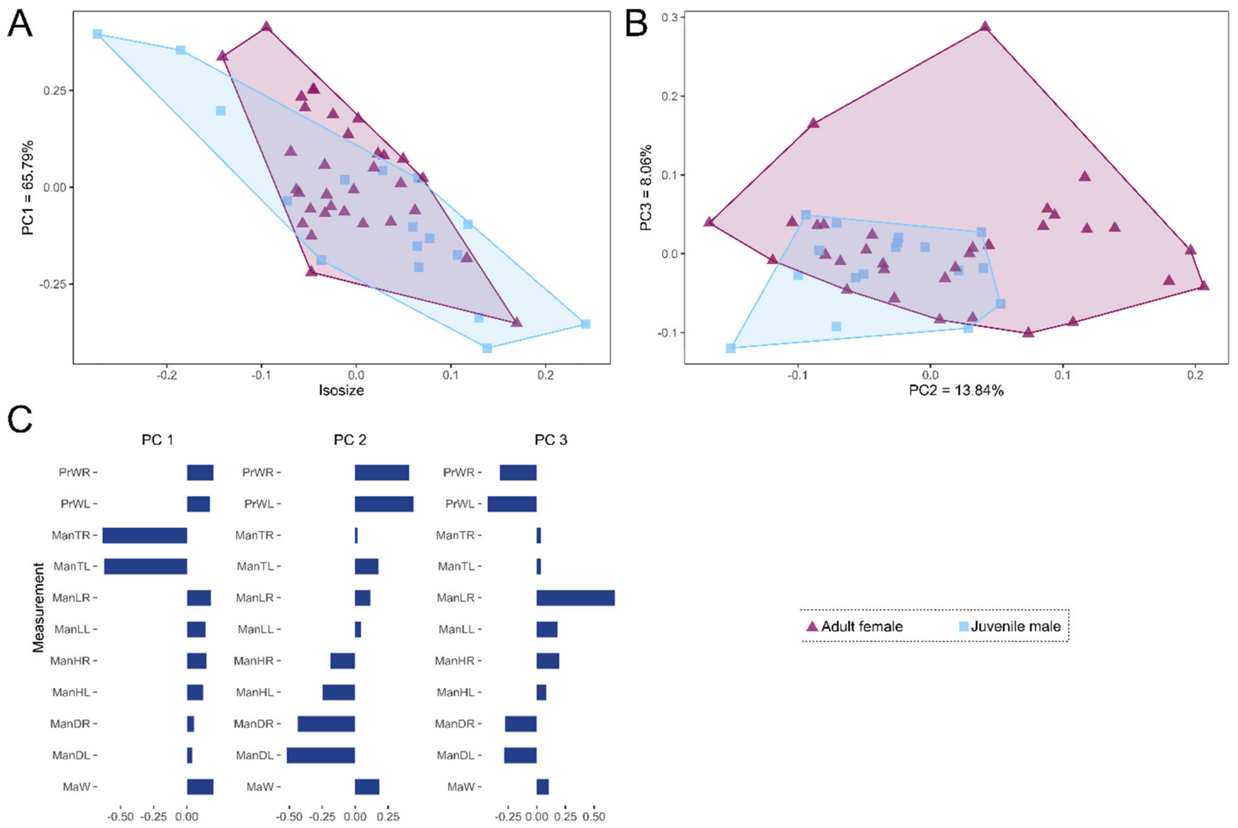

Figure S25. Comparison of adult females and juvenile males for mandibles. A. sPC1 versus Isosize; B. sPC2 versus sPC3; C. PCA loadings.

There is complete overlap between juvenile males and adult females on sPC1 (65.79%), sPC2 (13.84%), and sPC3 (8.06%) (Figure S25). Only sPC1 is significantly correlated with size (Table S13). The most important loadings on sPC1 are ManT, MaW, and PrW. The most important loadings on sPC2 are ManD, PrW, and ManH. The most important loadings on sPC3 are ManL, PrW, and ManD.

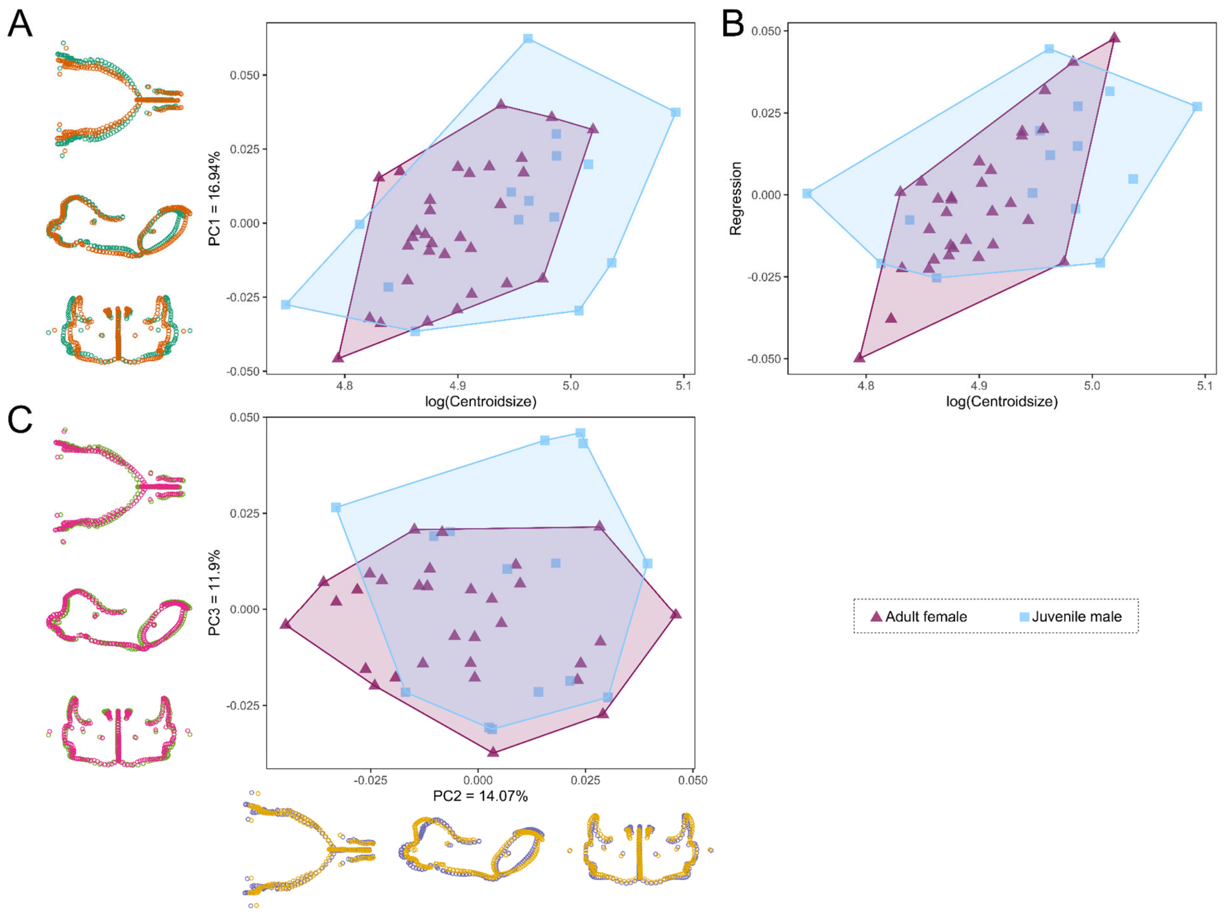

Figure S26. Comparison of adult females and juvenile males for mandibles. A. PC1 versus log-transformed Centroid size; B. Regression of Procrustes ANOVA versus log-transformed Centroid size per ontogenetic group; C. PC2 versus PC3.

There is complete overlap between juvenile males and adult females on PC1, and PC2 and strong overlap in PC3 (Figure S26). Both PC1 and PC2 are significantly correlated with size (Table S13). PC1 is associated with shape changes in the ventral margin of the ramus, the angle between the mandibles, and the symphysis. PC2 is associated with shape changes in the condylar, coronoid and angular processes. PC3 is associated with slight shape changes in the ventral protrusion of the ramus and angle between the mandibles.

Table S13. Output from correlation test, pairwise comparison, MANOVA, and regression models of mandible for comparison of adult females and juvenile males.

**Linear**							
	**Factor**	**p**	**cof**	**F**	**Pillai**	**df**	**den df**
**Shape ~Log Centroid size**		<0.001		20.235	0.5689	1	46
**Shape ~ Ontogenetic group**		0.02228		3.5175	0.1866	1	46
**Shape ~Log Centroid size * Ontogenetic group**	Size	<0.001		20.6508	0.58472	1	44
	Group	0.04365		2.9358	0.16678	1	44
	Size:group	0.61675		0.6026	0.03947	1	44
**PC 1 ~ CS**		<0.001	-0.6116206				
**PC 2 ~ CS**		0.7634	-0.04364946				
**PC 3 ~ CS**		0.2663	-0.1602401				
**Pairwise**		0.071					

**GMM**							
	**Factor**	**p**	**cof**	**F**	**Z**	**df**	**r2**
**Shape ~Log Centroid size**		0.01		3.6035	2.8445	1	0.0757
**Shape ~ Ontogenetic group**		0.01		2.1896	2.489	1	0.04741
**Shape ~Log Centroid size * Ontogenetic group**	Size	0.01		3.6849	2.87870	1	0.07570
	Group	0.02		1.8331	2.15085	1	0.03766
	Size:group	0.26		1.1615	0.68276	1	0.02386
**PC 1 ~ CS**		0.0001599	0.5284613				
**PC 2 ~ CS**		0.02616	0.3277829				
**PC 3 ~ CS**		0.6422	0.07036694				
**Pairwise**		0.018					

Socket linear measurements analysis

Using a subset of the specimens with tusk sockets intact or accessible, a morphological analysis was done for the internal tusk socket measurements similarly to what was done for the whole crania and mandibles in the main text. Sufficient specimens were only available for adult females, adult males, and juvenile females. The longitudinal (maximum dimension in anteroposterior direction) and latitudinal (maximum dimension in lateral-medial direction) measurements were taken for both left and right tusk socket roots were possible using spring calipers (Figure S27). Geometric morphometrics was not used for analysis, as the inside of the tusk sockets were not possible to scan. The dataset (Table S21 in Supplementary Information 2) and Rscript used for the analysis are available in the supplementary information.

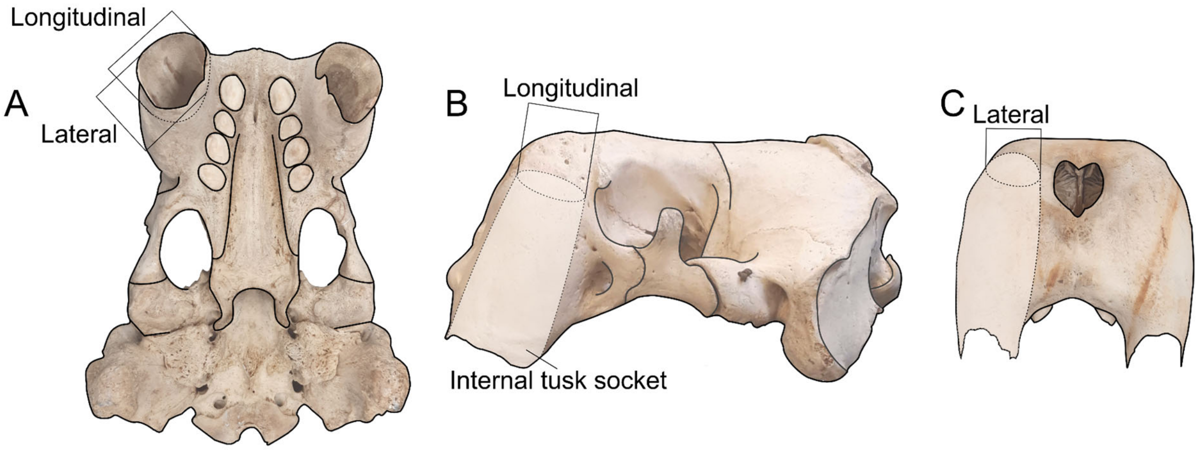

Figure S27. Measurements taken on the internal tusk socket roots. A. ventral view; B. lateral view; C. anterior view.

60 specimens of the ontogenetic reference dataset have at least one internal tusk socket measurement and were included in the analysis. Each measurement was present in at least 56 specimens. Across the ontogenetic groups, there were 24 adult females, 6 juvenile females, 28 adult males, 1 juvenile male, and 1 neonate male. Descriptive statistics for these groups are available in Table S14. Only adult females, juvenile females, and adult males are further considered in the size and shape analysis and for the classification test.

Adult males are larger than juvenile females and adult females in isosize and each individual measurement, while juvenile and adult females overlap in size and measurement ranges (Figure S28, Table S14). All groups overlap on the PC axes.
Two specimens (one adult male, FOC4509, and one adult female, FOC4610) seem to be outliers on PC axes (PC1 and PC2 respectively). The female specimen was also visually observed to have a noticeably different shape of the tusk socket. The male specimen could potentially reflect an error in measuring.

A boot-strapped LDA on all three ontogenetic groups showed a mean accuracy of 72.72%, which can be explained by the overlap in size and shape between adult and juvenile females. A boot-

strapped LDA on only adult females and males showed a mean accuracy of 96.75%. This shows that the internal tusk socket measurements are highly suitable for sex determination due to the large size difference between males and females, if the age of specimens can be confirmed. For adult (and thus large) males, this approach can be useful on size alone, while for adult females and juveniles, the age needs to be determined first via suture fusion states. The latter option can be difficult when working with incomplete and fragmented specimens, although nasal and/or premaxilla sutures are usually mostly or fully fused in (sub)adult specimens and often visible when internal tusk socket measurements are available, as is for example the case for rostra.

The classification of 7 unknown adult specimens was done using the same LDA approach as done for whole crania and mandibles, using the reference subset of 60 specimens. All specimens were classified as males, and were also classified as males by linear and geometric morphometrics using whole crania and mandibles. DNA sexing confirmed five of these to be male and two were inconclusive.

These results confirm the findings in Barrett *et al.* (2020) that the internal tusk socket dimensions are suitable to classify adult females and males and show similar patterns as the results obtained from the other cranium and mandible measurements in the main text of this study. Due to the relatively small sample size of the ontogenetic groups, caution is needed prior to wide-scale application of this data on tusk sockets.

Table S14. Descriptive statistics of tusk socket measurements per ontogenetic group. All measurements are expressed in mm. Measurement abbreviations are explained in Table 2. n = number of individuals; sd = standard deviation; min = minimum value; max = maximum value.

Measurement	Ontogeny group	n	mean	sd	median	min	max	variance
**Left longitudinal**	mal_juv	1	53.42					
	fem_juv	5	37.61	5.18	38.92	29.2	43.19	26.79
	mal_adu	25	60.40	8.16	61.86	45.66	78.92	66.53
	fem_adu	23	38.92	6.25	40.24	20.94	56.78	39.06
**Right longitudinal**	mal_neo	1	35.17					
	mal_juv	1	53.13					
	fem_juv	5	37.05	4.91	37.34	29.97	43.81	24.11
	mal_adu	22	57.80	8.14	59.47	43.44	71.61	66.23
	fem_adu	21	39.14	4.91	39.11	32.79	55.21	24.11
**Left latitudinal**	fem_juv	5	22.96	1.88	22.24	21.24	25.06	3.54
	mal_adu	26	39.32	5.64	39.37	21.7	49.14	31.78
	fem_adu	22	24.28	3.02	24.70	16.07	29.27	9.14
**Right latitudinal**	fem_juv	4	22.88	3.83	22.16	19.69	27.52	14.65
	mal_adu	23	39.11	5.29	40.04	28.92	48.13	27.96
	fem_adu	21	24.80	2.40	24.39	21.45	29.91	5.77

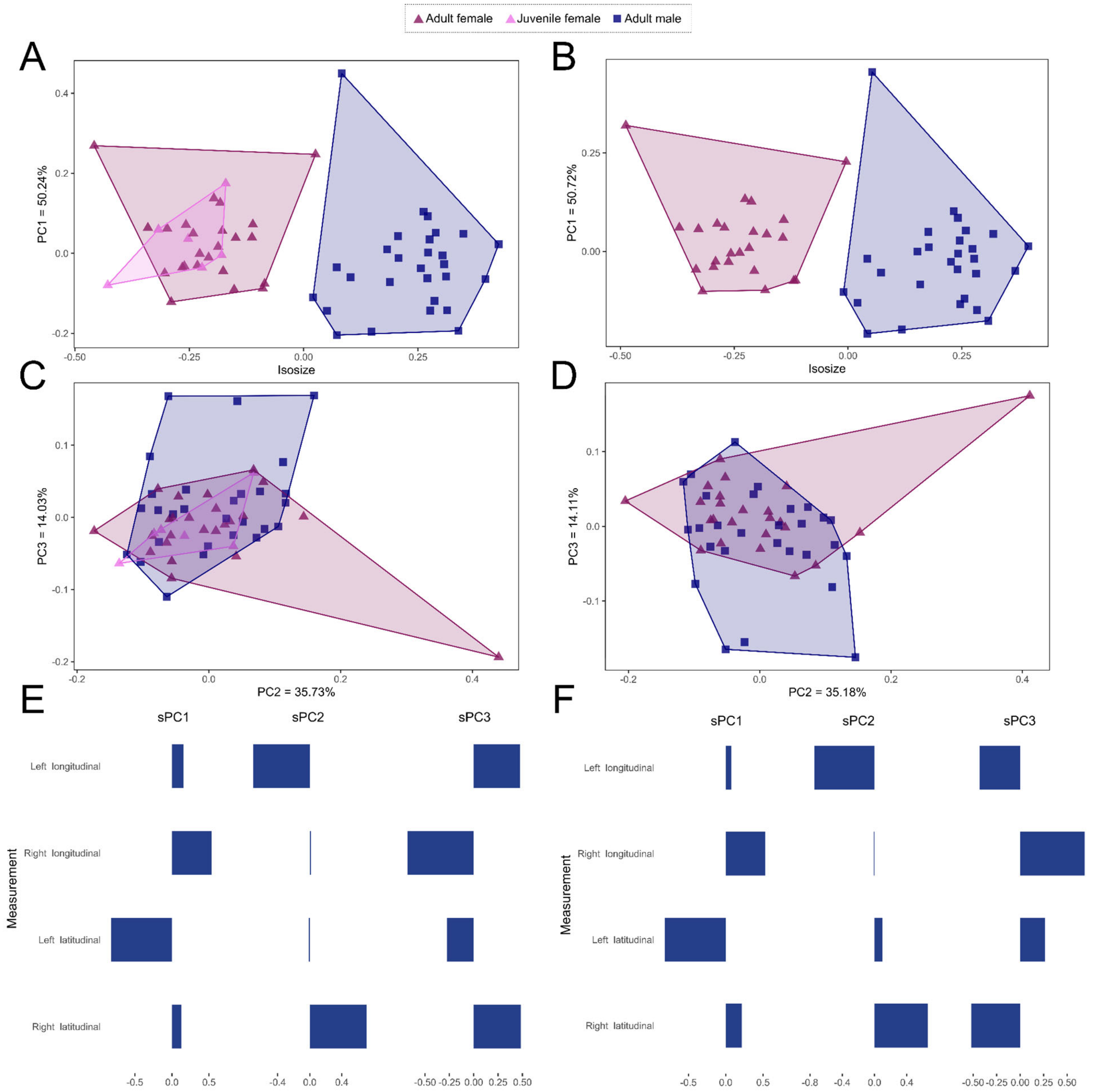

Figure S28. Principal component analysis for internal tuck sockets between age and sex groups using linear measurements. A. Isosize versus sPC1 for all included ontogenetic groups; B. Isosize versus sPC1 for adults; C. sPC2 versus sPC3 for all included ontogenetic groups; D. sPC2 versus sPC3 adults; E. loadings of the sPC for all included ontogenetic groups; F. loadings of the sPC for adults.

Table S15. Overview of sex classification results for selected specimens via linear measurements on tusk sockets compared with DNA sex attribution.

FOC	LM	Probability	DNA
3833	Male	0.9971145	Inconclusive
4489	Male	0.7928860	Male
4605	Male	0.9999761	Male
4619	Male	0.7379878	Male
4657	Male	0.6175499	Inconclusive
4845	Male	1.0000000	Male
5061	Male	0.9991235	Male

Ancient DNA Sexing

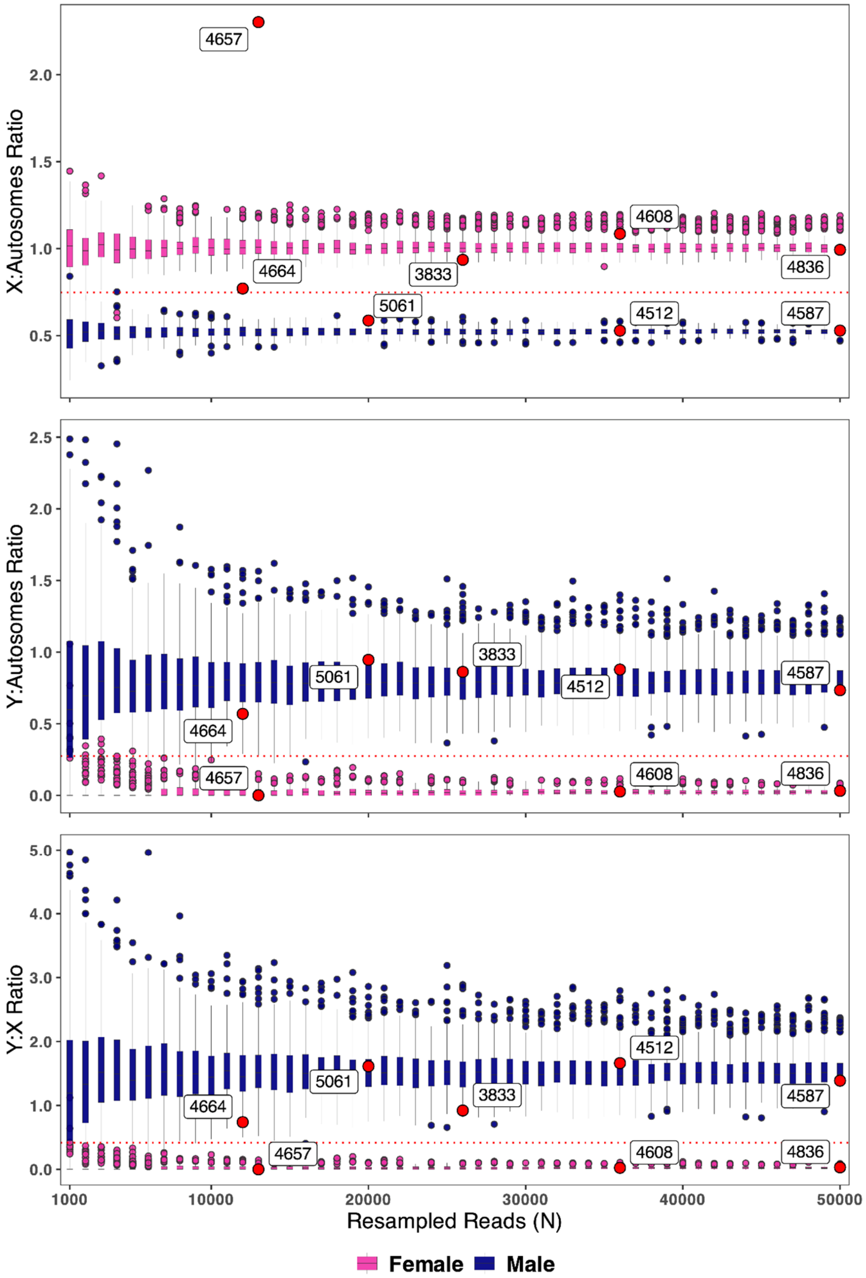

Figure S29. Genomic sexing of Atlantic Walrus specimens using a downsampling approach of ancient DNA sequencing data. Ratio of X chromosome to median autosome coverage (top), Y chromosome to median autosome coverage (middle) and Y chromosome to X chromosome coverage (bottom) generated by randomly subsampling BAM files of a reference sample dataset (N=21) in increments of 1,000 reads to contain a final number of mapped reads ranging from 1,000 - 50,000. At each interval, and for each individual, the downsampling was iterated 20 times. The red, horizontal dotted line depicts a cutoff to facilitate sex determination. Eight individuals (red dots) did not have sufficient reads (>100,000 mapped reads) to conduct a robust downsampling approach. Their ratios were calculated from original, complete BAM files.

Table S23. Sequencing and mapping characteristics of the 30 Atlantic walrus samples used for genomic sexing.

FOC specimen	Internal sample ID	Assigned genomic sex	No. of total raw reads	PCR duplication	Endogeny	No. of unique mapped reads (w/o duplicates)	Average read length (bp)	Average depth of coverage
3018	WLR081	Male	26 524 292	6.58 %	30.02 %	3 923 032	76.56	0.1158
3771	WLR105	Female	27 432 398	12.97 %	34.72 %	3 630 099	79.41	0.0946
3828	WLR096	Male	12 565 870	1.74 %	53.22 %	3 178 570	59.90	0.0777
3833	WLR097	Inconclusive	25 222 338	44.33 %	0.23 %	25 534	85.74	0.0007
4332	WLR107	Male	27 653 888	4.28 %	5.12 %	655 142	65.31	0.0146
4419	WLR108	Male	10 455 244	2.20 %	18.91 %	964 913	83.64	0.0355
4489	WLR109	Male	18 424 546	5.62 %	24.51 %	1 848 099	83.44	0.0502
4496	WLR110	Male	10 345 128	5.25 %	13.60 %	677 116	88.14	0.0220
4510	WLR111	Female	20 030 770	2.48 %	54.16 %	5 161 770	68.24	0.1424
4512	WLR112	Male	5 961 298	8.12 %	1.37 %	36 454	61.39	0.0009
4538	WLR113	Male	13 018 798	4.55 %	9.12 %	538 343	86.47	0.0152
4543	WLR114	Male	15 402 726	12.57 %	10.20 %	651 413	77.61	0.0184
4587	WLR115	Male	8 285 304	7.85 %	2.41 %	89 617	64.95	0.0023
4597	WLR116	Female	11 926 856	2.24 %	68.98 %	4 031 328	80.15	0.1306
4600	WLR117	Male	16 644 356	3.50 %	4.54 %	364 744	86.73	0.0100
4605	WLR098	Male	34 905 086	2.13 %	74.48 %	12 503 306	65.84	0.3211
4608	WLR118	Female	14 400 372	51.11 %	0.56 %	36 426	67.26	0.0012
4616	WLR099	Female	15 675 960	2.06 %	37.17 %	2 442 759	63.80	0.0629
4617	WLR100	Female	25 189 966	2.71 %	61.13 %	7 342 215	60.66	0.1825
4619	WLR119	Male	10 812 348	6.03 %	41.72 %	1 750 884	68.83	0.0452
4657	WLR101	Inconclusive	10 056 614	79.33 %	0.32 %	12 988	69.73	0.0005
4663	WLR120	Male	10 318 730	4.10 %	15.35 %	693 754	72.30	0.0195
4664	WLR121	Inconclusive	11 106 344	39.63 %	0.23 %	11 868	76.88	0.0004
4668	WLR122	Female	16 248 974	4.74 %	3.50 %	269 465	81.52	0.0083
4669	WLR102	Male	17 762 208	2.39 %	70.30 %	5 948 197	63.97	0.1580
4819	WLR103	Female	33 478 852	5.81 %	1.57 %	254 308	62.76	0.0062

4836	WLR123	Female	13 791 684	20.63 %	1.46 %	80 647	76.80	0.0022
4841	WLR124	Female	13 883 844	5.47 %	11.43 %	661 942	82.77	0.0198
4845	WLR104	Male	19 903 694	2.71 %	49.74 %	4 513 366	63.38	0.1114
5061	WLR125	Male	10 133 604	27.68 %	0.44 %	20 218	67.80	0.0005

Recommendations on how to use the classification script

An Rscript (SI) is provided to facilitate the classification and sexing of unknown specimens for modern, historical and archaeological material using linear measurement and geometric morphometrics. As the LDA requires a reference set of specimens with known age and sex, the dataset for the ontogenetic description provided in this study can be used, but other reference data can also be loaded. Similarly, the csv file in SI contains the information to create a subset of unknown specimens to classify (column "ontogenetic study", classifier "test …"), and the specimens in this file can be replaced by others. The test specimens are split between adults and juveniles and marked as such to facilitate the classification and increase the success rate, as the ages were determined via suture fusion states. While the protocol in the provided script only includes measurements or landmarks present in the specimens being identified for the analysis, it is still recommended to apply this method on specimens with sufficient morphometric data (i.e. one linear measurement or minimally three landmarks should be present for morphometric classification and more is better). Where possible, applying both linear and geometric methods are advised, as well as applying the LDA on both crania and mandibles, to increase confidence in the result.
