# Supplementary figures and images for: Ontogenetic changes and sexual dimorphism in the cranium and mandible of the Atlantic walrus (Odobenus rosmarus rosmarus L.)
Source: Anat Rec (Hoboken). 2025 Sep 12;309(7):1875–903. doi: 10.1002/ar.70050 (PMC13251751; doi:10.1002/ar.70050)

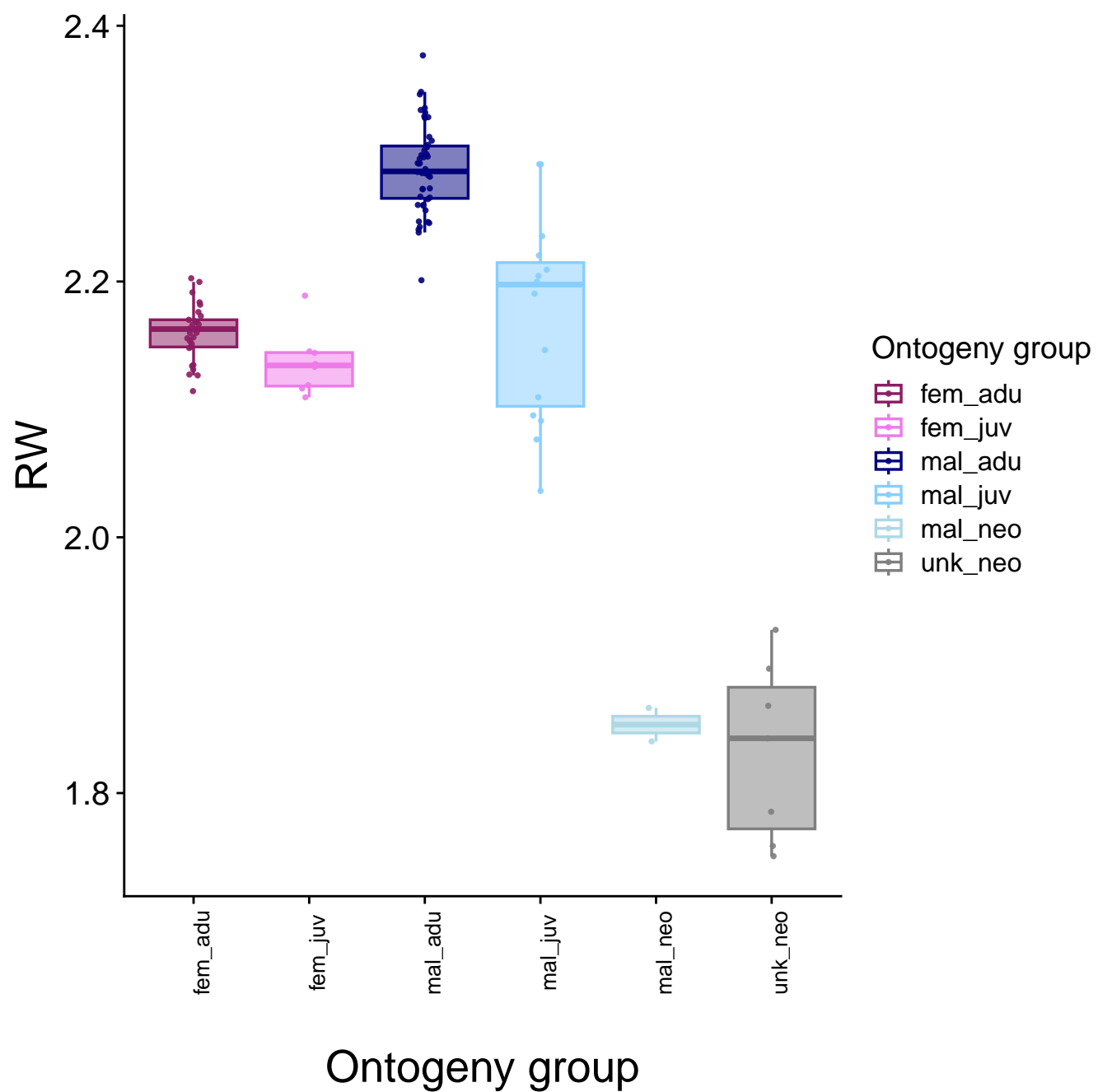

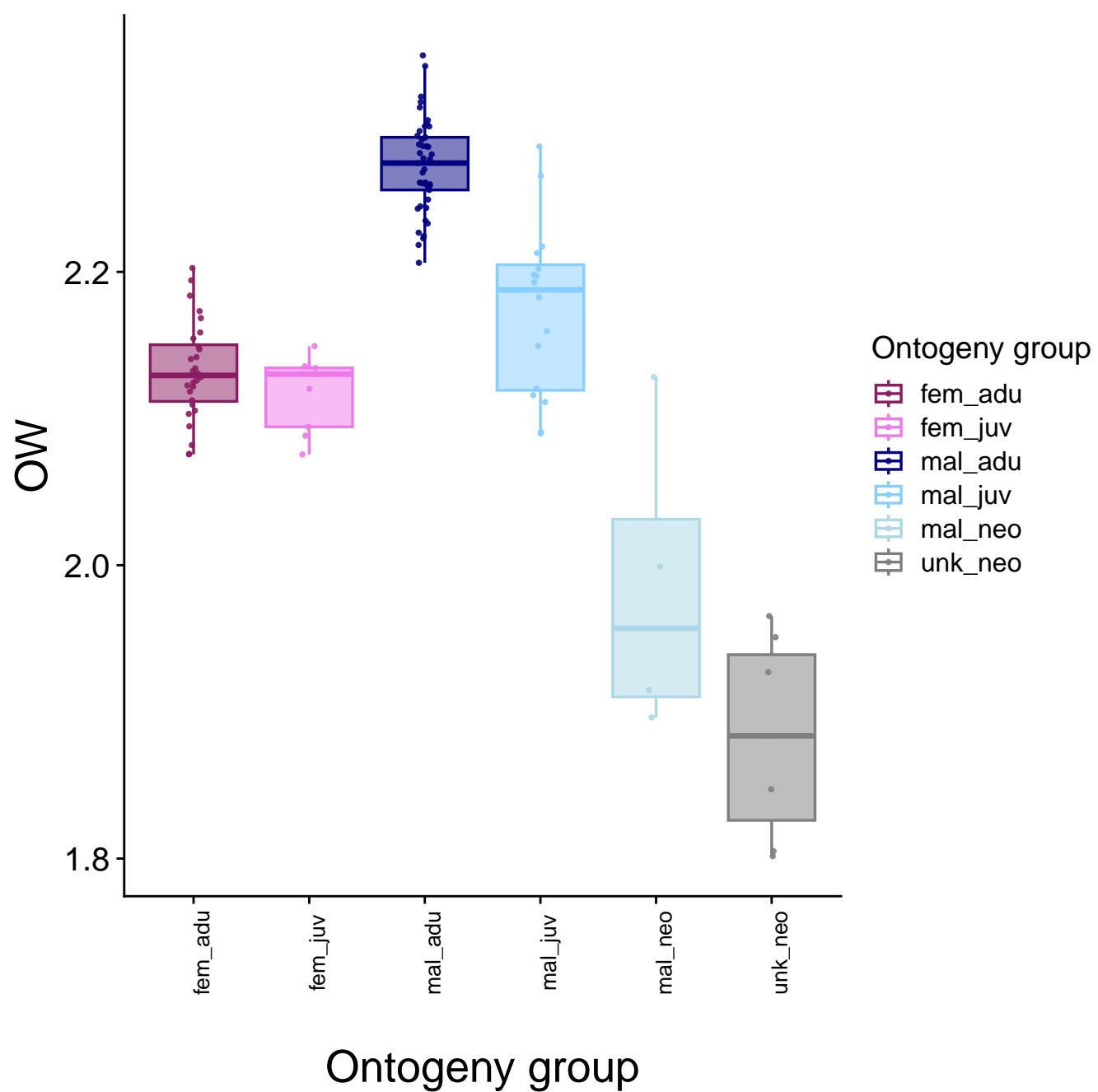

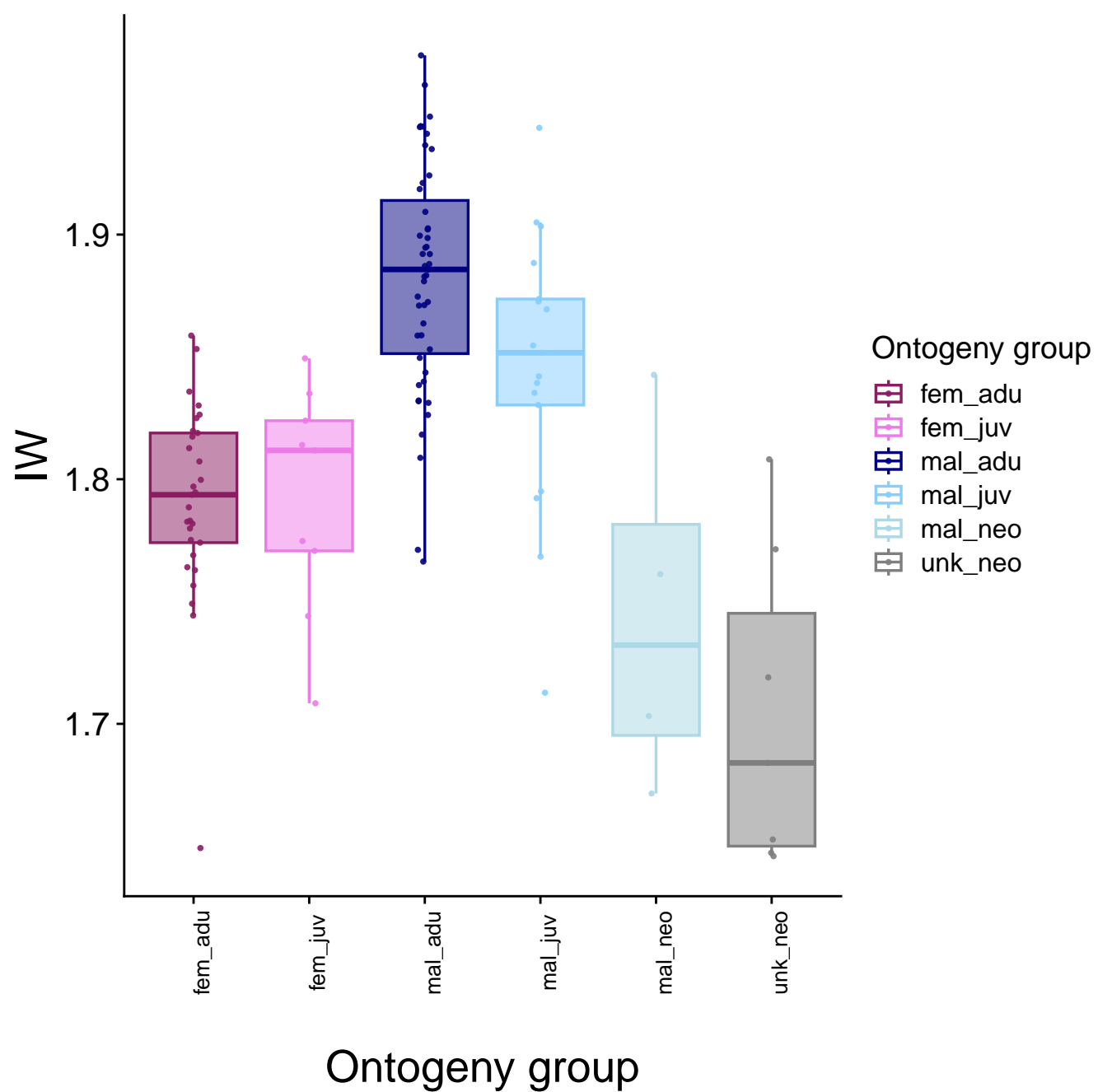

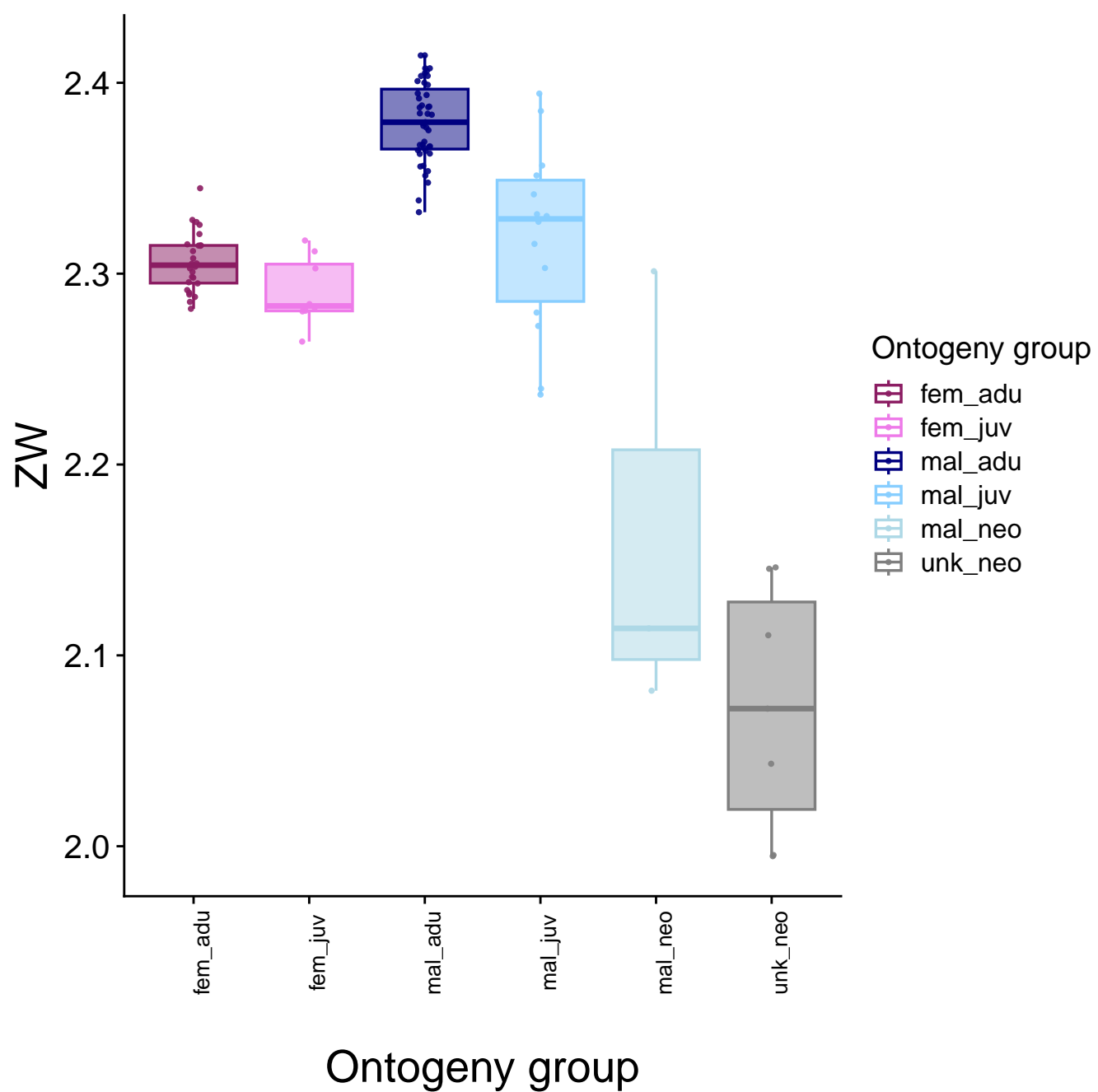

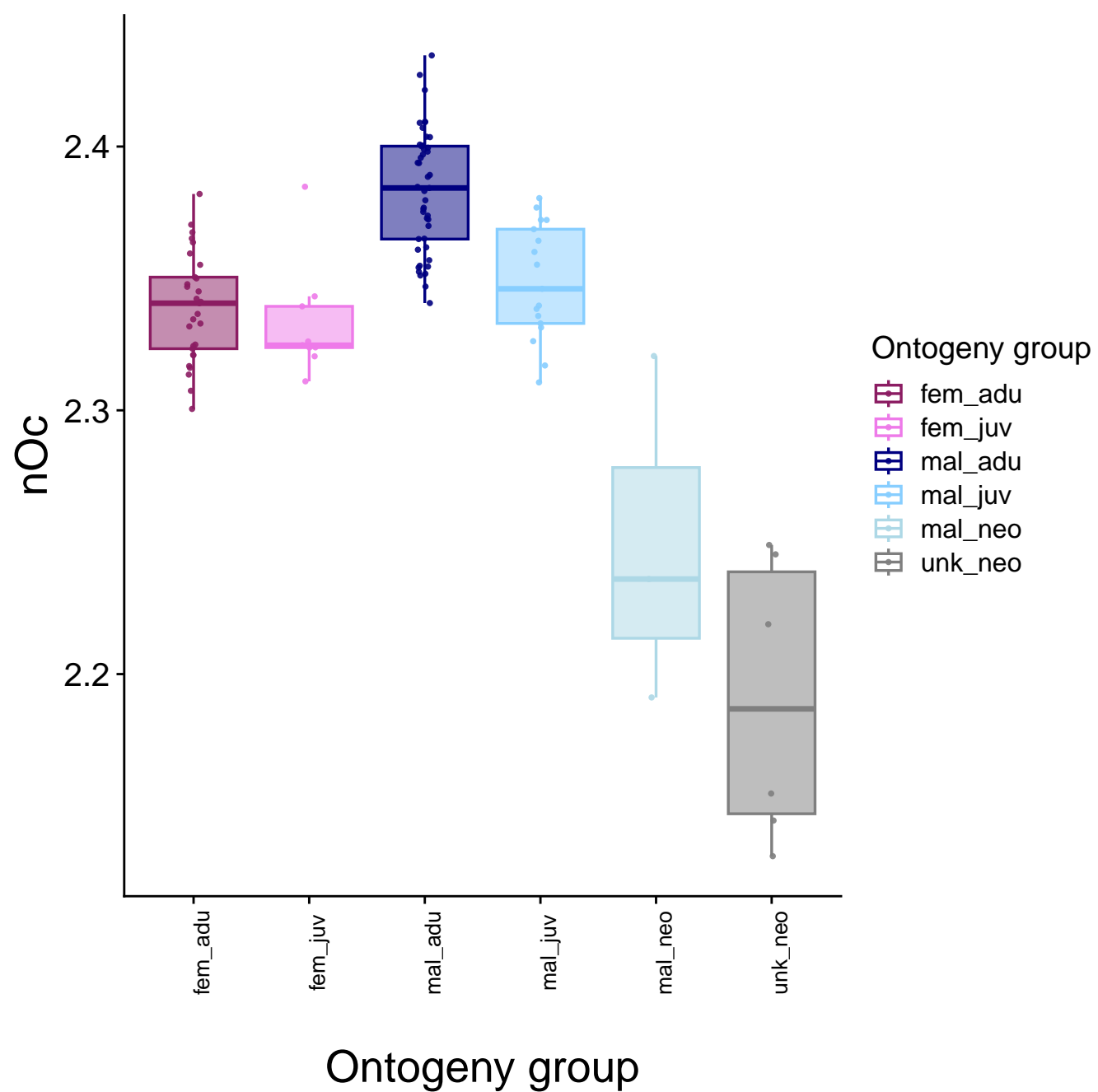

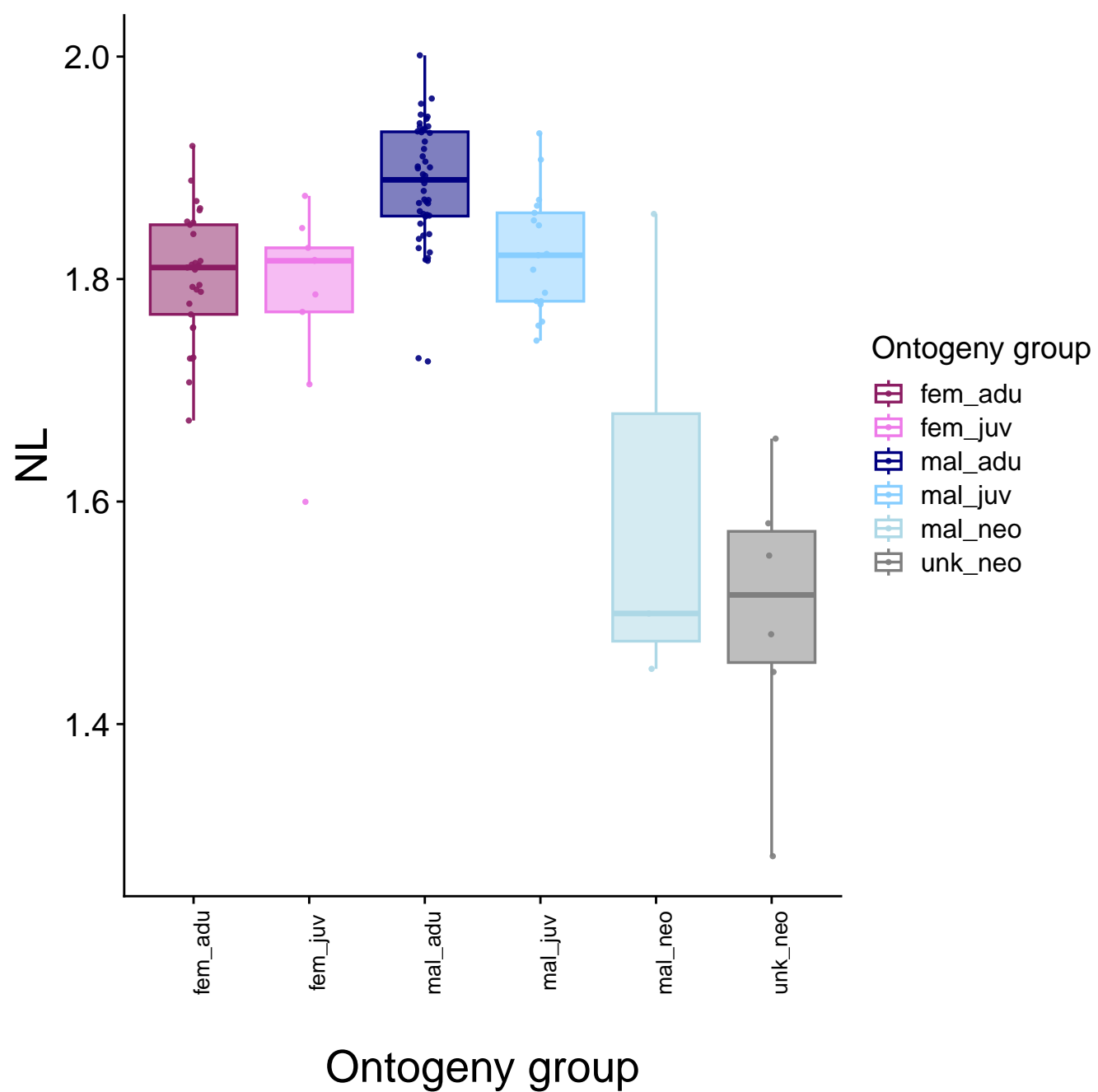

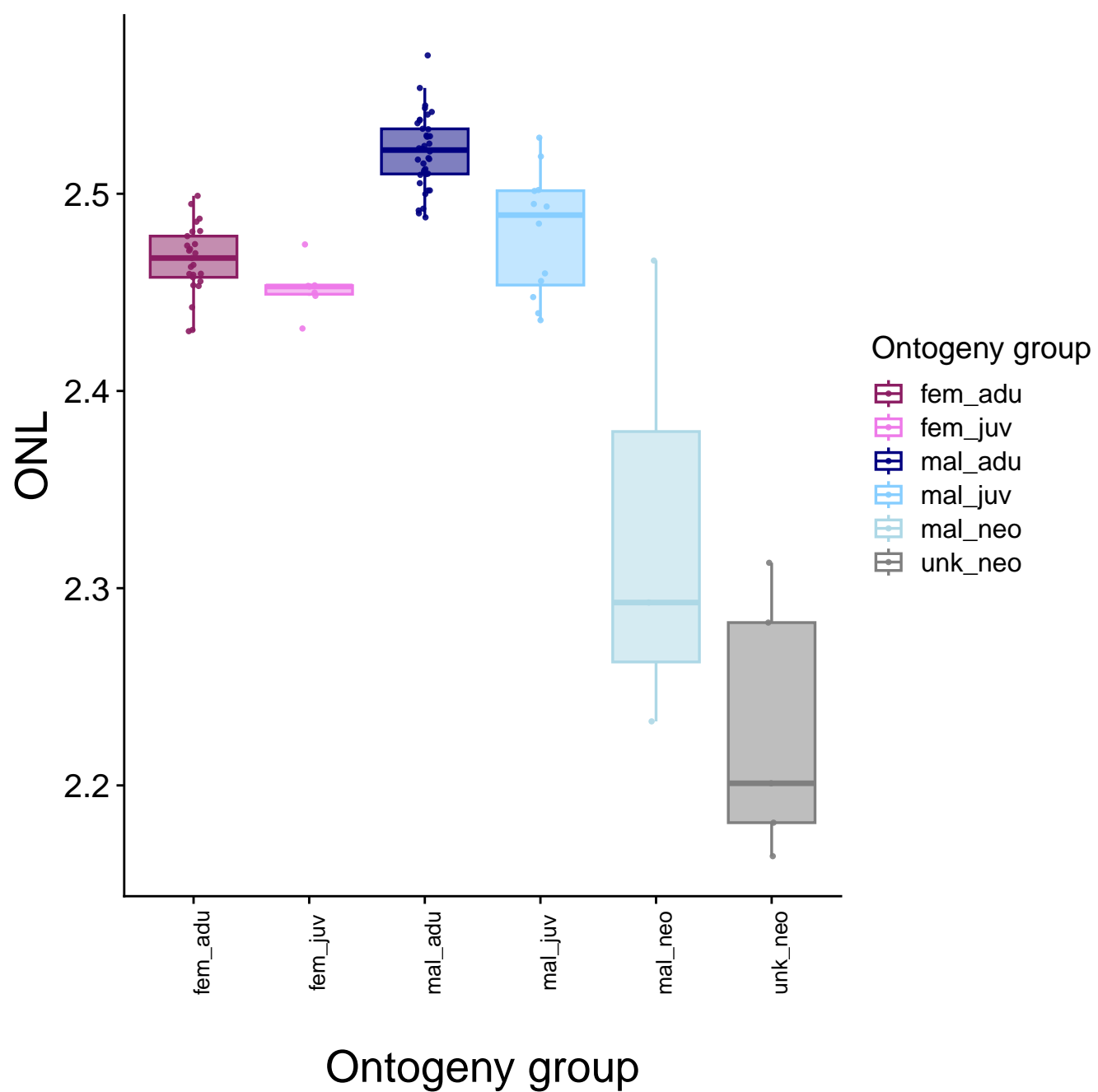

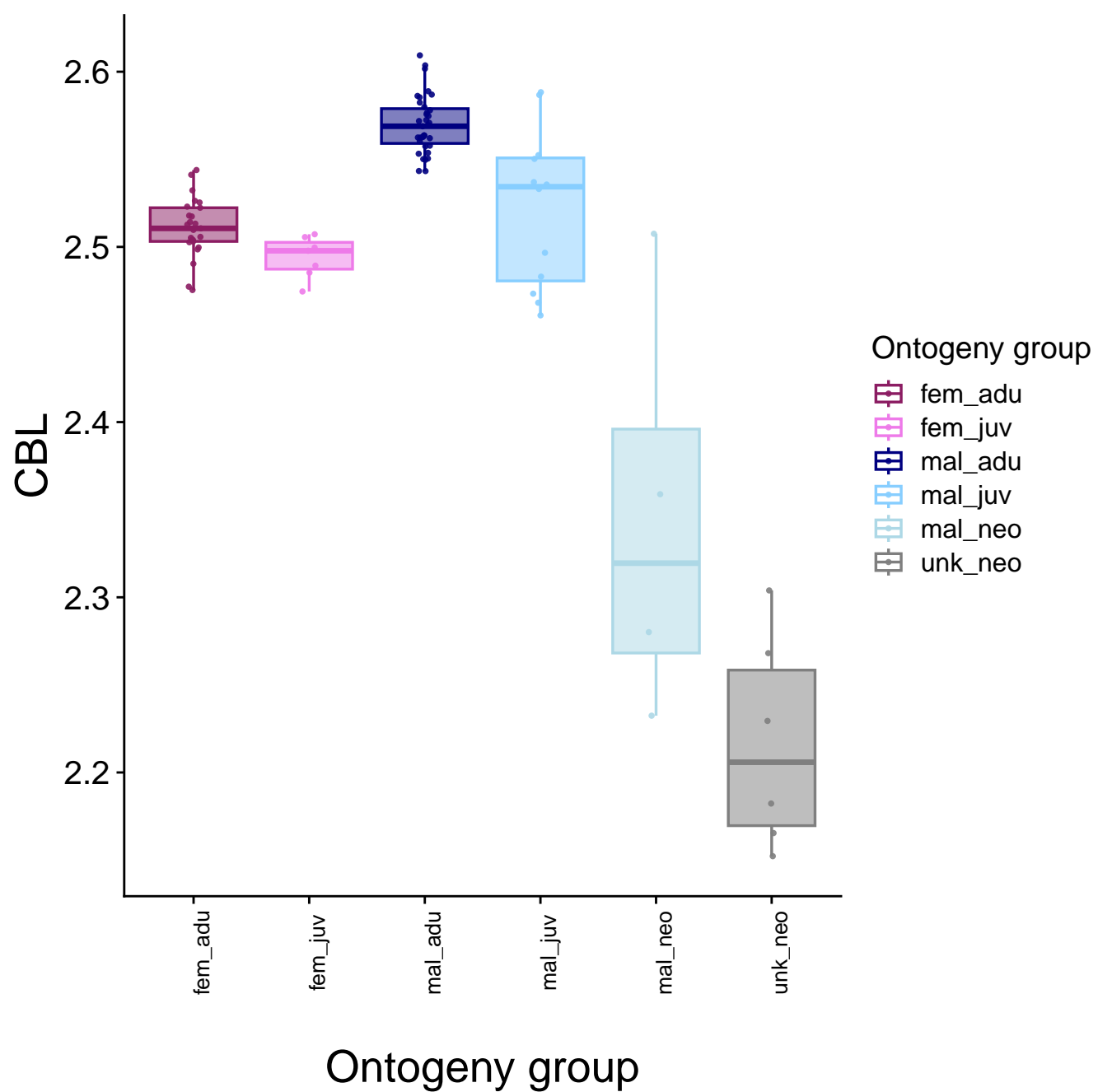

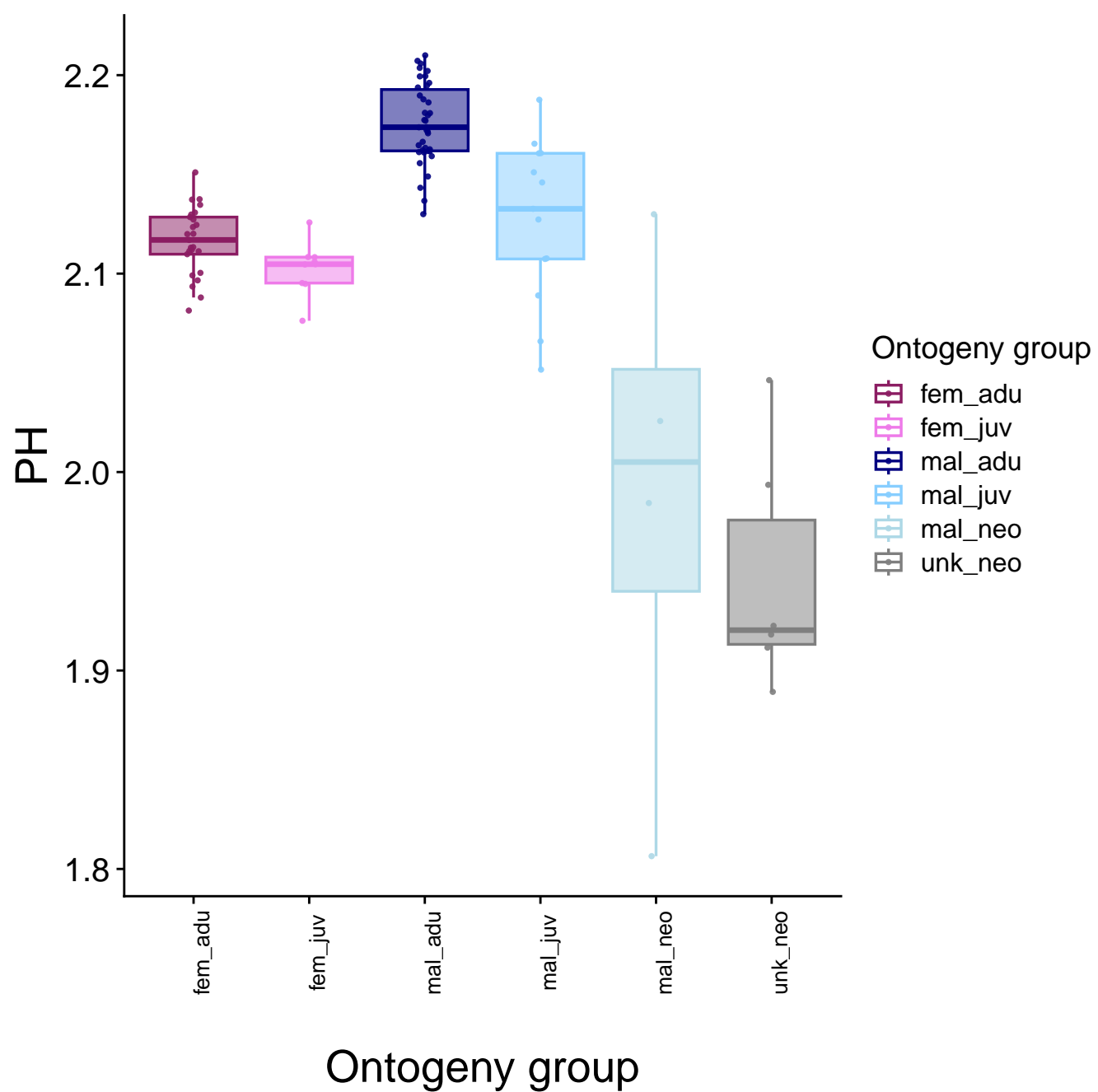

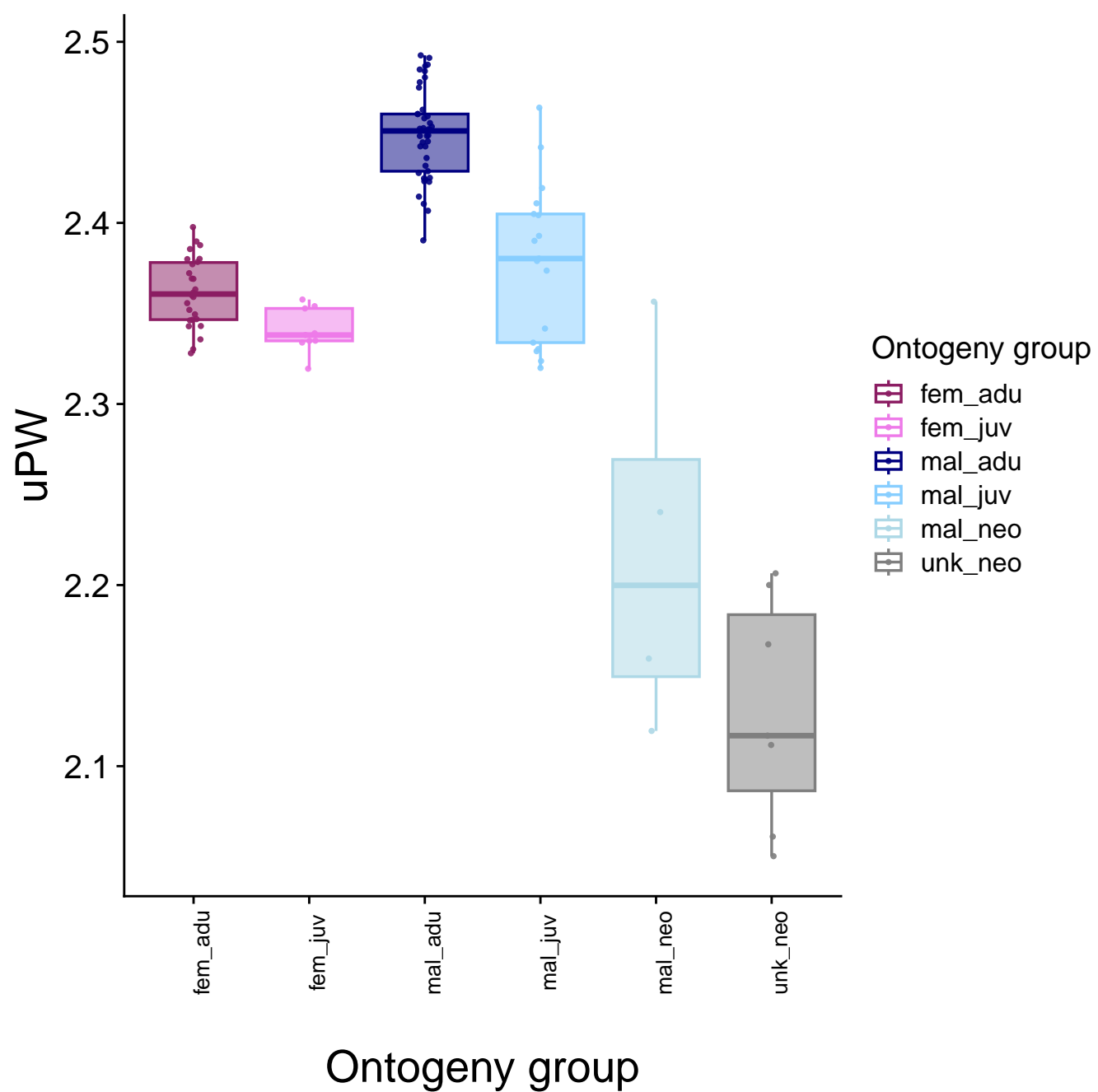

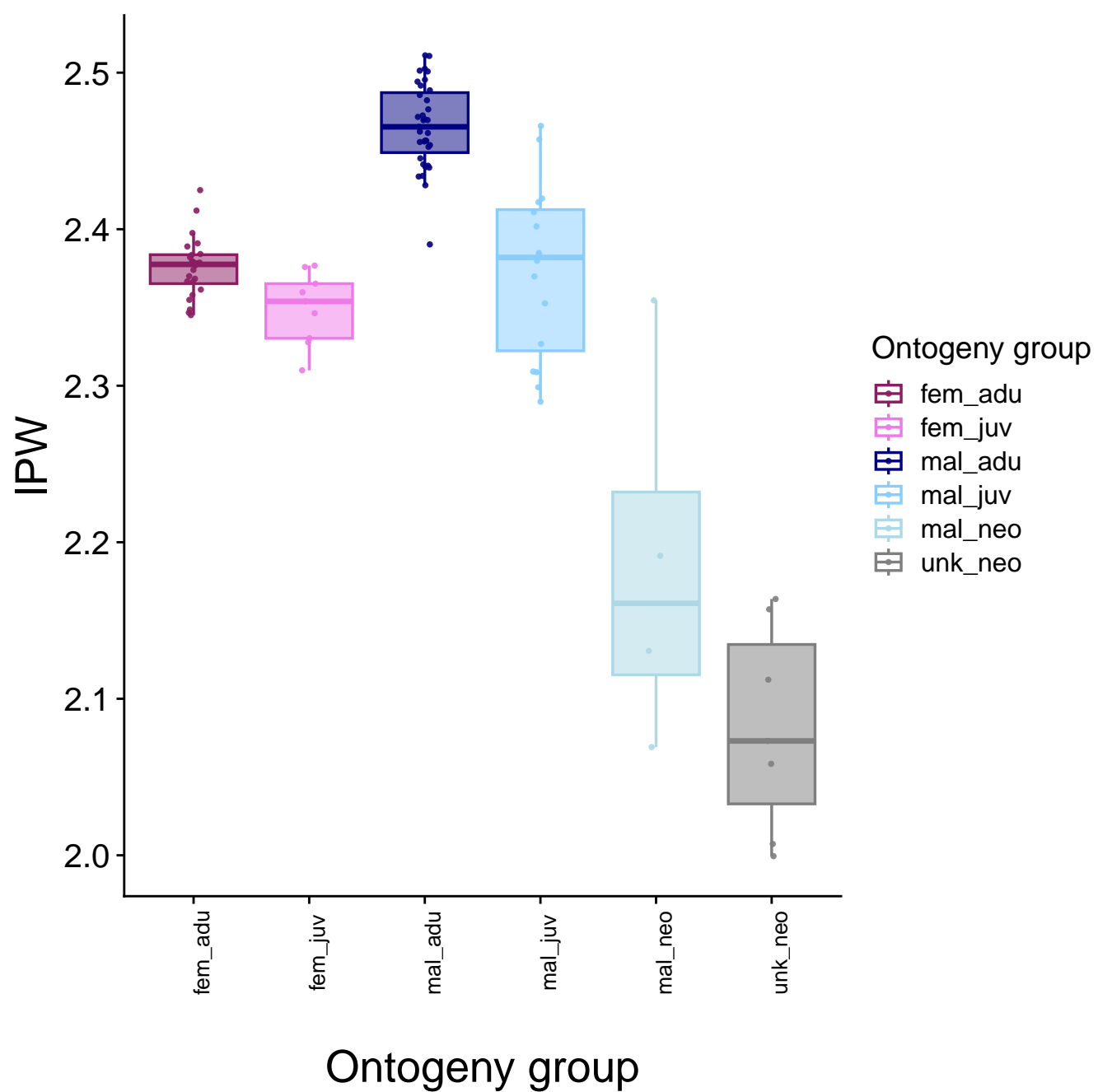

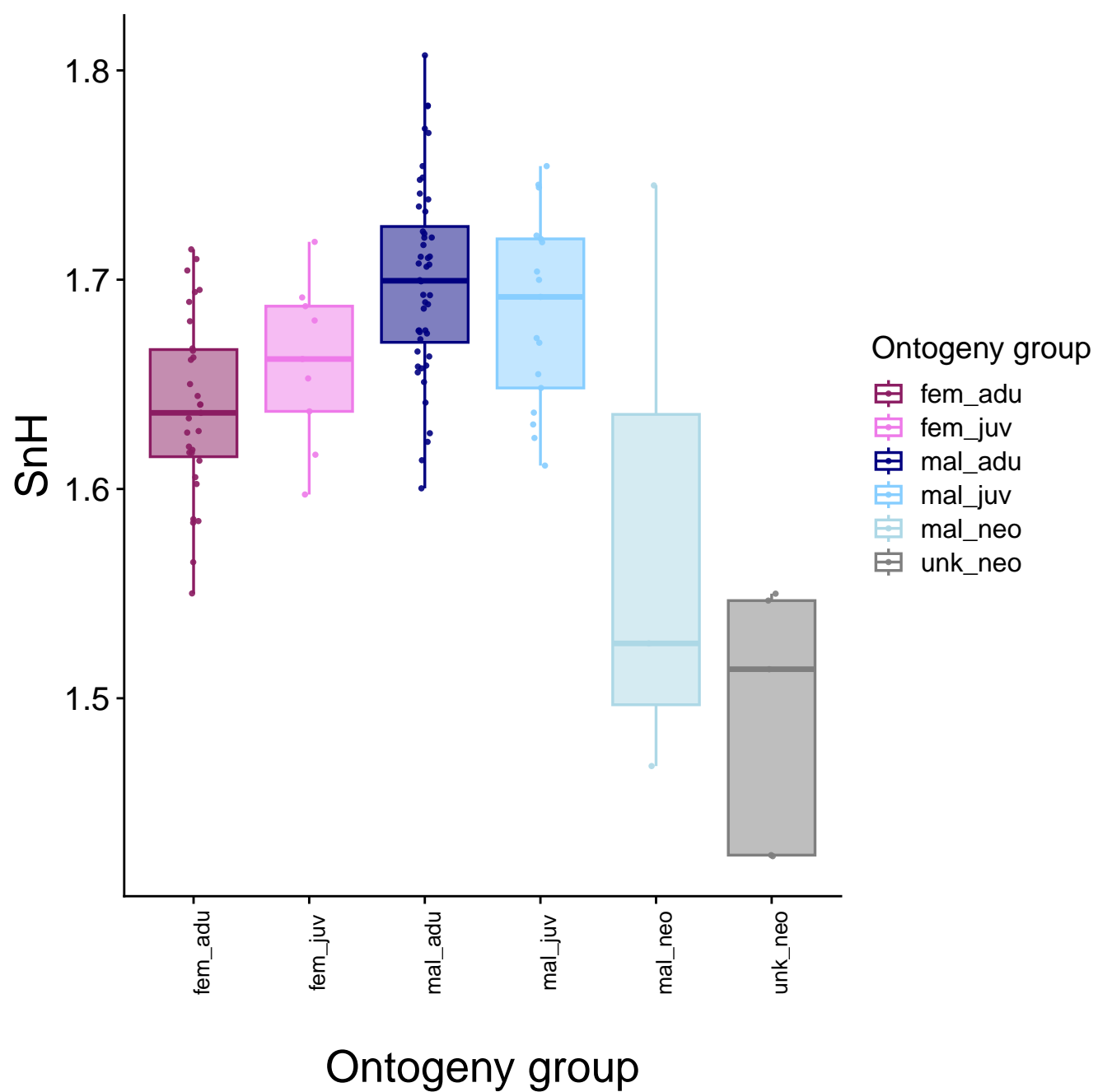

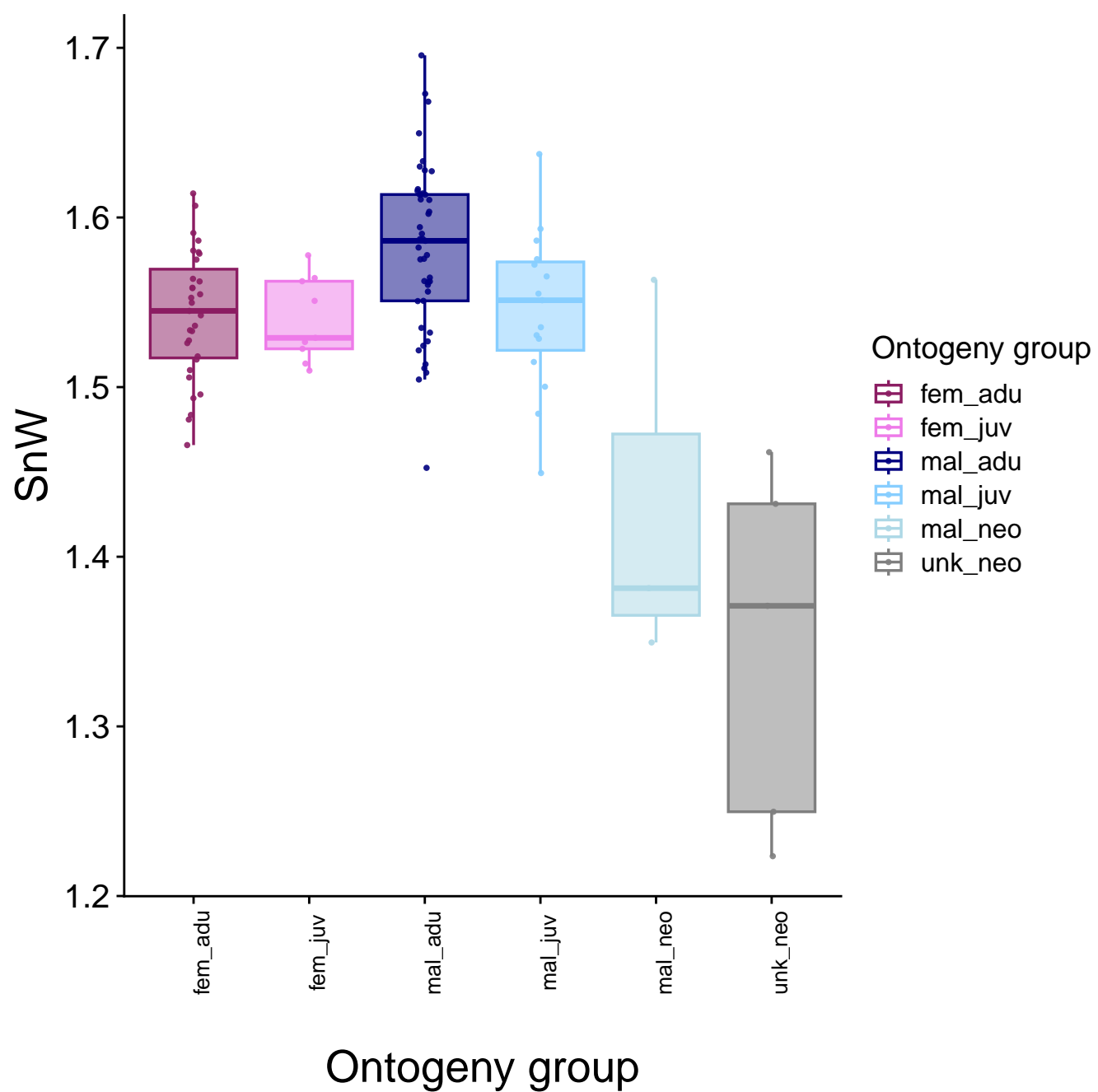

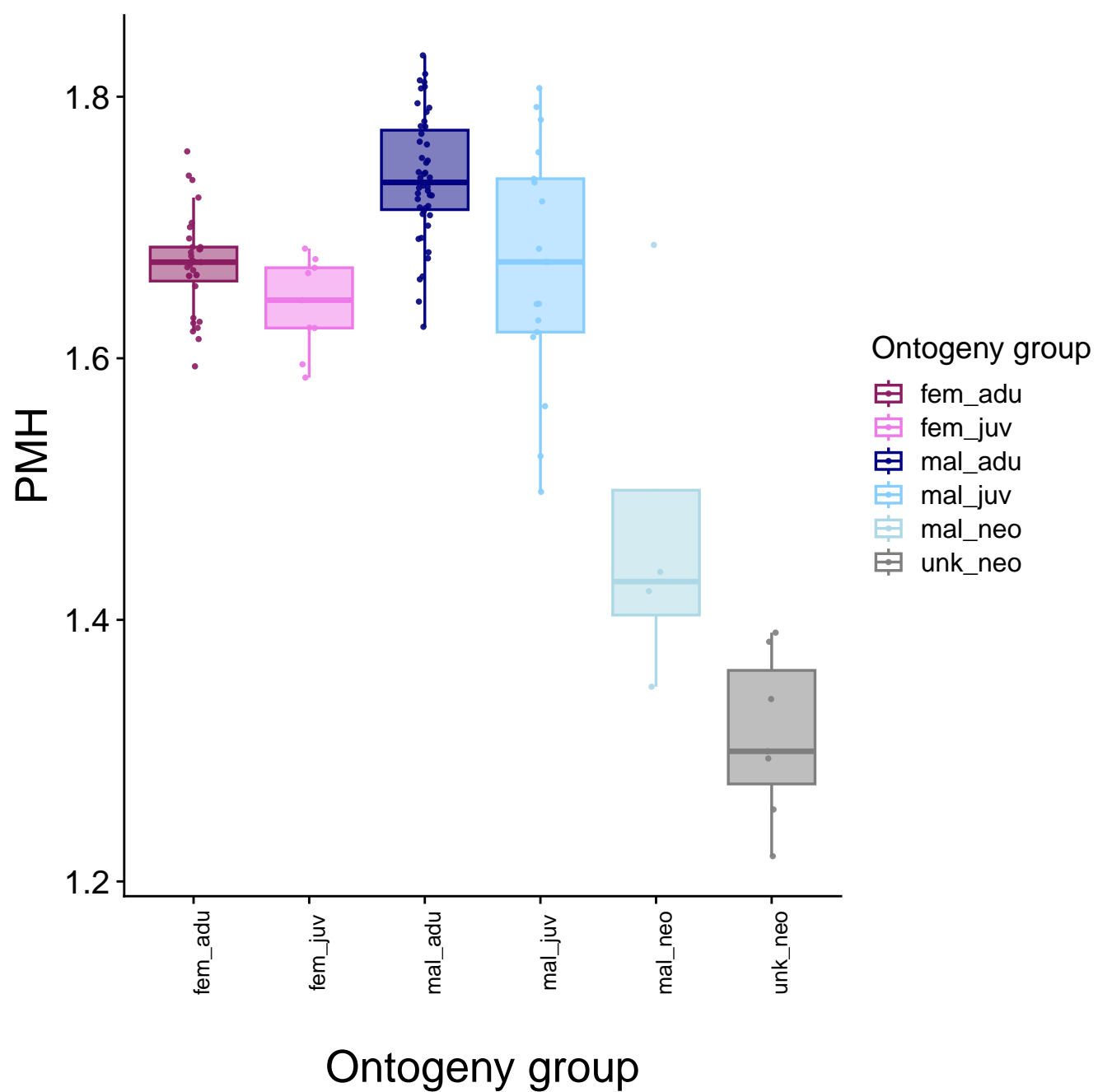

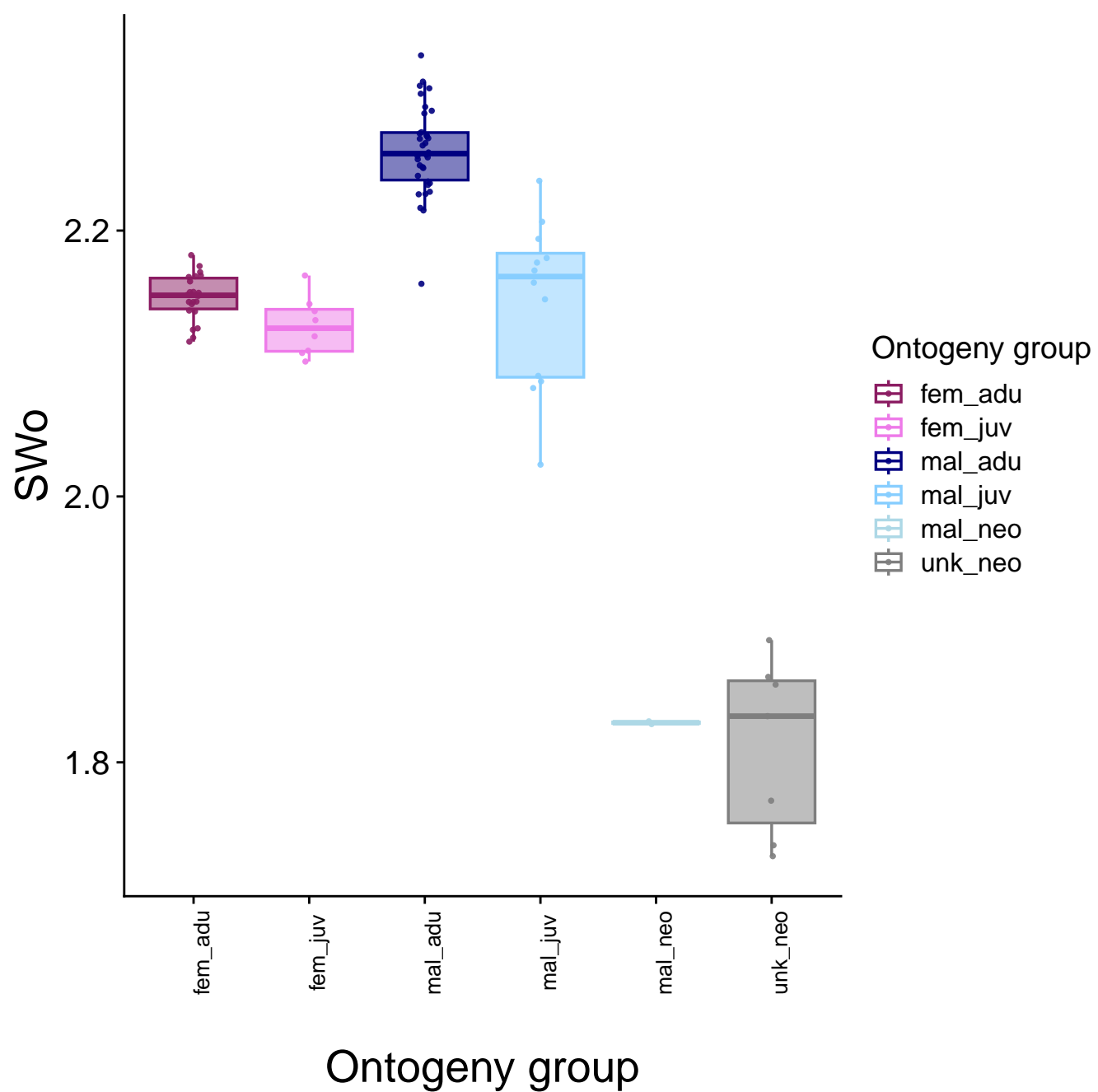

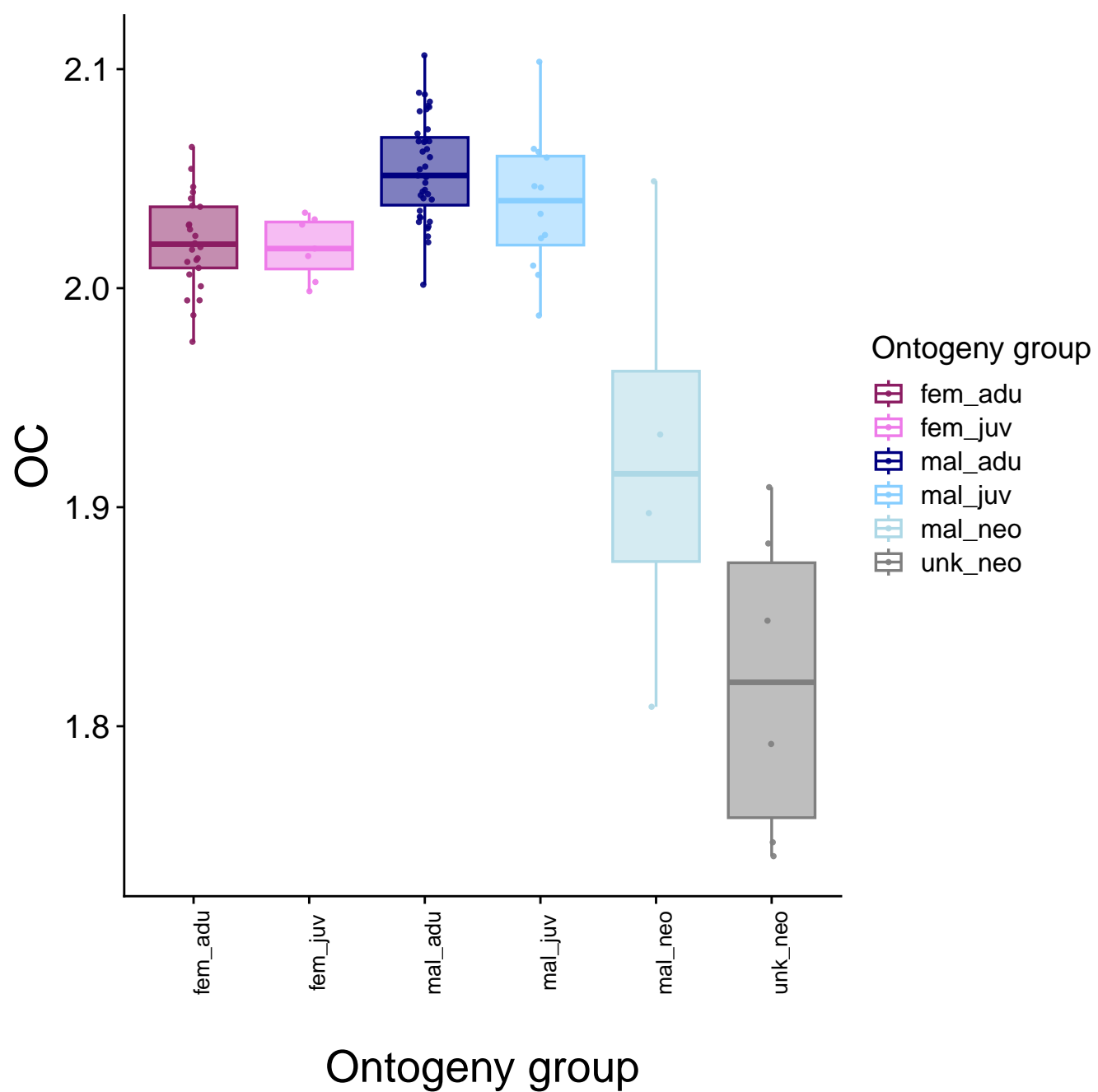

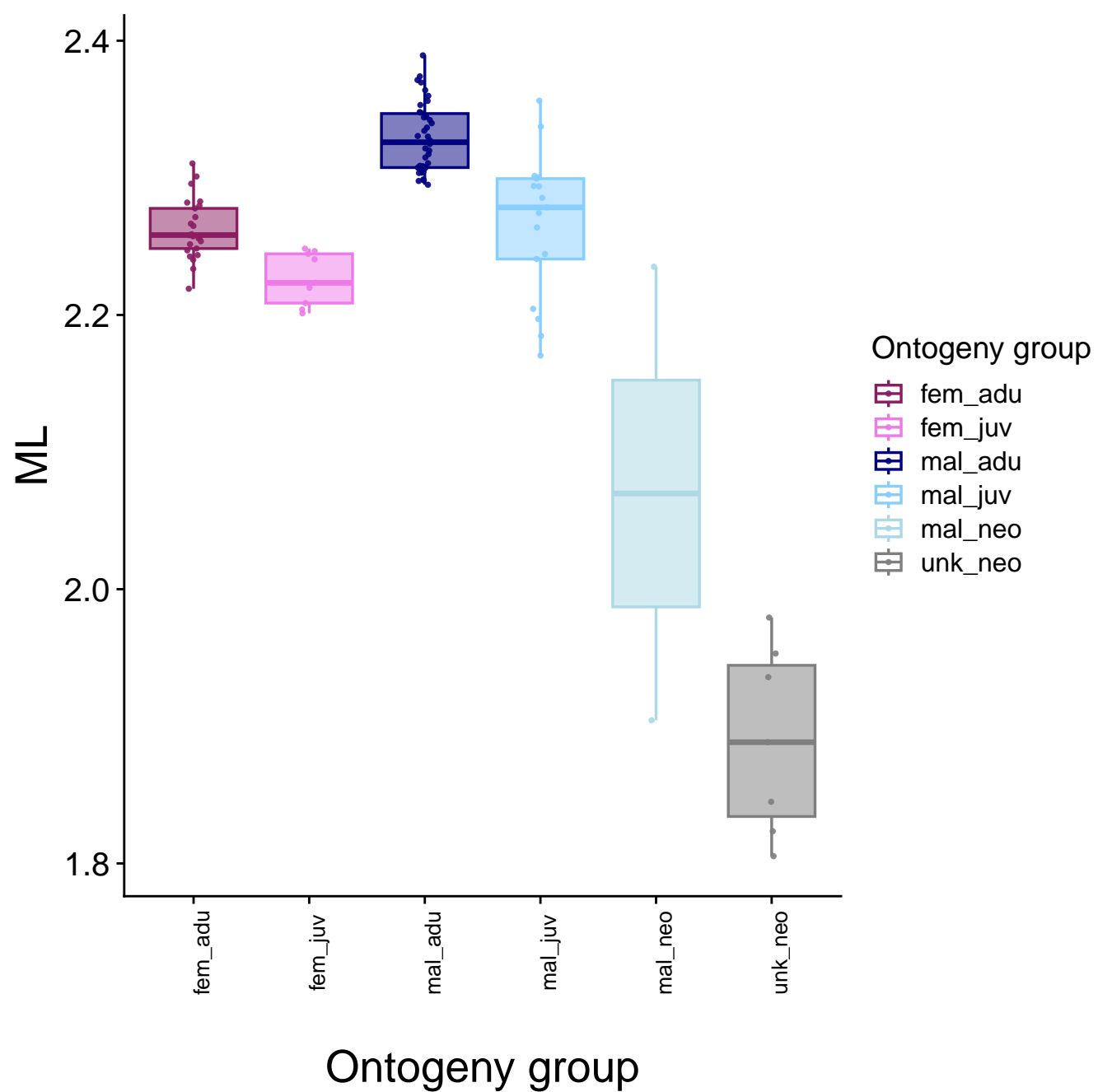

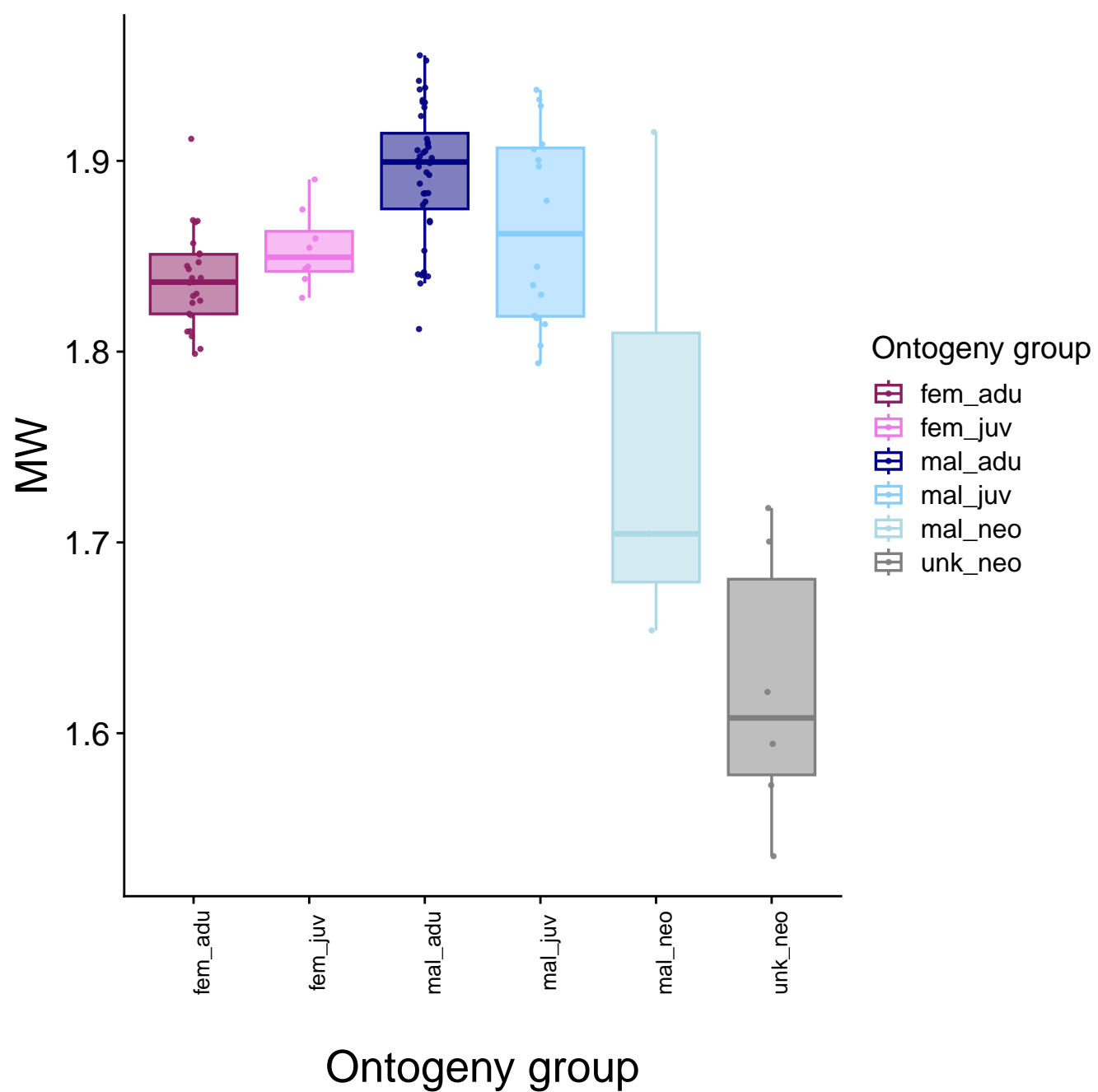

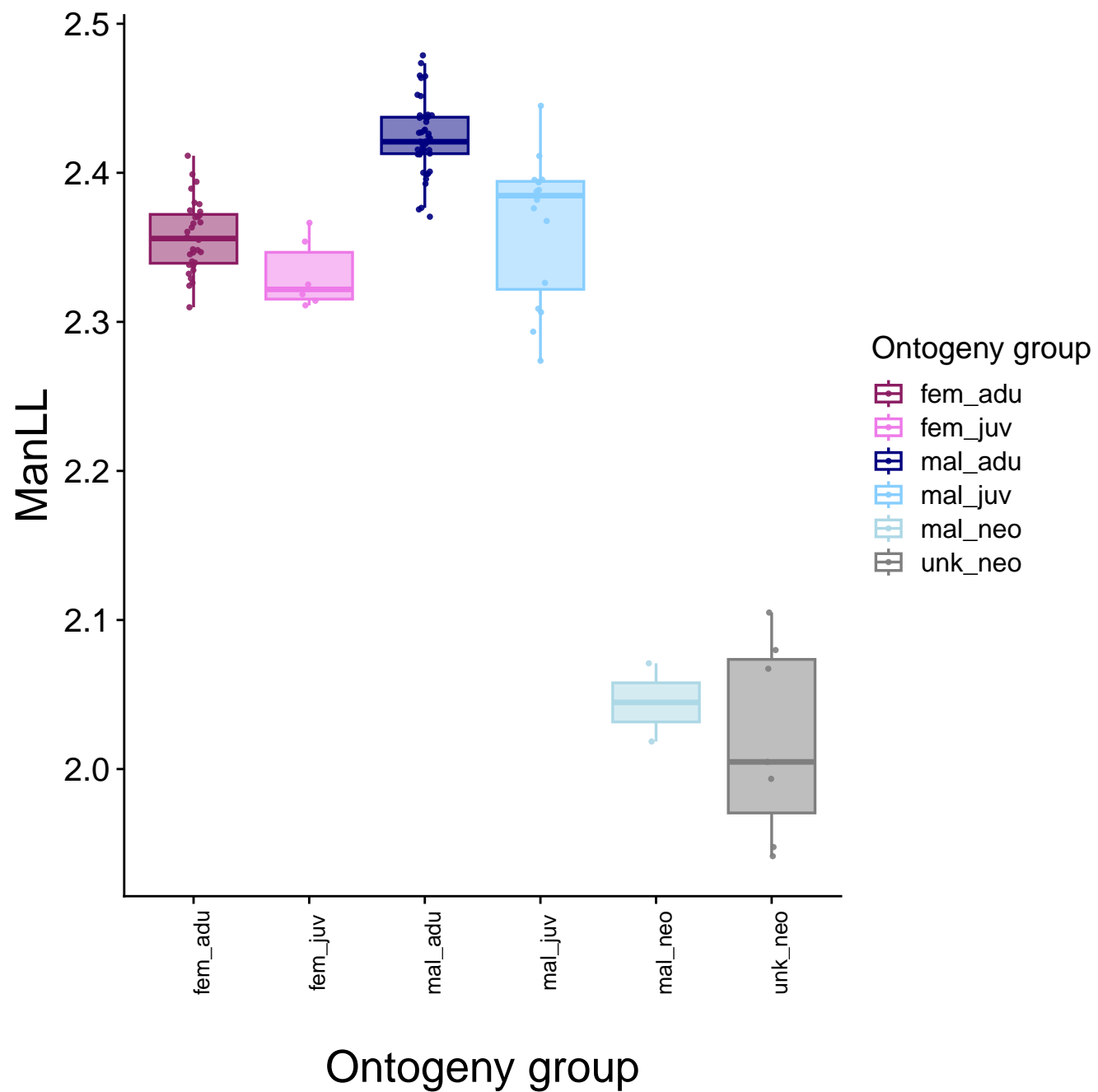

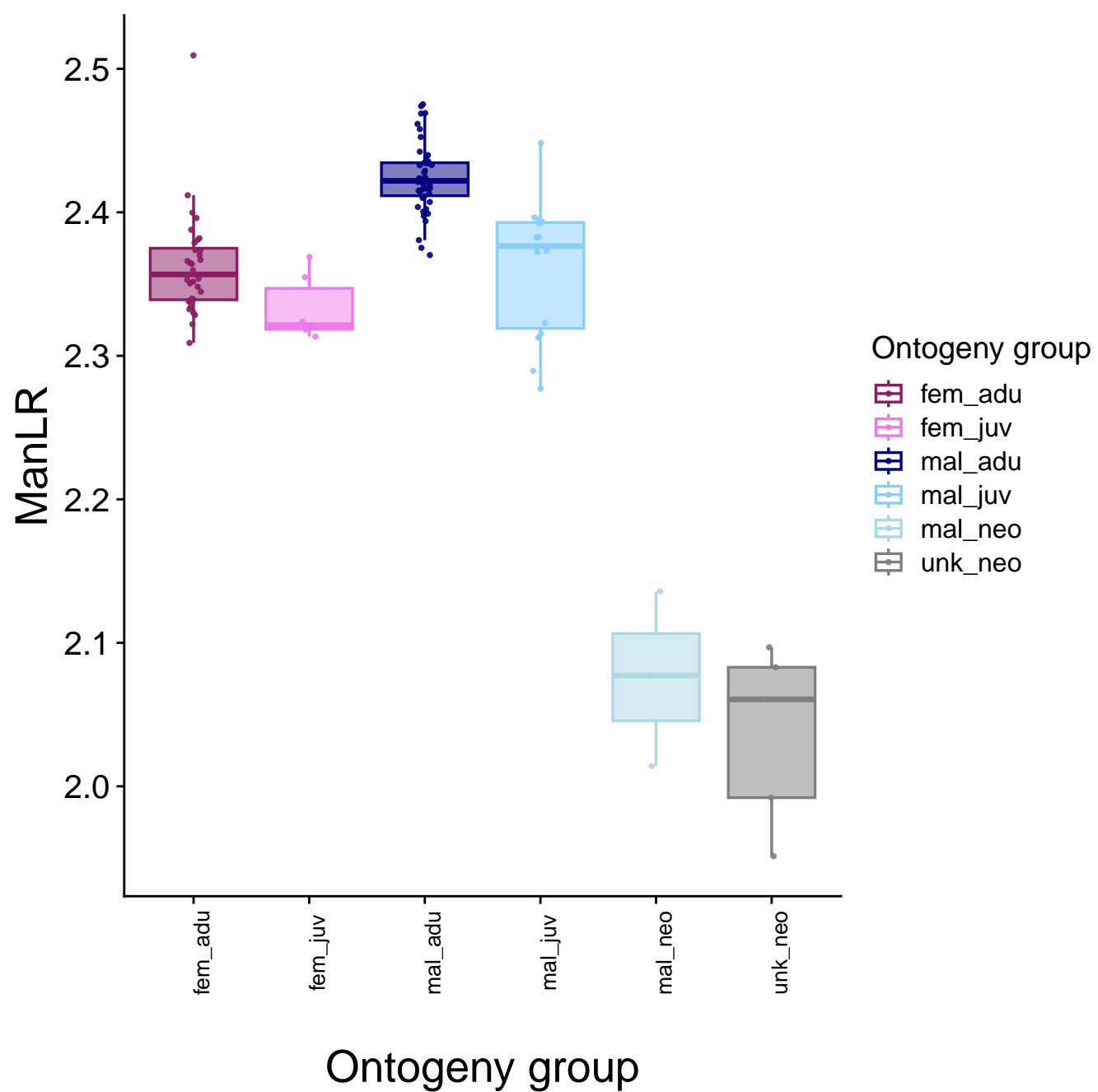

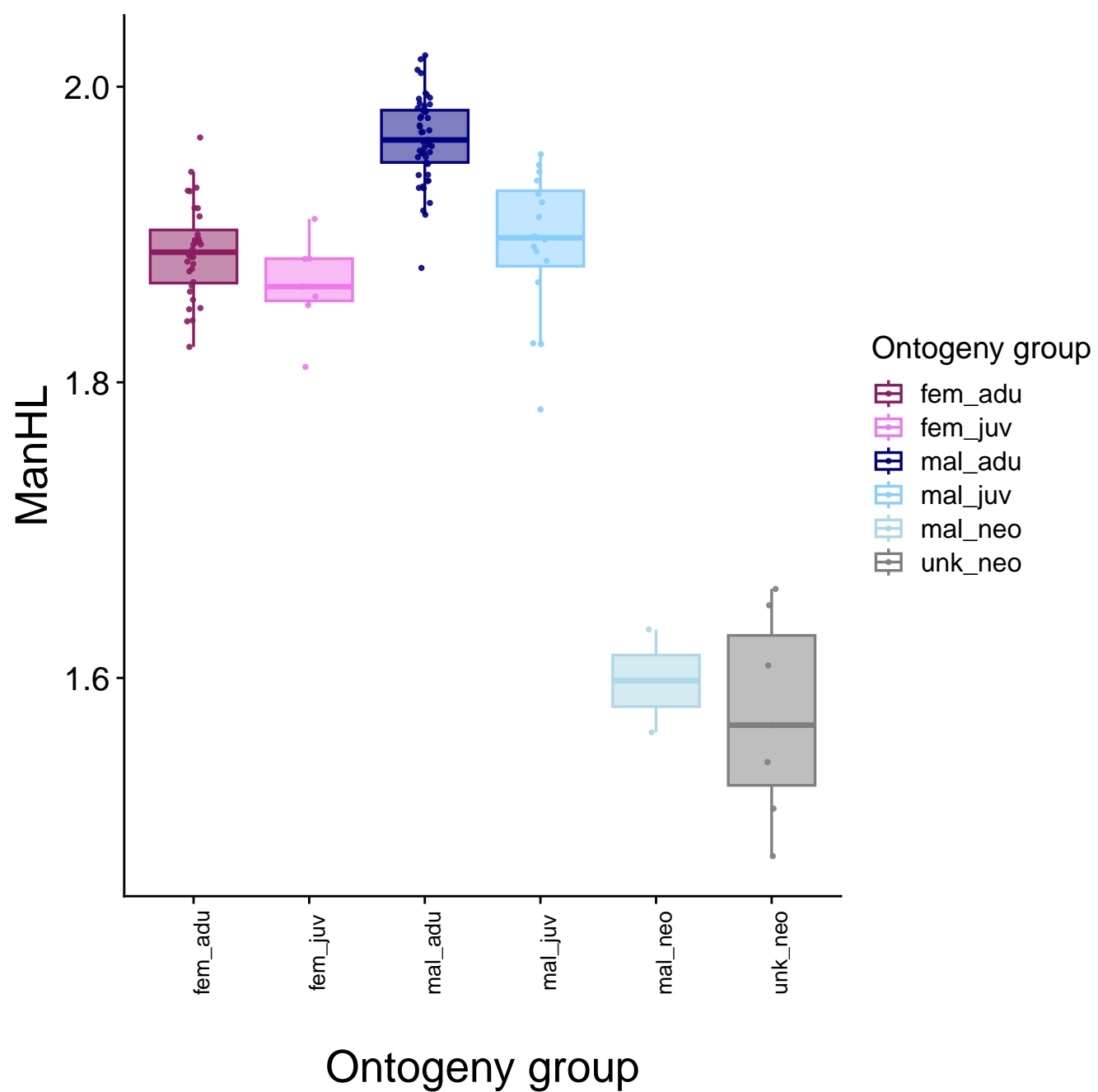

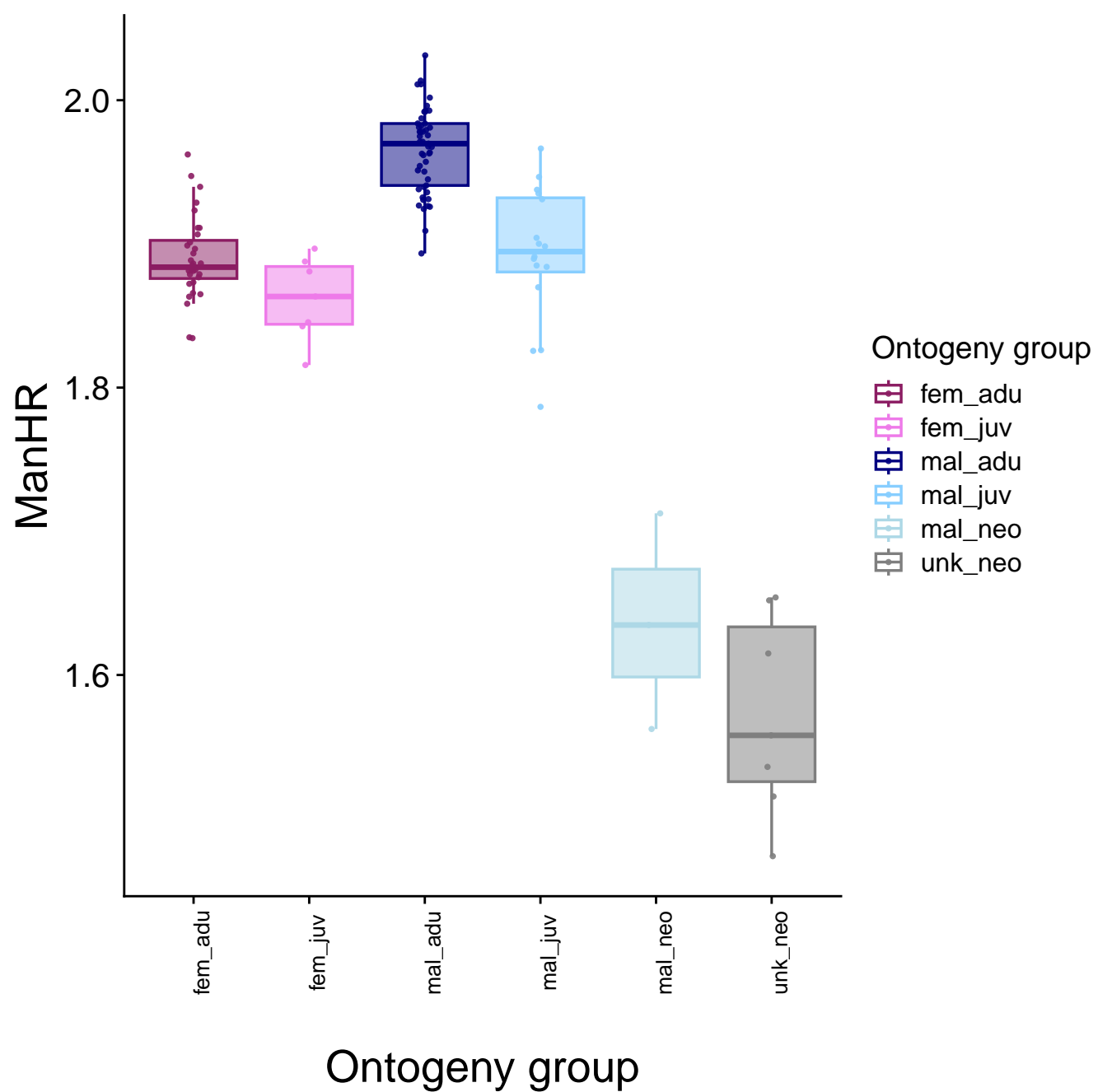

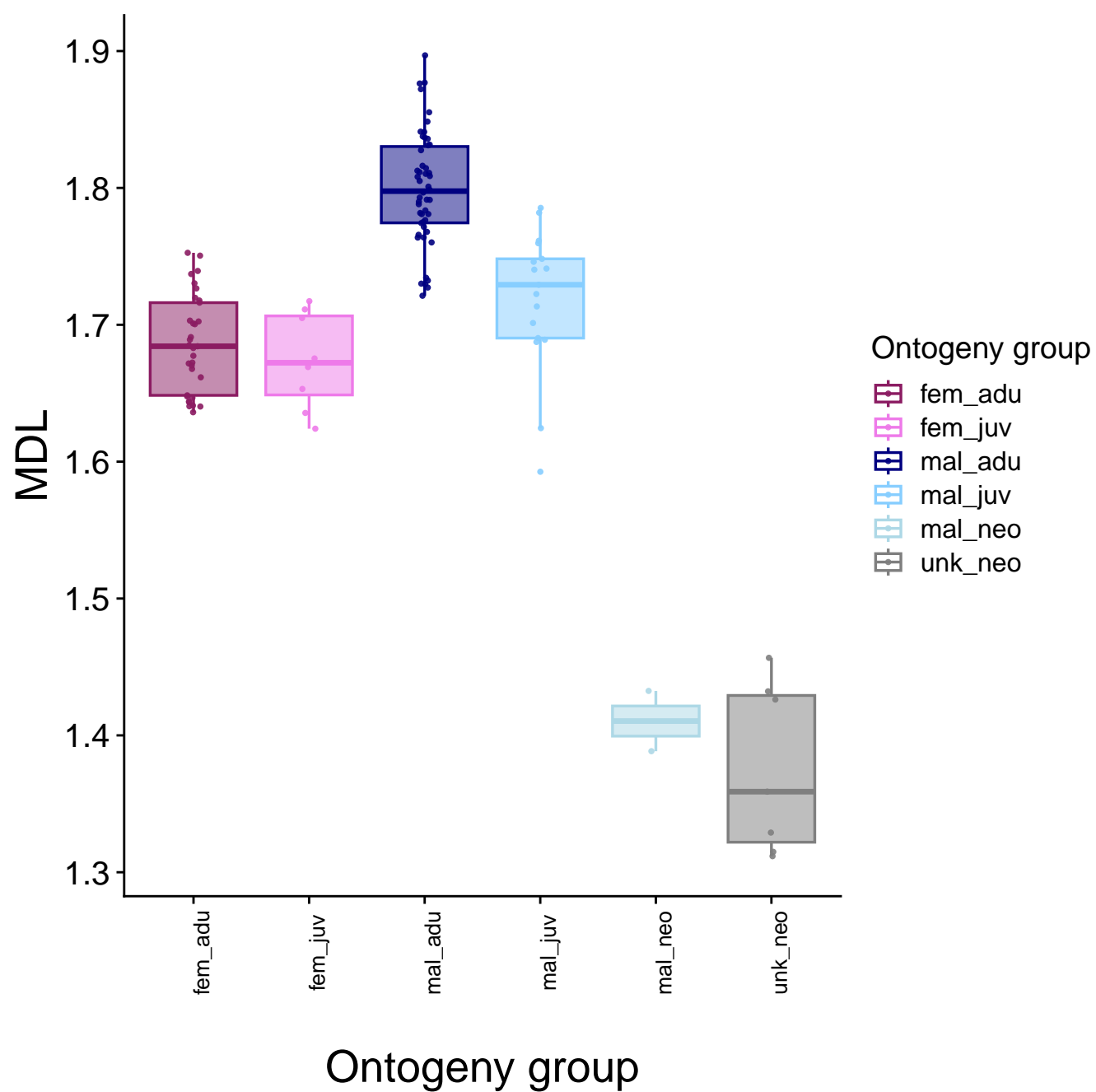

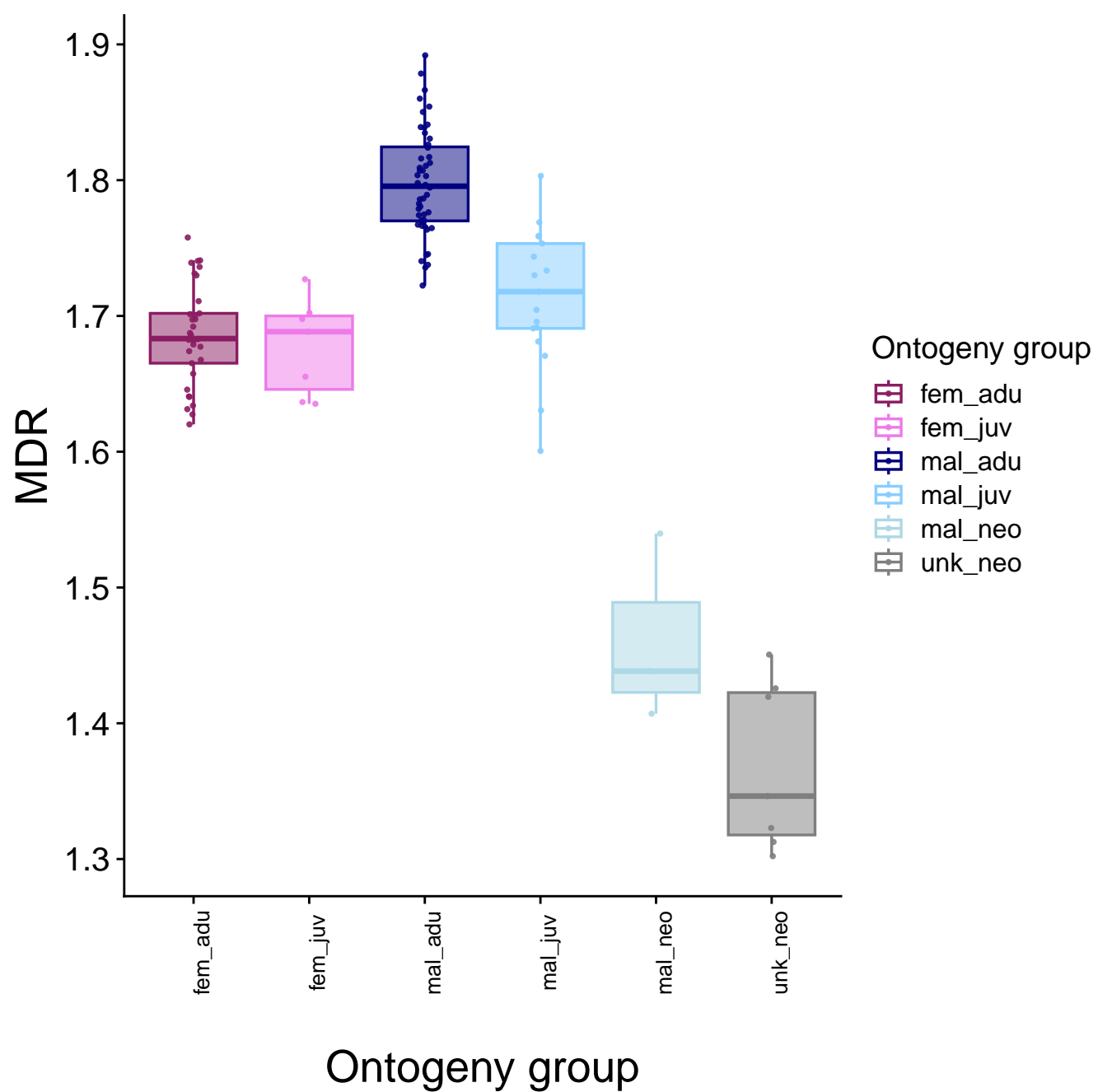

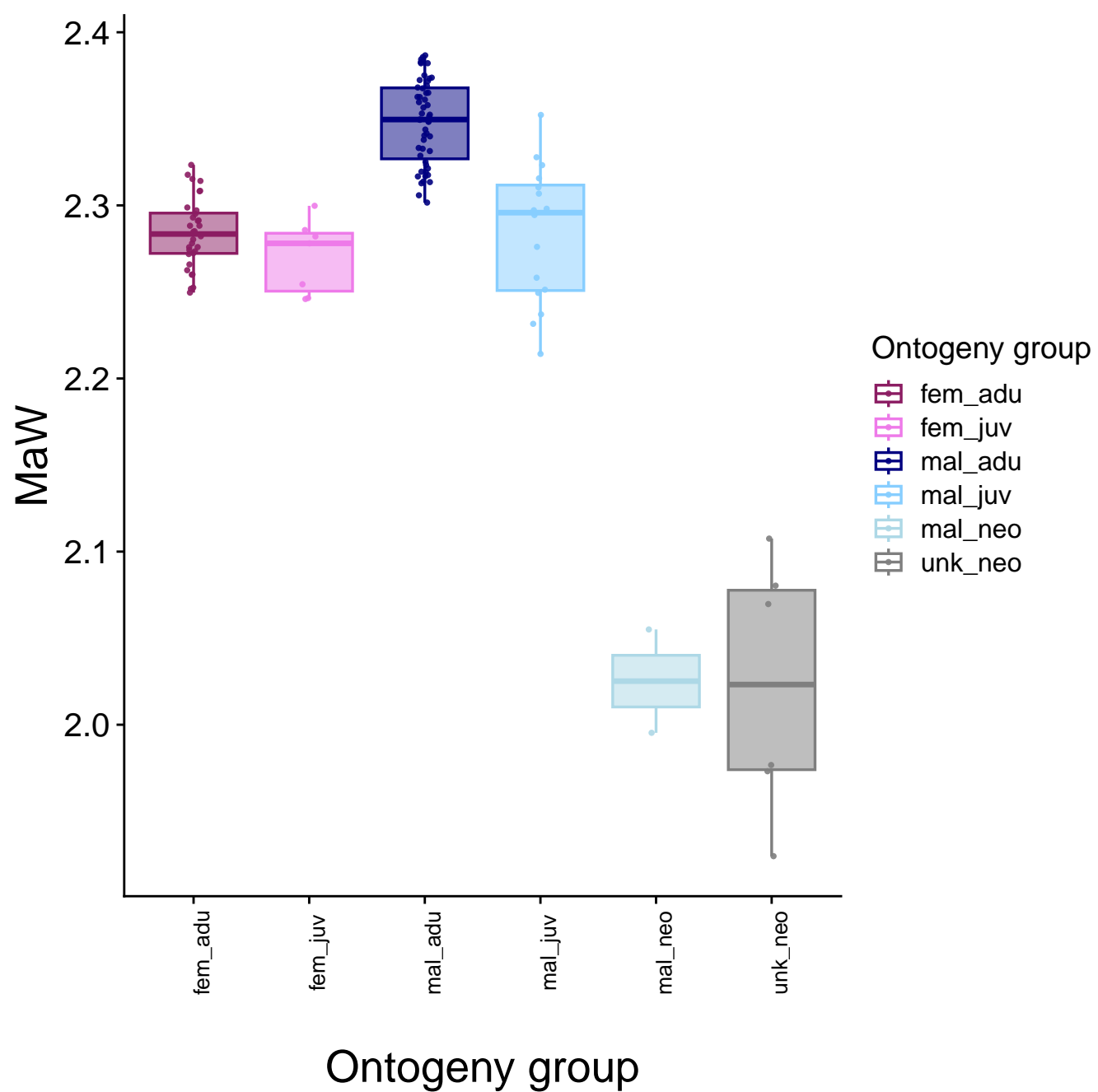

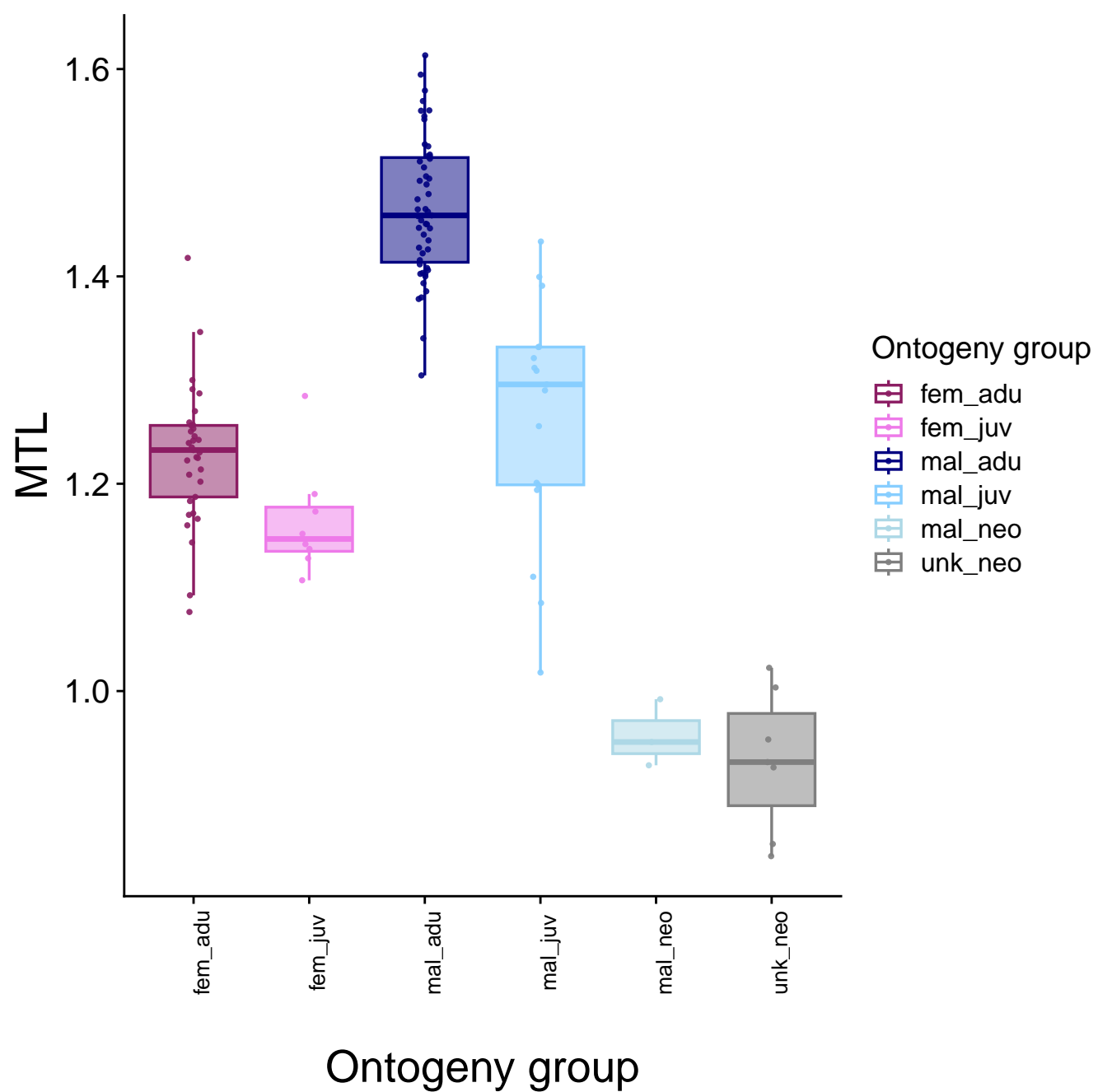

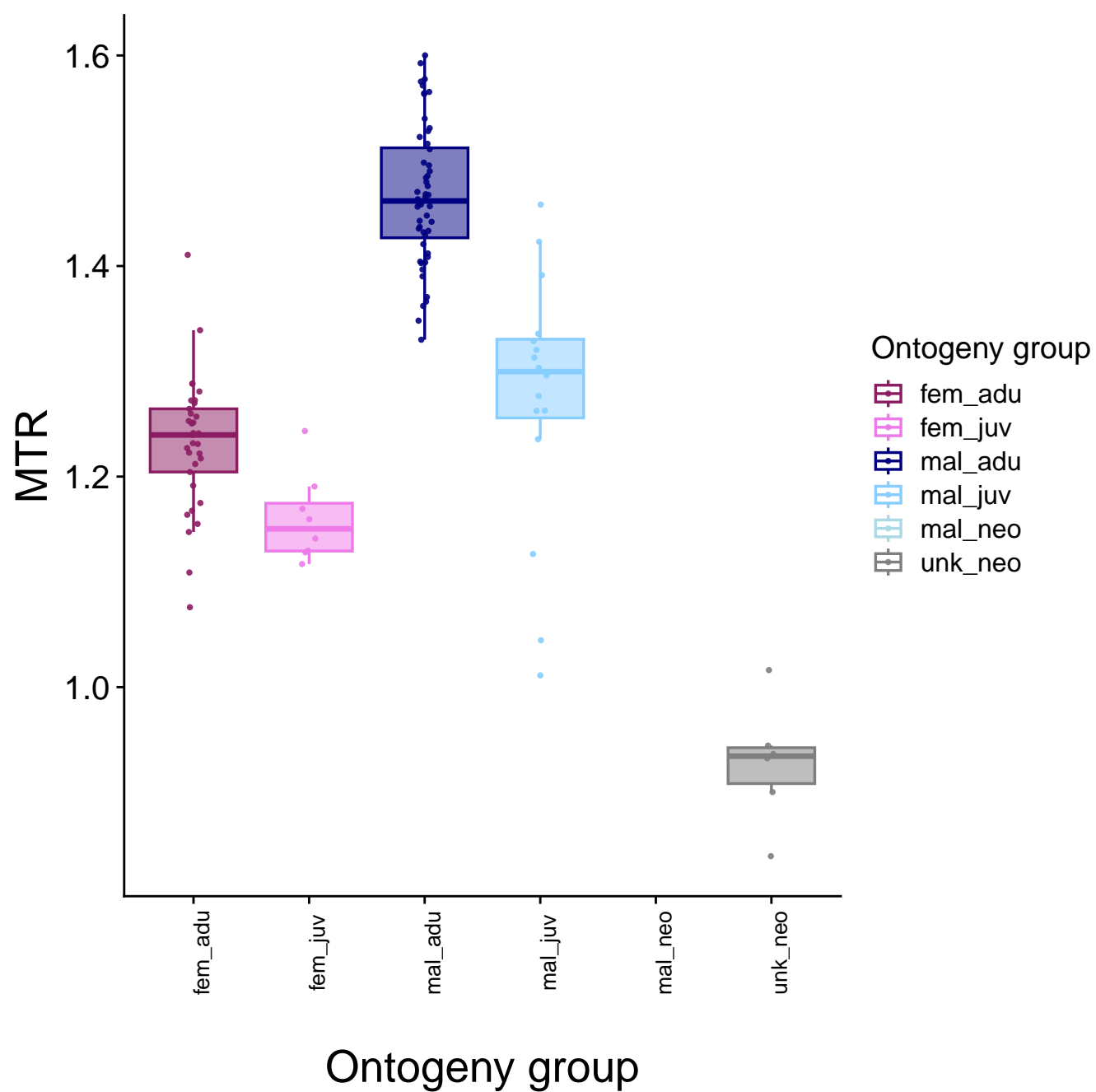

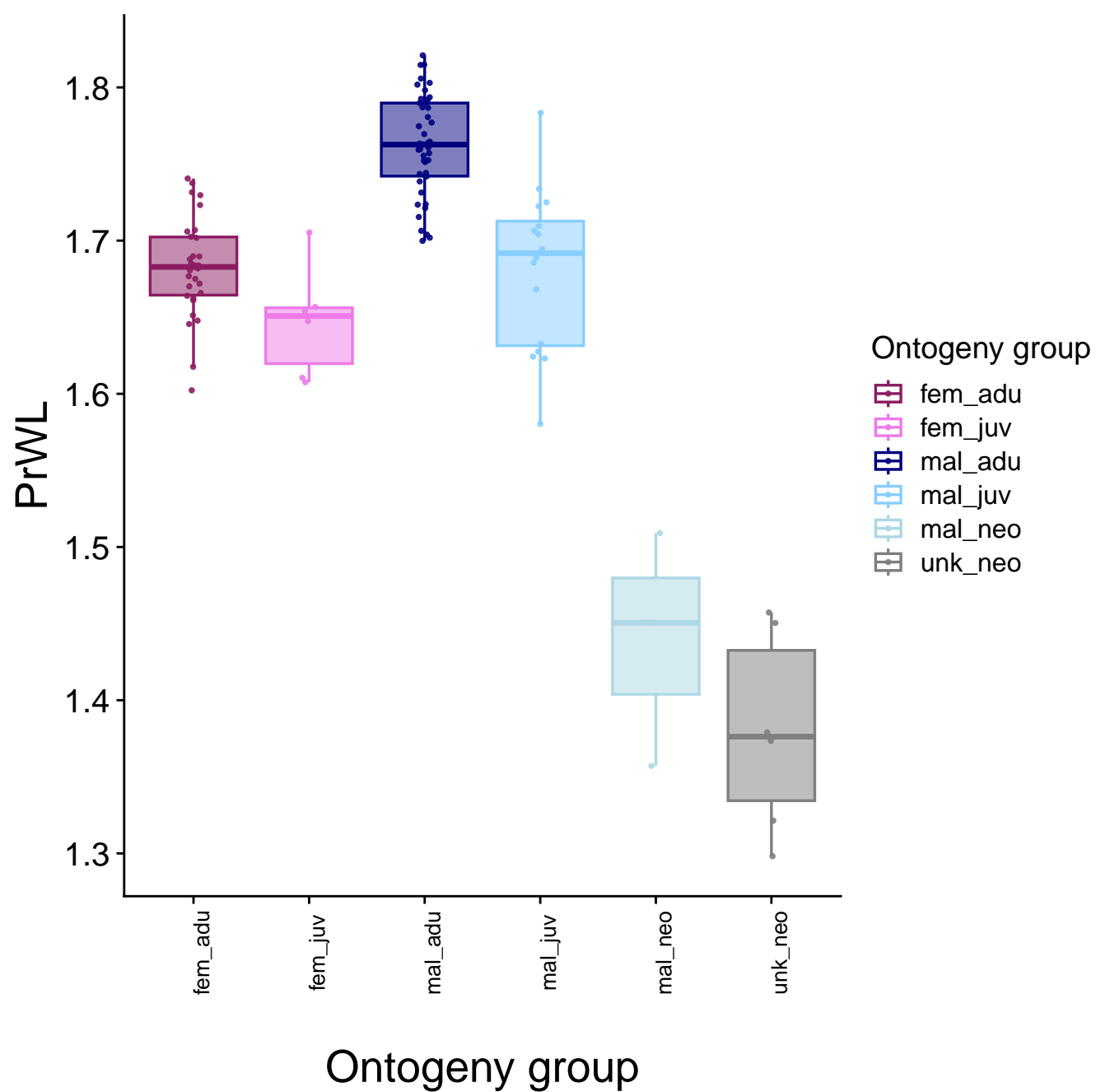

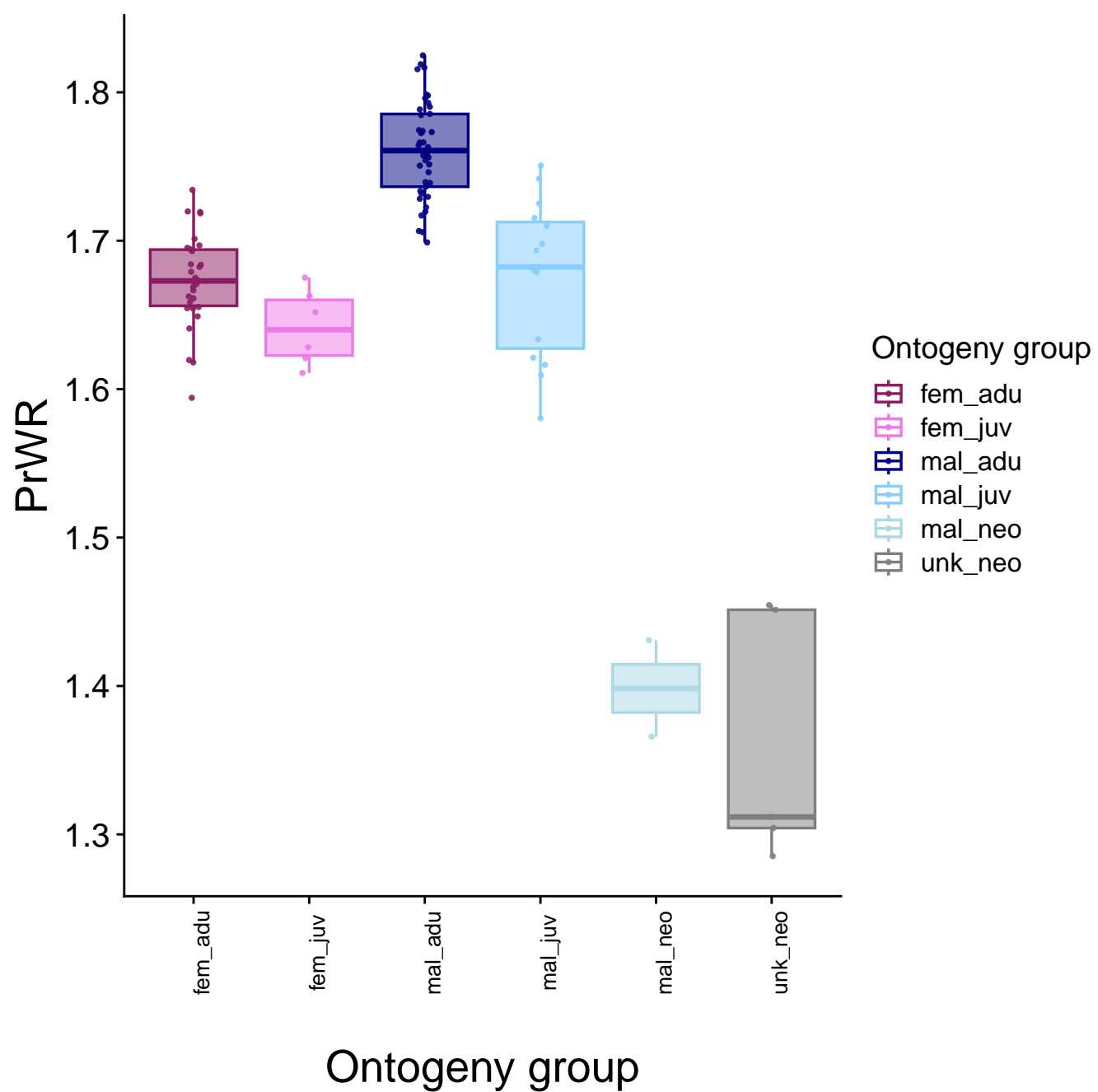

Supplement: Supplementary file 8 — Data S8: Boxplots. [file AR-309-1875-s006.pdf]
